# Selectivity in Chemiresistive Gas Sensors: Strategies and Challenges

**DOI:** 10.1021/acs.chemrev.4c00592

**Published:** 2025-04-08

**Authors:** Peresi Majura Bulemo, Dong-Ha Kim, Hamin Shin, Hee-Jin Cho, Won-Tae Koo, Seon-Jin Choi, Chungseong Park, Jaewan Ahn, Andreas T. Güntner, Reginald M. Penner, Il-Doo Kim

**Affiliations:** † Department of Mechanical and Industrial Engineering, University of Dar es Salaam, P.O. Box 35131, Dar es Salaam, Tanzania; ‡ Department of Materials Science and Chemical Engineering, Hanyang University, Ansan 15588, Republic of Korea; § Department of Materials Science and Engineering, Korea Advanced Institute of Science and Technology (KAIST), 291 Daehak-ro, Yuseong-gu, Daejeon 34141, Republic of Korea; ∥ Advanced Nanosensor Research Center, KI Nanocentury, KAIST, Daejeon 34141, Republic of Korea; ⊥ Human-Centered Sensing Laboratory, Department of Mechanical and Process Engineering, ETH Zürich, CH-8092 Zürich, Switzerland; # Division of Materials of Science and Engineering, Hanyang University, 222 Wangsimni-ro, Seongdong-gu, Seoul 04763, Republic of Korea; ∇ Institute of Nano Science and Technology, Hanyang University, 222 Wangsimni-ro, Seongdong-gu, Seoul 04763, Republic of Korea; ○ Department of Chemistry, University of California, Irvine, Irvine, California 92697-2025, United States

## Abstract

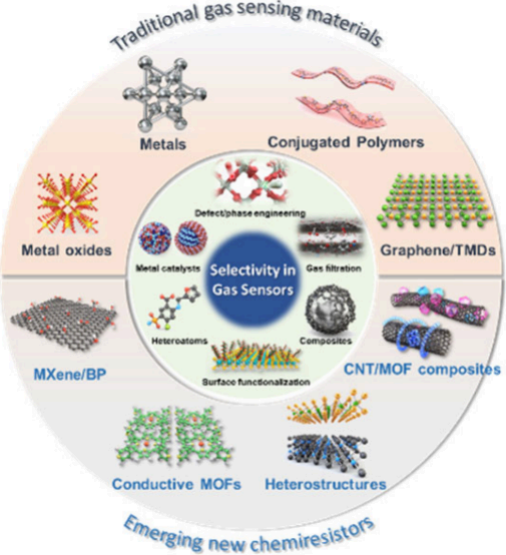

The demand for highly functional chemical gas sensors has surged due to the increasing awareness of human health to monitor metabolic disorders or noncommunicable diseases, safety measures against harmful greenhouse and/or explosive gases, and determination of food freshness. Over the years of dedicated research, several types of chemiresistive gas sensors have been realized with appreciable sensitivities toward various gases. However, critical issues such as poor selectivity and sluggish response/recovery speeds continue to impede their widespread commercialization. Specifically, the mechanisms behind the selective response of some chemiresistive materials toward specific gas analytes remain unclear. In this review, we discuss state-of-the-art strategies employed to attain gas-selective chemiresistive materials, with particular emphasis on materials design, surface modification or functionalization with catalysts, defect engineering, material structure control, and integration with physical/chemical gas filtration media. The nature of material surface–gas interactions and the supporting mechanisms are elucidated, opening opportunities for optimizing the materials design, fine-tuning the gas sensing performance, and guiding the selection of the most appropriate materials for the accurate detection of specific gases. This review concludes with recommendations for future research directions and potential opportunities for further selectivity improvements.

## 1 Introduction

In recent years, our society has developed a pronounced awareness on a wide range of environmental and health issues such as (1) controlled storage of flammable gases, (2) control of toxic gas emissions and reduction of associated human health risks, (3) nondestructive determination of food freshness to safeguard consumers’ health, and (4) prediction of health status of individuals. For instance, rotting foods produce specific gaseous compounds such as amine components, which can give clues on food freshness.^1,2^ Similarly, it has been experimentally shown that metabolic disorders and noncommunicable diseases correlate with the presence of certain volatile organic compounds (VOCs) in human exhaled breath,^3^ rendering the analysis of volatile biomarker complementary to blood and urine assay. Also worth considering is the emission of harmful and greenhouse gases into the environment through leakage, explosion, combustion, or other routes, which poses health threats to humans and the surrounding ecosystem.^4^ When these gases are released and mixed with many other ambient gas molecules, it becomes difficult to selectively detect a target gas. To address this issue, it is important to understand various strategies that have been attempted to attain selectivity toward target gases and the allied mechanisms. To date, various gas sensors employing potentiometric,^5,6^ amperometric,^7^ capacitive,^8^ mass-sensitive,^9^ optical,^10−13^ and chemiresistive^14−17^ operating principles have been widely elucidated in the literature. Herein, we constrain our discussion to selective chemiresistive sensors.

Typically, chemiresistive sensors operate based on the principle of detecting the changes in the electrical conductivity upon exposure of the gas sensing materials to the environment, which may consist of air (or rather, the contained oxygen) or other types of reducing and/or oxidizing gases. Semiconducting metal oxides (SMOs) in pristine forms (such as ZnO, SnO_2_, WO_3_, CuO, Co_3_O_4_, In_2_O_3_, and Fe_2_O_3_), as well as composite and noble metal functionalized forms thereof (such as SnO_2_–ZnO, Pt–SnO_2_, GO–WO_3_, ZnO/ZnCo_2_O_4_, RuO_2_–WO_3_, PdO–ZnO, Pt–ZnO, La–ZnO, Pt–PdO, and Pt–WO_*x*_N_*y*_) represent the most investigated class of chemiresistive materials.^18−26^ The interaction between target gas species and the SMOs is largely based on the interaction with oxygen- or hydroxyl-related species adsorbed on the surface of the SMOs, which will be further explained in the following sections. Besides the SMOs, other materials such as metals, metal alloys/composites, conjugated polymers, carbon nanotubes^27^ and various 2D materials such as graphene and its derivatives, transition metal dichalcogenides (TMDs), nitrides, bromides,^28^ MXenes, phosphorenes, and conducting metal–organic frameworks (cMOFs) have also been suggested as highly promising platforms for gas sensing applications.^29−34^ The gas sensing mechanism of these materials depends usually on direct charge exchange between the sensing layer and the target gases.

**Figure 1:**
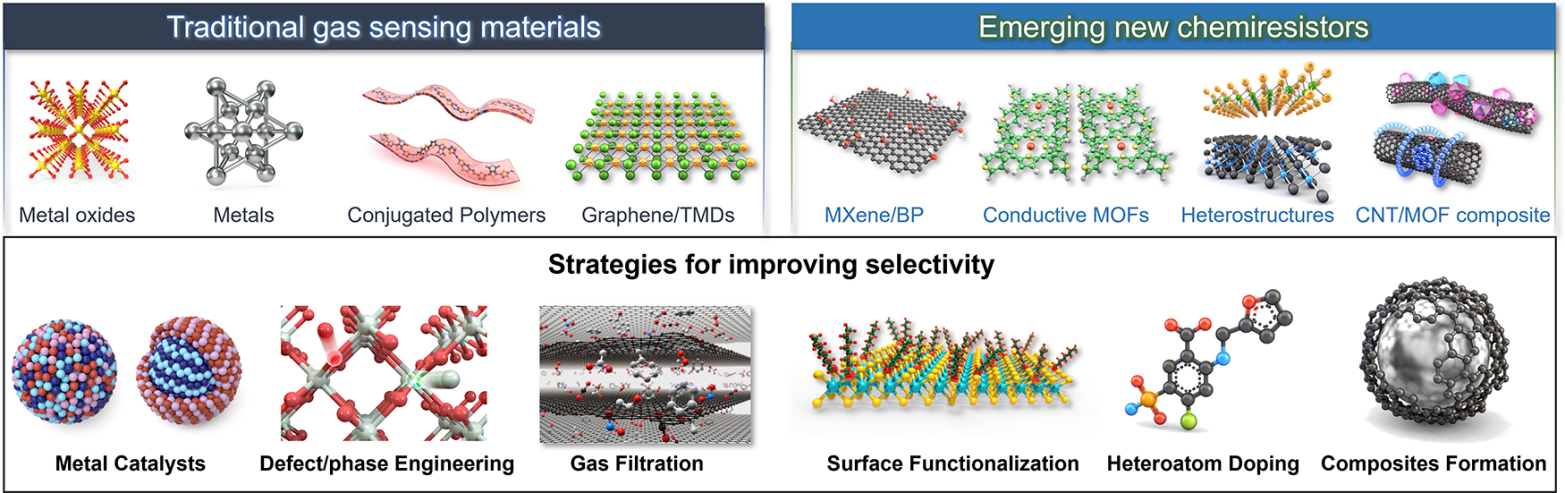
Schematic illustration of traditional and emerging materials for chemiresistive gas sensors, and strategies for improving selectivities toward target gas species.

The chemiresistive sensor operates primarily based on changes in resistance values induced by surface chemical reactions. However, when multiple coexisting gas species simultaneously influence the resistance value, the resulting signal represents a convolution of these individual effects, making it challenging to deconvolute the contributions of specific analytes. In this context, selectivity, as defined by IUPAC, refers to the ability to determine a specific analyte in a mixture or matrix without interference from other components.^35^ Despite reports of specific sensor materials with promising selectivity toward certain analytes, the underlying mechanisms often remain unclear.^36,37^ Overall, the poor selectivity of chemiresistive-type sensors continues to pose a significant challenge for their successful commercialization. Figure 1 illustrates materials widely utilized as chemiresistive gas sensing materials and the common approaches implemented to enhance their selectivity.

Moreover, the assessment of selectivity in gas sensing literature often varies widely, with many studies defining selectivity as the ratio of sensor responses to different target gases. However, such metrics may fail to capture the full complexity of interference effects under realistic environmental conditions, including the presence of humidity, temperature variations, and complex gas mixtures. This lack of standardization complicates direct comparisons across studies and highlights the need for more rigorous and consistent evaluation methods.

In this comprehensive review, we present the recent developments in the novel strategies employed to attain selectivity in chemiresistive-type gas sensors, with a focus on the associated opportunities and challenges. We also provide our assessment of the current progress as well as the future outlook.

## 2 Basic Sensing Mechanism of Chemiresistive Sensors

The three primary principles of a sensor, that is, the receptor function, the transducer function, and the utility factor, have been elucidated in different treatises by several scholars including Yamazoe et al.,^37−40^ Rothschild et al.,^41^ and Barsan et al.^42^ Detailed gas sensing mechanisms of n-type and p-type chemiresistive materials have also been clearly presented in literature.^29,43−47^ In principle, sequential exposure of conventional (n-type or p-type) SMOs to oxygen and target gases leads to a change in electrical resistance. When SMOs are exposed to air, they adsorb oxygen, either in molecular or atomic forms in accordance with Equation 1 and 2.^42^
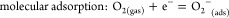
1
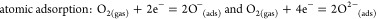
2Adsorption of these oxygen species creates electron-depleted layers (for n-type SMOs) or hole accumulation layers (for p-type SMOs) because of electron trapping by the ionized oxygen from the sensing material.^38,40,42^ Note that with humidity, hydroxyl-related species are adsorbed on the surface. The space-charge regions modulate the potential barrier at intergranular interfaces. Accordingly, the resistance of n-type SMOs increases whereas that of p-type SMOs decreases, as ionosorbed oxygens species develop an electron depletion region in n-type SMOs and a hole accumulation layer in p-type SMOs, respectively. Furthermore, a fluctuation of charge carriers (electrons or holes) occurs when n-type or p-type SMOs and their surface-adsorbed oxygen interact with reducing gases (electron donors) or oxidizing gases (electron acceptors), restoring the n-type and p-type SMOs to their original states in air. Specifically, reducing gases (e.g., CO_2_, acetone, H_2_) donate electrons, detaching ionized oxygen species from the surface, thereby decreasing the electron depletion region in n-type SMOs and increasing the hole accumulation region in p-type SMOs. Conversely, oxidizing gases (e.g., NO, NO_2_, O_3_) attract electrons from the conduction band, further increasing resistance in n-type SMOs and decreasing resistance in p-type SMOs. In some cases, analytes may adsorbed also directly and modulate the space-charge region.^48^ Subsequently, the barrier potential at intergranular interfaces is modulated. Altogether, the transformation of the charge carrier fluctuations into variations in electrical conductivity across the grain boundaries is the basis for the gas-sensing signal. The intensity of the gas sensing signal can be improved through a variety of techniques including surface modifications of pristine SMOs with catalysts,^20,49−51^ enhancement of gas diffusion,^52^ as well as control of grain size and grain–grain interconnections.^37^

It is worth noting that room temperature operating 2D materials such as transition metal dichalcogenide (TMD) and graphene, have a slightly different operating principle because surface charge interaction with adsorbed analytes is more likely to be responsible for the sensing mechanisms instead of the participation of adsorbed O_2_^–^, O^–^, or O^2–^ species.^53^ When 2D materials are exposed to various gases at room temperature, these gases are ionosorbed on the surfaces and accept or donate varying quantities of charge, resulting in modulation of local carrier density and consequently the resistance.^53−55^ Charge transfer mechanisms in 2D materials can be explained by both exchange of charge carriers with physisorbed gases and modulation of Schottky barrier/junctions (for graphene in particular). Exposure of n-type 2D materials to reducing/oxidizing gases decrease/increase both the resistivity and the Schottky barrier, whereas the p-type analogues exhibit an opposite resistance variation.^47^

Understanding the operating mechanisms behind chemiresistive gas sensing requires consideration of not only the transduction process, where the adsorption of gas molecules is converted into a measurable sensor signal, but also the reception of gases on the surface of sensing materials. The surface chemistry and temperature conditions play a critical role in determining how gas molecules interact with the sensor surface, influencing their binding orientation and behavior.^56^ For instance, the same gas molecule can exhibit different adsorption orientations depending on surface properties, and in some cases, (incomplete) thermal decomposition may occur, leading to the formation of intermediate states through multiple reaction steps. These factors directly impact the sensitivity of the gas sensor to specific analytes and, more importantly, the selectivity in differentiating target gases from interfering species.

To analyze and understand these interactions under realistic conditions, a variety of spectroscopic and structural tools are employed. Diffuse reflectance infrared Fourier transform spectroscopy (DRIFTS),^57−60^ Raman spectroscopy,^61,62^ X-ray absorption spectroscopy,^63^ and X-ray diffraction (XRD)^64^ are particularly valuable, as they can be applied under *in operando* conditions. These techniques provide critical insights into the dynamic surface reactions and intermediate states formed during gas adsorption, enabling a more comprehensive understanding of the factors governing sensor performance and selectivity.

Humidity, or the presence of water vapor, is a ubiquitous environmental factor that significantly influences the performance of gas sensors. Unlike specific target analytes, water vapor is not typically considered an interferent in the same sense but rather an inherent aspect of the operational environment. Its effects are particularly pronounced in semiconducting metal oxide-based sensors, where adsorption and desorption of water molecules can alter the baseline resistance, interfere with charge transfer processes, and introduce variability sensor response.^65−67^ Addressing humidity-related effects is essential for ensuring sensor stability and reliability under diverse ambient conditions. Strategies such as humidity-resistant material coatings, controlled operating conditions, or integration with desiccating filters have been developed to mitigate these effects, which will also be introduced in the following sections. By emphasizing the role of water vapor as an environmental factor rather than an analyte, we aim to highlight the importance of designing sensors that can robustly operate in realistic, variable conditions without compromising their selectivity or sensitivity to target gases.

## 3 Selectivity in Semiconducting Metal Oxides-Based Chemiresistors

SMOs-based chemiresistive sensors are valued for their simple operation principles and robust performance but suffer from poor gas selectivity. This limitation arises because the sensing mechanism in unmodified SMOs is primarily based on charge carrier modulation, which lacks specificity to particular gases, which remains a key challenge in their broader adoption.^68,69^ For n-type SMOs, chemisorbed oxygen forms an electron depletion region, with reducing gases decreasing resistance and oxidizing gases increasing resistance; p-type SMOs exhibit the opposite behavior via hole accumulation. However, these responses are broad and nonspecific. Furthermore, SMO sensors require high operating temperatures (200–450 °C) to activate surface reactions, which reduces selectivity as many gases become reactive under these conditions. Enhancing selectivity requires strategies like catalytic nanoparticle incorporation, heterojunction design, or external stimuli such as light activation, as highlighted by numerous studies emphasizing the importance of tailoring sensor responses to specific analytes to improve selectivity. However, there are exceptions to this behavior for cases where selectivity mechanisms for particular gases are influenced by a reversible phase change of SMOs which follow a unique reaction with target gases. In this regard, metal oxides such as CuO, NiO, and WO_3_ have attracted immense attention for the detection of hydrogen sulfide (H_2_S). Apart from the basic sensing mechanism, CuO,^70,71^ NiO,^72,73^ and WO_3_^74^ react directly with H_2_S according to eqs 3, 4, and 5, respectively.
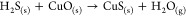
3
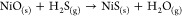
4
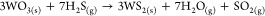
5The as-formed metallic sulfides (CuS, NiS, and WS_2_) exhibit high electrical conductivity compared to SMOs, leading to a substantial reduction in resistance. Inspired by this mechanism, Kneer et al. demonstrated selective detection of H_2_S using CuO.^71^ Typically, adsorption of H_2_S on the surface of CuO causes the substitution of lattice-bound oxygen by sulfur (S), forming Cu–S bonds that enable conduction of electrons. This selectivity of CuO toward H_2_S is particularly predominant at low operating temperatures (<200 °C), whereas at higher operation temperatures, electron excitation and recombination with holes are likely to occur, favoring the reaction of H_2_S with adsorbed oxygen (eq 6) as a dominant reaction.^75^
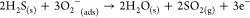
6In this way, the selectivity of CuO toward H_2_S tends to decrease a little at high operating temperatures. It has been observed that, at room temperature, the reaction of H_2_S with adsorbed oxygen is also predominant for concentrations of H_2_S exceeding 100 ppm, whereas for concentrations in the range of 1–100 ppm, the formation of CuS predominates.^76^ For the case of WO_3_, Shi and co-workers demonstrated that immobilization of hexagonal WO_3_ (h-WO_3_) nanosheets onto reduced graphene oxide (rGO) increased the response to about 3.7–folds toward 40 ppm of H_2_S.^74^ The sensor exhibited fast response (7 s) and recovery (55 s) to 10 ppm of H_2_S, and a low limit of detection (LOD) of 10 ppb. The improvement in selectivity was attributed to the unique reactivity of WO_3_ toward H_2_S, as opposed to that of interfering gases which could potentially reduce W^6+^ to W^4+^. Moreover, this unique reactivity of CuO, NiO, and WO_3_ with H_2_S make them ideal catalysts for H_2_S detection when incorporated in other SMOs.^73,77^ Qu et al.^72^ hydrothermally grew NiO/ZnO nanowires (NWs) and investigated their room temperature sensing performance toward H_2_S. A higher response of 31.5% toward 100 ppm of H_2_S was observed with a faster response rate (15 s), unlike bare ZnO NWs analogues. When H_2_S was removed and dry air (O_2_) was reintroduced, NiO would be reformed as shown in eq 7, demonstrating a reversible interaction of NiO with H_2_S. This reversibility can also be observed for interactions of H_2_S with CuO and WO_3_ in accordance with eqs 8 and 9.
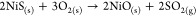
7
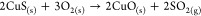
8
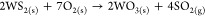
9Typically, the formation of metal sulfides leads to the destruction of p–n or n–n junctions at interfaces of p-type/n-type or n-type/n-type oxides, respectively, significantly modulating the resistance of SMOs.

As discussed hitherto, CuO, NiO, and WO_3_ based sensors exhibit a much-improved selectivity toward H_2_S. However, these are very specific examples of material–gas pairs that show such selectivities, and their capability to discriminate interfering gases is still limited when exposed to target gases other than H_2_S. Therefore, as a more practical set of approaches to develop a general strategy for achieving noticeable improvements in the selectivity of SMOs, we present a discussion in the following sections regarding the (1) functionalization of SMOs with catalysts, (2) defect generation and structure control, and (3) integration of SMOs with gas filters.

### 3.1 Toward Commercialization of Semiconducting Metal Oxides-Based Chemiresistors and Current Limitations

Semiconducting metal oxides (SMOs) are a key contributor to modern gas sensing technology and dominate chemiresistive sensors. As these materials transition from laboratory research to industrial applications, their widespread adoption highlights their critical role in sectors such as environmental monitoring (e.g., Figaro TGS 2600, AMS CCS811, or Sensirion SGP30), industrial safety (e.g., AMS CCS801, CCS803, or SGX MiCS-5524), and medical diagnostics (e.g., Bosch Vivatmo *me*). SMO-based chemiresistive gas sensors boast several decades of real-world application, standing out as a cost-effective option^78−80^ compared to other sensor types like electrochemical or photoionization detectors. The primary targets for these sensors are usually VOCs, H_2_, CH_4_ CO, and NO_*x*_, which are key contributors to outdoor and indoor air pollution.^81,82^ Since these often go undetected by human senses, monitoring tools are required, e.g., in chemical industries, hospitals housing,^83^ and even during space missions.^84^ SnO_2_ (pristine or functionalized) is particularly prevalent in commercial applications in the detection of these gases due to its high sensitivity to various gases. The performance of these commercialized sensors is rigorously evaluated over long periods, typically exceeding 12 months, with findings frequently documented in scientific literature.^85−88^ For instance, Massok et al. conducted a comparative study of two Figaro sensors,^88^ the Figaro TGS 813 and the TGS 842, assessing their sensitivity, selectivity, and long-term stability in methane detection. Their research revealed that the TGS 842, the most recent model at the time, exhibited enhanced selectivity toward methane compared to the TGS 813. Yet, inaccuracy of such sensors remains a critical concern,^89^ that may be overcome with the strategies outlined in this review article. For instance, it was noted that the TGS 842 still displayed some cross-sensitivity to propane, a challenge due to both gases belonging to the same family of hydrocarbons.

The relentless pursuit of innovation in sensor technology, spurred by the burgeoning Internet of Things (IoT),^90^ is reshaping the landscape of smart and wearable devices globally. This revolution demands smarter sensor solutions, particularly for applications in pollution monitoring and healthcare, where the size and power consumption of sensors are critical for integration into mobile and hand-held or wearable technologies. In response to these needs, companies like Sensirion, Bosch, and Honeywell are developing advanced gas sensing platforms that utilizes microelectromechanical systems (MEMS) technology, featuring highly miniaturized semiconducting metal oxide (SMO) gas sensors and microheaters that operate with ultralow power consumption. These innovations are crucial for applications requiring compact, energy-efficient sensors that do not compromise on performance.^91^ A pertinent study by Hong et al. underscores the challenges and opportunities presented by these advanced sensors. Their research evaluated the Sensirion SGP30 sensor in a field test for VOC detection across three distinct regions in Taiwan, spanning 10–12 months.^86^ The study revealed that environmental variables such as relative humidity, temperature, and CO concentrations significantly influenced the sensor readings. Notably, cross-sensitivity to CO was identified as a primary factor in the sensor’s tendency to overmeasure VOC levels. These findings highlight the necessity for ongoing calibration of the sensors, taking into account hourly fluctuations in temperature, relative humidity, and CO levels to enhance accuracy. Such adjustments are essential for ensuring that these advanced sensors can reliably function in diverse environmental conditions, making them more adaptable for IoT applications in real-world settings.

Achieving selectivity even in commercialized SMO-based chemiresistive gas sensors poses significant challenges. Typically, these sensors tend to detect multiple analytes nonselectively, as they often exhibit similar response characteristics to various substances. However, a more targeted approach involves designing a gas sensing layer specifically tailored to detect a particular analyte, which can significantly enhance selectivity. A notable advancement in this direction was made in 2021 through a collaborative effort between materials scientists from a research institute and electrical engineers from Samsung Electronics.^92^ This team developed a medical device designed to diagnose halitosis (bad breath) by directly analyzing human breath. The core of this device is its innovative gas sensing layer, comprised of WO_3_ nanofibers. These nanofibers are functionalized with Pt nanoparticles and a sodium-induced secondary phase of Na_2_W_4_O_13_. This composition was specifically engineered to detect hydrogen sulfide (H_2_S), a biomarker for halitosis, demonstrating exceptional selectivity (with a response ratio of H_2_S to ethanol (*R*_EtOH_) of 277), sensitivity, and rapid response times (∼10 s to 1–5 ppm of H_2_S). Moreover, the sensor demonstrates distinct cross-selectivities toward dimethyl sulfide (DMS) and methyl mercaptan (CH_3_SH), two additional volatile sulfur compounds associated with halitosis, with respective response ratios (*R*_H2S_/*R*_DMS_ and *R*_H2S_/*R*_CH3SH_) of 9.5 and 2.7 at 1 ppm. These values underline the sensor’s ability to differentiate between these sulfur-containing species and other interfering compounds. To further refine its performance, the device integrates additional sensors for humidity, pressure, and temperature. This multivariate analysis allows for a comprehensive assessment of the breath sample, enhancing the device’s ability to quantitatively determine hydrogen sulfide concentrations. Impressively, when compared to traditional gas chromatography methods, this integrated sensor system achieves an accuracy rate of 86.3% in detecting halitosis in 80 case studies with exposure to direct human breath.

Another promising strategy to improve the selectivity of SMO-based gas sensors is the integration of a filtering mechanism^93,94^ prior to analyte detection. This approach complements the direct modifications of the SMO layer by adding a selective filtration step that can screen out interfering gases before they reach the sensor. An illustrative example of this technique was developed by van den Broek et al., who engineered a low-cost, hand-held sensor platform incorporating a separation column filled with Tenax.^95^ This polymer-based sorption filter is designed to selectively detect methanol, effectively differentiating it from other interfering gases. The detailed mechanics of this design will be explored further in section 3.4.3 of this review. Building on this foundational research, the same team designed a device specifically tailored for the detection of toxic methanol in alcoholic beverages (e.g., distillates),^97^ hand sanitizers^98^ and even exhaled human breath.^99^ The device leverages a Pd-doped SnO_2_ sensor combined with the Tenax filter to enhance selectivity and accuracy, and performs automated temperature correction to enable stable performance under varying conditions.^100^ In proof-of-concept tests, it quantified methanol concentrations ranging from 0.01 to 10 volume percent,^101^ as confirmed also in an interlaboratory trials with 17 independent participants following ISO 5725.^100^ Moreover, field tests confirmed that the sensor performance aligns closely with that of conventional gas chromatography coupled with a flame ionization detector,^97^ validating its practical efficacy.

Looking ahead, future research should focus on optimizing sensor architectures and exploring new material combinations to overcome current limitations. Continued interdisciplinary efforts will be vital in advancing sensor technology to meet the increasing demands of industrial, environmental, and health-related applications, ultimately leading to more reliable and user-friendly sensing solutions.

### 3.2 Functionalization of Semiconducting Metal Oxides with Catalysts

#### 3.2.1 Noble Metal Nanoparticle Catalysts

Metal nanoparticles (NPs), particularly those based on noble metals such as palladium (Pd), platinum (Pt), silver (Ag), rhodium (Rh), and gold (Au), exhibit remarkable electronic properties and catalytic activity toward various gases when incorporated in or on surfaces of SMOs.^15,16,19,25,102−104^ The influence of these catalysts is not only reflected in the enhancement of the sensitivity, the acceleration of the sensing speed (response and recovery times) and lowered sensor operational temperature, but also in the improved selectivity of the sensor. Chiefly, the gas-selective property can be influenced in two different ways: catalysts are either highly reactive to analyte gases,^105^ or in some cases, their work functions are relatively low, enabling the adsorption of specific gases (e.g., H_2_ on Pd–Zn_2_SnO_4_) as opposed to the interferents.^103^ A recent review by Degler et al. has highlighted the critical influence of metal nanoparticle loading on both chemical and electronic sensitization mechanisms of semiconducting metal oxides.^106^ Key parameters such as the size, coverage, and distribution of loaded nanoparticles play pivotal roles in determining gas sensing performance, particularly through their effects on oxygen spillover, catalytic activity, and lattice oxygen activation at the interface. While much of the current research primarily focuses on improving performance, understanding the interplay between these mechanisms also offers fundamental insights into how nanoparticle tuning can be leveraged to enhance gas selectivity. For instance, selective reactions on additive phases, such as Rh_2_O_3_-loaded WO_3_ for CO sensing,^107^ or CuO-loaded SnO_2_ for H_2_S detection,^108^ directly modulate the space charge layer and sensor response.

Regarding the first mechanism, interfering gases would be spontaneously consumed on the surface of the sensing layer to minimize the ingress of the interfering gases into the bulk sensing material. Meanwhile, a less-reactive gas (the target gas, in this case) would favorably permeate the sensing layer. Among such catalysts is Au, which shows high catalytic activity toward oxidation of combustible gases. For example, one report showed that, upon loading small Au NPs on ZnO NPs (pore size >10 nm), the material not only exhibited an extremely high response to 100 ppm toluene (*R*_a_/*R*_g_ = 92) at 377 °C but also showed high selectivity against interfering gases such as H_2_, CO, ethanol, acetaldehyde, and acetone (*R*_a_/*R*_g_ < 7 at 100 ppm) due to the high catalytic activity of Au catalyst NPs toward these gases.^105^ However, a higher amount of Au would also sharply degrade the selectivity and sensitivity toward toluene, indicating that superior selectivity can only be achieved with optimal amount of Au NPs.

The second mechanism relates to chemical sensitization and spillover effects.^14,18^ Du et al. investigated the NO_2_ sensing behavior of Pt-modified SnO_2_ mesoporous spheres and reported an unprecedented ultrahigh response (*R*_g_/*R*_a_ = 5770) and selectivity toward 5 ppm of NO_2_ at 80 °C.^109^ The interfering gases (H_2_S, SO_2_, CH_4_, formaldehyde, and CO_2_) exhibited ultralow responses, even at a concentration of 50 ppm. This sensing behavior resulted from a strong interaction of Pt NPs with NO_2_; that is, the Pt NPs (≈ 5 nm) not only increased the O^2–^/O_2_^–^ ratio on the SnO_2_, but also the selectivity toward NO_2_. It is important to note that NO_2_ is highly hydrophilic and possesses a higher electron affinity (220 kJ/mol) than oxygen (20 kJ/mol).^102^ As a consequence, adsorption of NO_2_ on Pt NPs and subsequent spillover effect would render the displacement of most preadsorbed O^–^ species on SnO_2_ surface, a process which does not occur with the interfering gases.

**Figure 2:**
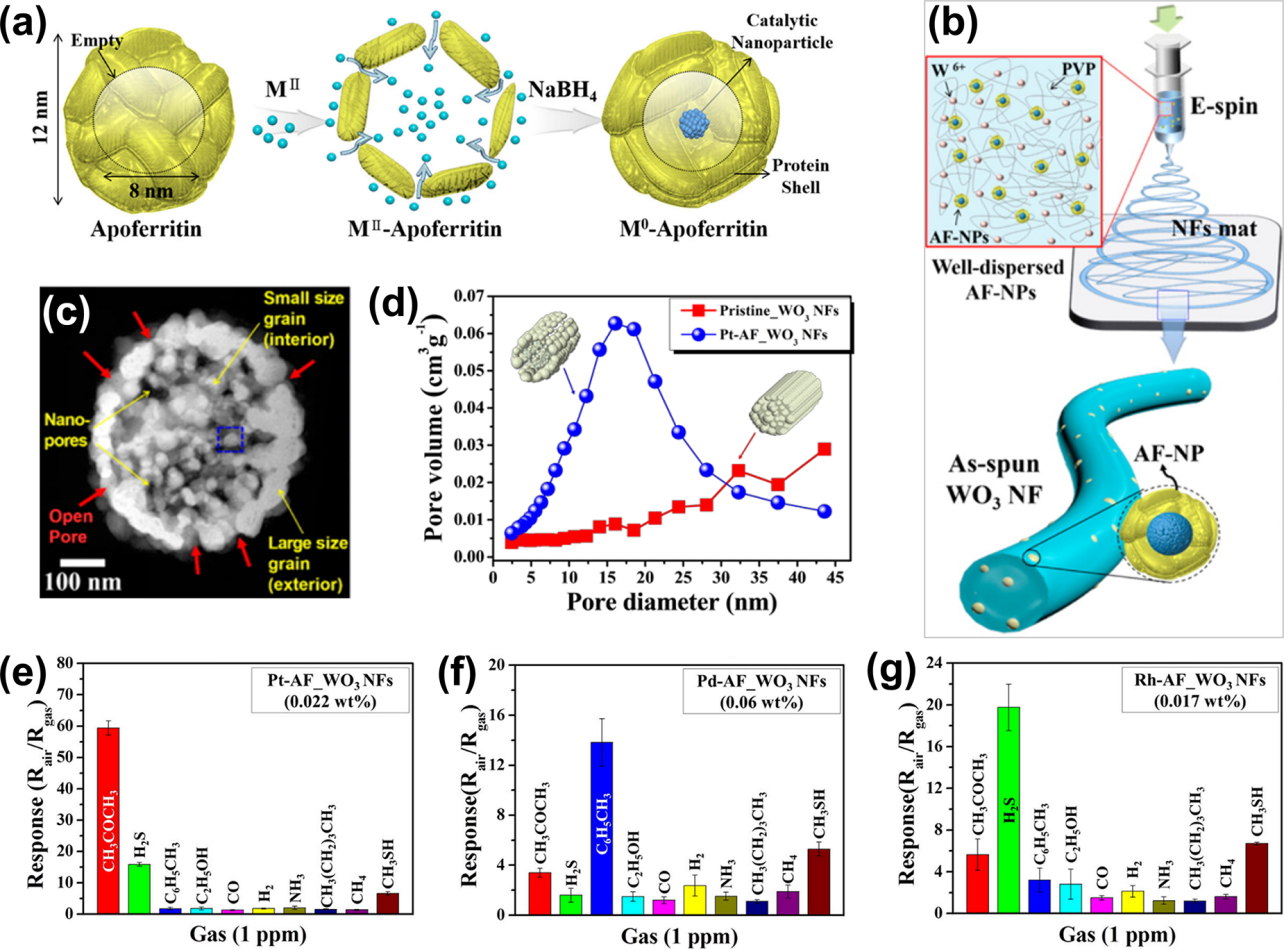
(a) Schematic for synthesis procedure of apoferritin-templated metal catalysts, where M = Pt, Pd, and Rh, (b) schematic for electrospinning of the catalyst-containing precursor solution, (c) morphology of Pt-AF_WO_3_ NFs, and (d) pore distribution of pristine and catalyst loaded WO_3_ NFs. Selectivity of (e) Pt-functionalized WO_3_ NFs (Pt-AF_WO_3_ NFs), (f) Pd-functionalized WO_3_ NFs (Pd-AF_WO_3_ NFs), and (g) Rh-functionalized WO_3_ NFs (Rh-AF_WO_3_ NFs) upon exposure to various gases at 350 °C. Reproduced with permission from ref (16). Copyright 2016 American Chemical Society.

Moreover, this chemical sensitization mechanism can remarkably amplify the response and selectivity of pure SMOs especially when the extremely small catalysts are uniformly incorporated in the SMOs. To this end, Kim et al.^16^ demonstrated the preparation of apoferritin (hollow protein cage with inner cavity size of 8 nm and shell size of 12 nm)-templated catalyst NPs of Pt (2.0 ± 0.58 nm), Pd (1.8 ± 0.71 nm), and Rh (2.0 ± 0.52 nm), which were uniformly decorated on mesoporous WO_3_ nanofibers (NFs). In a typical experiment, the apoferritin molecules were incubated in inorganic precursor solutions under a pH-controlled, reducing environment, during which the apoferritin cavity served as a spatially restricted nanoreactor which enabled the fabrication of the ultrasmall NPs within the cavity (Figure 2a). As illustrated in Figure 2b, the apoferritin-templated NPs exhibited a uniform dispersion in the electrospinning solution and as-spun NFs due to the positively charged apoferritin shell. The thermal treatment of as-spun NFs resulted in highly porous oxide NFs, as both the apoferritin and the templating polymer were removed by thermal decomposition (Figure 2c,d). These findings verify that abundant mesopores could be derived by removing the apoferritin shell, promoting the accessibility of the analyte gases to the NPs. Gas sensing results indicated a notable selectivity toward 1 ppm of acetone, toluene, and H_2_S at 350 °C for Pt-, Pd-, and Rh-functionalized WO_3_ NFs, respectively (Figure 2e–g). Specifically, Pt catalysts increased the adsorption of oxygen and acetone onto WO_3_ via chemical sensitization, i.e., adsorption and subsequent spillover effects,^37^ resulting in a considerable resistance change. Unlike Pt catalysts, Pd and Rh act as the best electronic sensitization among noble metal catalysts.^110^ Accordingly, the oxidation states of Pd and Rh were changed upon the adsorption of oxygen, resulting in the electron transfer from the catalyst NPs to WO_3_, which accelerated the response rate toward toluene and H_2_S, respectively.

Additionally, the catalytic influences of NPs also vary depending on the properties of host SMOs. Different from the previous case study on WO_3_-based systems, Kim et al.^111^ demonstrated the decoration of SnO_2_ NWs with uniformly sized Pt (≈8 nm), Pd (≈6 nm), and Au (slightly elongated, ≈10 nm) NPs, which enabled a fast and selective response to 1 ppm of toluene (*R*_a_/*R*_g_ = 40), benzene (*R*_a_/*R*_g_ = 25.5), and CO (R_a_/*R*_g_ = 20.1) at 300 °C, respectively. The catalytic capability of Pt, Pd, and Au NPs was attributed to both the electronic sensitization (a flow of electrons from SnO_2_ to the NPs) and the chemical sensitization. The chemical sensitization was significantly influenced by both the energy of gas adsorption onto NPs and the desorption temperature. For instance, it was shown that benzene would desorb from Au and Pt NPs at temperatures as low as 127 and 227 °C, respectively. Note that the desorption of benzene from Pt NPs at 227 °C would occur before the dehydrogenation of benzene into phenyl species (C_6_H_*x*_). In this way, the effect of benzene was minimal for the case of Pt-SnO_2_ NWs at 300 °C compared to the case of Pd-SnO_2_ NWs. Among the tested gases (benzene, CO, H_2_S, toluene, and ethanol), only the benzene exhibited a strong interaction with Pd NPs. It is because the adsorption energy (1.35 eV) of C_6_H_6_/Pd is in between those of the other two metal couplets, i.e., 1.49–1.83 eV for C_6_H_6_/Pt and 0.64 eV for C_6_H_6_/Au, thus the Pd NPs might feature higher catalytic activities based on volcano theory.^112^ Another observation was that the reaction barrier of the adsorption of CO on Au NPs was the lowest among the tested gases (i.e., 0.21 eV on Au vs 0.87 and 1.2 eV on Pd and Pt, respectively), which explained the selective CO adsorption on Au-SnO_2_ NWs.

Apart from noble metal catalysts, transition metals are also promising as catalysts for selective oxidation of diverse gases, so long as they are judiciously incorporated into SMOs.^113−115^ In a case study, Qu et al. synthesized ZnO/Ni_0.9_Zn_0.1_O double-shelled nanocages (DSNCs) derived from metal organic frameworks (MOF) for xylene sensing and investigated the catalytic effect of Ni.^115^ We clearly note that the ZnO was the interior shell and the Ni_0.9_Zn_0.1_O was the exterior shell. It was shown that, upon exposure to 100 ppm of xylene at 240 °C, the response of ZnO/Ni_0.9_Zn_0.1_O DSNCs (*R*_a_/*R*_g_ = 54.7) was higher than that of pristine ZnO nanocages (NCs). The Ni in the shell of NCs efficiently dissociated the xylene into more reactive gases. This was not the case with interfering gases like toluene, acetone, ethanol, methanol, NO_2_, and NH_3_. The improved response and selectivity toward xylene were attributed to chemical and electronic sensitization effects as well as the defects that were formed in the synthesis process. However, the exact mechanism for the enhanced selectivity with the increased doping level of Ni could not be clarified based on the limited experimental data.

**Figure 3:**
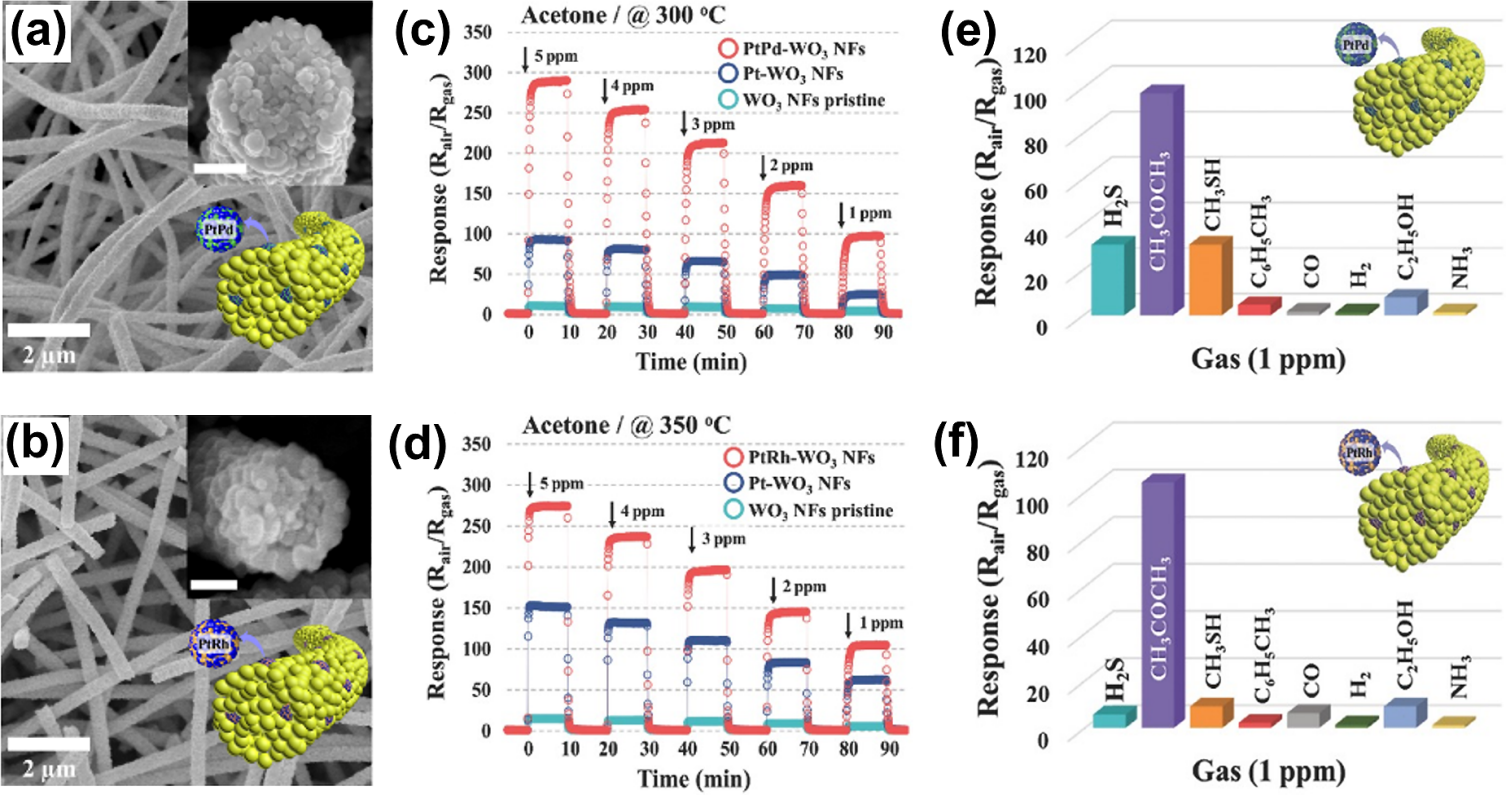
Morphology of (a) PtPd-WO_3_ NFs and (b) PtRh-WO_3_ NFs. The insets are schematic representation and high-resolution images of the NFs. Unindicated scales bars are 200 nm. Acetone sensing property of (c) PtPd-WO_3_ NFs at 300 °C, (d) PtRh-WO_3_ NFs at 350 °C in the concentration ranges of 1–5 ppm. Selectivity property of (e) PtPd-WO_3_ NFs and (f) PtRh-WO_3_ toward 1 ppm of various gases at 300, and 350 °C, respectively. Reproduced with permission from ref (118). Copyright 2017 Wiley-VCH.

Furthermore, the a priori control of composition and structure improves the reactivity and selectivity properties of catalytic NPs. In general, multimetallic NPs with sizes on the subnanometer scale exhibit distinctively improved physical and chemical properties compared to their single-component counterparts.^116^ The robustness of these heterogeneous NPs originates from synergistic effects between the neighboring atoms, which promotes enhanced electronic and catalytic efficacy. Hitherto, several studies have employed multimetallic catalytic NPs in sensing materials.^117−120^ For instance, Kim et al.^118^ prepared an ensemble of porous WO_3_ NFs loaded with ultrasmall apoferritin-templated PtPd and PtRh catalytic NPs (Figure 3a,b). The PtPd and PtRh loaded NFs exhibited higher response to concentrations of acetone in the range of 1–5 ppm (Figure 3c,d), even though the total mass of NPs loaded onto the NFs were significantly lower than that of the monometallic counterparts.^16^ As shown in Figure 3e,f, remarkable selectivity toward 1 ppm of acetone against interfering gases was also observed. Fast dissociation of acetone on the surfaces of WO_3_ and bimetallic NPs enabled response times of 4.2 and 4 s for PtPd- and PtRh-functionalized WO_3_ NFs, respectively. A similar study by Jang et al.^116^ has also revealed that Pt–Ag NPs exhibit higher catalytic activity than Ag and Pt NPs, respectively. Modification of WO_3_–SnO_2_ NFs with Pt–Ag NPs not only enhanced the response (*R*_a_/*R*_g_ = 2.25) but also the selectivity to 5 ppm of H_2_S. The enhancement in the performance could also be attributed to the hollow morphology of the Pt–Ag NPs, which further promoted the dissociation of H_2_S compared to the interferents. Table 1 summarizes the progress of recently reported pristine and catalyst-loaded SMOs for selective detection of various gases.

**Table 1:** Recently Reported Gas-Selective Sensors Based on Various Pristine and Catalyst-Loaded Semiconducting Metal Oxides

sensing material	synthesis route	response definition	response	target gas	interferents	ambient condition	ref
Pd decorated MnO_2_ film	sputtering	*R*_a_/*R*_g_	11.4 at 100 ppm	H_2_	H_2_S, NH_3_, and CO	air, 100 °C	(121)
Pd modified ZnO NW arrays	electrodeposition	*R*_a_/*R*_g_	13 100 at 100 ppm	H_2_	CO, CH_4_, ethanol, and acetone	air, RT	(122)
WO_3_ nanoplates	hydrothermal	*R*_g_/*R*_a_	131.75 at 100 ppm	NO_2_	CO, CO_2_, NO, acetone, NH_3_, SO_2_, and LPG	air, 100 °C	(123)
rGO–CO_3_O_4_ NFs	electrospinning	(*R*_g_ – *R*_a_)/*R*_g_	53.6% at 50 ppm	NH_3_	methanol, benzene, methylbenzene, formaldehyde, acetone, and ethanol	air, RT	(124)
Pt-SnO_2_ hollow spheres	electrospray etching	*R*_a_/*R*_g_	10.8 at 1 ppm	H_2_S	ethanol, H_2_, toluene, acetone, NH_3_, CO, and pentane	air, 250 °C	(19)
Pt-SnO_2_ NTs	electrospinning etching	*R*_a_/*R*_g_	89.3 at 1 ppm	H_2_S	pentane, NH_3_, CO, toluene, acetone, H_2_, and ethanol	air, 300 °C	(20)
*PI-ZnFe_2_O_4_	molten salt route	*R*_a_/*R*_g_	≈22.5 at 50 ppm	H_2_S	ethanol, acetone, C_4_H_10_O, C_4_H_11_N, and CH_2_O	air, 260 °C	(125)
Pt-SnO_2_ NTs	electrospinning	*R*_a_/*R*_g_	192 at 5 ppm	acetone	H_2_S, toluene, pentane, CO, NO, CH_4_, and H_2_	air, 350 °C	(14)
Pd-WO_3_ NFs	electrospinning	*R*_a_/*R*_g_	43.5 at 5 ppm	H_2_S	acetone, toluene, CO, ethanol, NH_3_, and pentane	air, 250 °C	(126)
RuO_2_-WO_3_ NFs	electrospinning	*R*_a_/*R*_g_	78.61 at 5 ppm	acetone	H_2_S, toluene, ethanol, pentane, ammonia, H_2_, and water vapor	air, 350 °C	(18)
Pt-ZnO NFs	electrospinning	*R*_a_/*R*_g_	13.07 at	acetone	CO, NH_3_, NO, and toluene	air, 450 °C	(25)
Pt-WO_3_ NFs	electrospinning	*R*_a_/*R*_g_	88.04 at 5 ppm	acetone	H_2_S, toluene, ethanol. CO, NH_3_, and CH_4_	air, 350 °C	(127)
Pt-SnO_2_ NFs	electrospinning	*R*_a_/*R*_g_	141.92 at 5 ppm	acetone	ethanol, H_2_S, HCHO, toluene, CO, CH_4_, and NH_3_	air, 350 °C	(15)
Au-ZnO microspheres	hydrothermal	*R*_a_/*R*_g_	311.3–100 ppm	acetylene	CO, CH_4_, acetone, H_2_, HCHO, and C_2_H_4_	air, 183.5 °C	(128)
Au loaded ZnO NPs	coprecipitation	*R*_a_/*R*_g_	92 at 100 ppm	toluene	ethanol and acetone	air, 377 °C	(105)
PtPd-WO_3_ NFs	electrospinning	*R*_a_/*R*_g_	97.5 at 1 ppm	acetone	H_2_S, CH_3_SH, toluene, CO. H_2_, ethanol, and NH_3_	air, 350 °C	(118)
PtRh-WO_3_ NFs	electrospinning	*R*_a_/*R*_g_	104 at 1 ppm	acetone	H_2_S, CH_3_SH, toluene, CO, H_2_, ethanol, and NH_3_	air, 350 °C	(118)
PtNiO-WO_3_ NFs	electrospinning	*R*_a_/*R*_g_	340 at 1 ppm	H_2_S	acetone, CH_3_SH, toluene, CO, H_2_, ethanol, and NH_3_	air, 300 °C	(118)
Pt-WO_3_ microbelts	electrospinning	*R*_a_/*R*_g_	378.12 at 5 ppm	H_2_S	acetone, ethanol, NH_3_, and CO	air, 365 °C	(129)
CuO nanosheets	hydrothermal	*R*_g_/*R*_a_	1.25 at 10 ppb	H_2_S	SO_2_, NO, H_2_, CO, and ethanol	air, RT	(130)
CuO@WO_3_ nanoplates	hydrothermal	*R*_a_/*R*_g_	223 at 5 ppm	H_2_S	H_2_, CH_4_, CO, SO_2_, ethanol, isopropanol, acetone, methanol, methanol, benzene	air, 100 °C	(131)
hierarchical Co-doped ZnO	coprecipitation	*R*_a_/*R*_g_	54 at 50 ppm	ethanol	*n*-butyl alcohol, methanol, acetone, toluene, formaldehyde, cyclohexane	air, 180 °C	(132)
*Co-doped In_2_O_3_ nanorods	hydrothermal	*R*_a_/*R*_g_	23.2 at 10 ppm	HCHO	acetone, ethanol, toluene, NH_3_, and CO	air, 130 °C	(133)
C–N/SnO_2_/ZnO/Au microspheres	electrospinning	*R*_a_/*R*_g_	1970 at 50 ppm	triethyl-amine	ethanol, methanol, acetone, toluene, bwnzene, and NH_3_	air, 280 °C	(134)
Pt–Au In_2_O_3_ nests	hydrothermal	*R*_a_/*R*_g_	40 at 50 ppm	acetone	NH_3_, CO, benzene, toluene, and xylene	air, 160 °C	(135)
Ni doped SnO_2_ NPs	hydrothermal	*R*_a_/*R*_g_	130 at 100 ppm	HCHO	ethanol, methanol, dipropylamine, acetone, NH_3_, CO, and NO	air, 200 °C	(136)
Rh doped SnO_2_ NFs	electrospinning	*R*_a_/*R*_g_	60.6	acetone	ethanol, methanol, HCHO, xylene, toluene, and benzene	air, 200 °C	(137)
ZnO/CuO NTs	ultrasonic spray pyrolysis and chemical bath deposition	(*R*_g_ – *R*_a_)/*R*_a_	25% to 5 ppm	H_2_S	acetone, ethanol, methanol, HCHO, SO_2_, Cl_2_, and O_2_	air, 50 °C	(138)
Co-doped SnO_2_ NFs	electrospinning	*R*_a_/*R*_g_	40.1 at 100 ppm	ethanol	acetone, methanol, HCHO, toluene, benzene, and xylene	air, 300 °C	(139)
Ru-doped In_2_O_3_ nanosheets	hydrothermal	*R*_a_/*R*_g_	128.9 at 100 ppm	xylene	acetone, toluene, ethanol, benzene, CO, and methane	air, 120 °C	(140)
CuO_*x*_/Co_3_O_4_	flame-spray pyrolysis	*R*_a_/*R*_g_	5.2 at 1 ppm	formaldehyde	acetone, toluene, ethanol, NO, acetaldehyde, NH_3_, CO, CH_4_	air, 75 °C	(141)
Y-doped ZnO	flame-spray pyrolysis	*R*_a_/*R*_g_	155 at 1 ppm	acetic acid	isoprene, ethanol, acetone, H_2_	air, 350 °C	(142)
Si-doped MoO_3_	flame-spray pyrolysis	*R*_a_/*R*_g_	1.53 at 1 ppm	ammonia	acetone, NO, CO	air, 400 °C	(143)
Ti-doped ZnO	flame-spray pyrolysis	*R*_a_/*R*_g_	5.4 at 500 ppb	isoprene	acetone, ethanol, ammonia	air, 325 °C	(144)

#### 3.2.2 Noble Metal Single-Atom Catalysts

In terms of catalyst atom efficiency, noble metal NPs have brought about an enormous enhancement compared to bulk or film type catalysts. The enhancement of the gas sensing performance in these systems increases with decreasing NP diameter due to the exposure of more catalyst atoms toward target gas species and oxygen molecules in air, as a consequence of an increased surface area-to-volume ratio (SA/V). Theoretically, the ultimate maximization of catalyst atom efficiency can be achieved if every single catalyst atom can take part in the reaction, i.e., if every catalyst atom is exposed on the surface of the support material without agglomeration. Note that reaction pathways on single catalyst atoms may be different to atom assemblies of the same element, offering new opportunities for selectivity optimization.^145^ This theoretical catalyst design, in fact, has been successfully achieved using several different approaches, and proven to be highly effective in many research fields.^146−148^

Such design, known as “single-atom catalysts (SACs)”, is typically composed of atomically isolated catalyst atoms stabilized on the surface of the support material, hence, the form is clearly dissimilar to doping foreign atoms into the support material body. Stabilization of single-atoms (SAs) on metal oxides relies on surface defect sites (such as cationic and anionic point defect,^149^ or step edges),^150^ and the rational design strategy for higher loading of the SAs without causing agglomeration is essential for increasing the specific surface area of the support metal oxide by nanostructuring.

**Figure 4:**
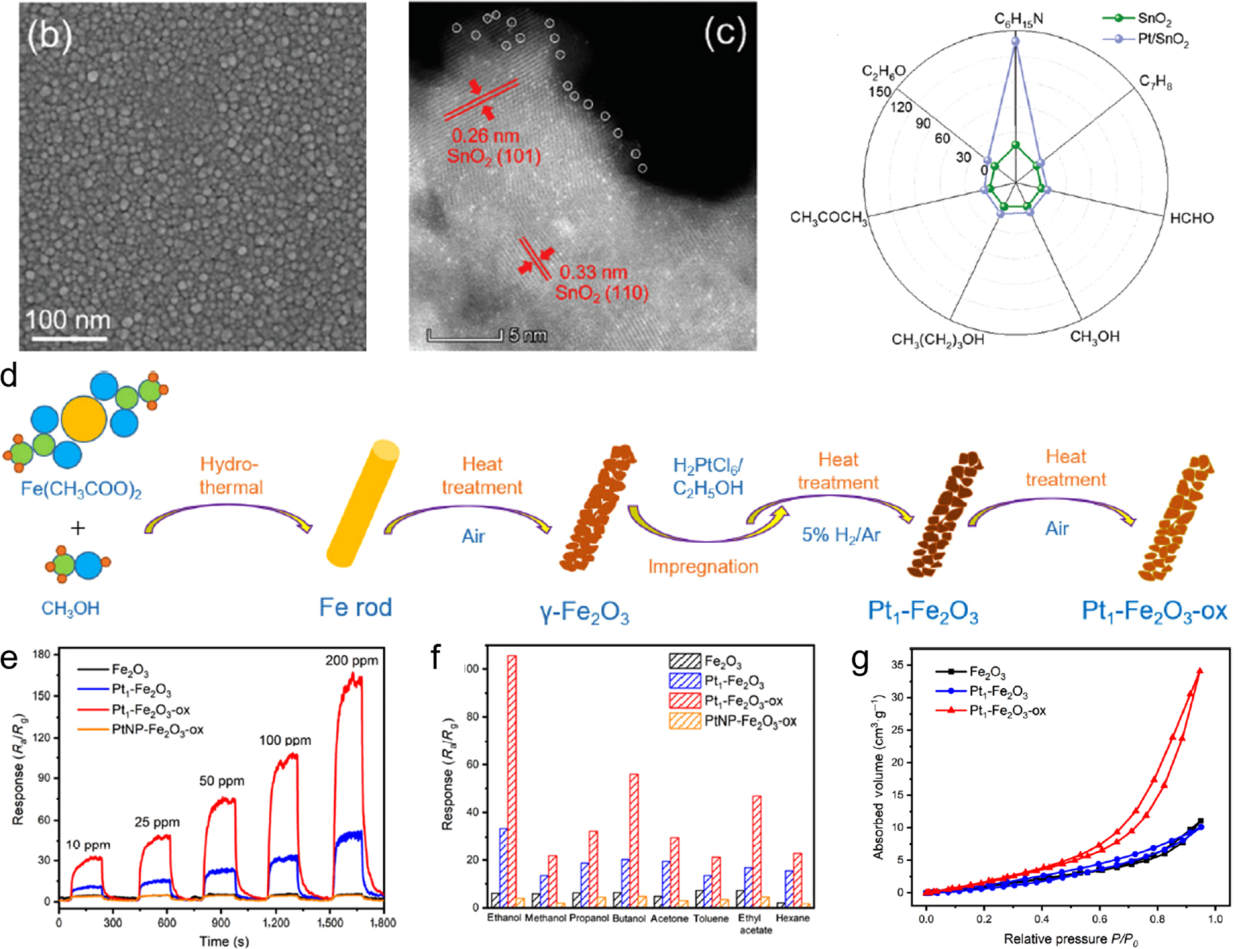
(a) SEM image of a SnO_2_ thin film, (b) HAADF-STEM image of single-atom Pt/SnO_2_ thin films, (c) selectivity of SnO_2_ and Pt/SnO_2_ thin films sensors to various 10 ppm gases toward TEA. Reproduced with permission from ref (152). Copyright 2020 Royal Society of Chemistry. (d) Schematic illustration of the synthesize procedures of Fe_2_O_3_, Pt_1_–Fe_2_O_3_, and Pt_1_–Fe_2_O_3_-ox, (e) response of the Fe_2_O_3_, Pt_1_–Fe_2_O_3_, and Pt_1_–Fe_2_O_3_-ox to C_2_H_5_OH in concentrations of 10–200 ppm at 280 °C, (f) selectivity to various gases (100 ppm) of the Fe_2_O_3_, Pt_1_–Fe_2_O_3_, and Pt_1_–Fe_2_O_3_-ox, (g) comparative analysis of the C_2_H_5_OH adsorption–desorption isotherms to the Fe_2_O_3_, Pt_1_–Fe_2_O_3_, and Pt_1_–Fe_2_O_3_-ox. Reproduced with permission from ref (154). Copyright 2021 Springer Nature.

Since the first emergence in 2011,^151^ there have been several successful attempts to employ metal oxide support-based SACs in chemiresistive gas sensing. In 2020, Xu et al. fabricated Pt SAs-functionalized SnO_2_ ultrathin films (Pt/SnO_2_) via atomic layer deposition (ALD) technique.^152^ After careful control of the thin film thickness for response optimization (Figure 4a), Pt SAs were deposited onto SnO_2_ for further improvement of the sensor response and detection limit (Figure 4b). The main mechanism for the enhancement of the gas sensor response is the chemical sensitization mechanism; the X-ray photoelectron spectroscopy (XPS) results revealed that Pt/SnO_2_ films possess far higher surface-adsorbed oxygen species concentration (64.4%) compared to those of pristine SnO_2_ and annealed (PtO-functionalized) SnO_2_ films. Pt/SnO_2_ showed substantial increments in response toward all measured gas species, but with exceptionally high enhancement for triethylamine (TEA) (Figure 4c). Although the explanation for the selective catalyzed reaction between Pt/SnO_2_ with TEA is vague, it can be suggested that the Pt SAs stabilized on the surface of SnO_2_ films may catalyze the reaction between SnO_2_ and TEA in an unexpected direction, such as serving as favorable adsorption sites for the TEA molecules over other interferent gas species. It has already been suggested that surface-stabilized high-valent Pt species are known to catalyze the reaction between the metal oxide supports and gas species in a way that is distinctive from those with Pt nanoparticles or Pt dopants.^153^

Li et al. further investigated not only on the relationship between the coordination environment of Pt atomic sites and the gas sensing performance but also the adsorption capability of Pt SAs toward certain gas species.^154^ As displayed in Figure 4d, after impregnation of Pt precursor onto γ-Fe_2_O_3_ nanorods, the mixture was first heat-treated in H_2_ (reducing) atmosphere and followed by oxidative heat treatment (Pt_1_–Fe_2_O_3_-_OX_). Through the oxidative heat treatment, the Pt species exhibited higher valence state than preoxidative heat treatment sample, and hence, a different coordination environment. Pt_1_–Fe_2_O_3_-_OX_ showed dramatic selective response enhancement toward ethanol in contrast to the inferior improvement of Pt_1_–Fe_2_O_3_ (Figure 4e,f), which is attributed to selective accommodation of ethanol molecule absorption onto the high-valence state Pt species, as was the case in the study of Xu et al. In the ethanol gas adsorption–desorption isotherms (Figure 4g) performed by Li et al., Pt_1_–Fe_2_O_3_-_OX_ showed 3-fold increase (34.05 cm^3^ g^–1^) in the adsorbed volume of ethanol compared to those of Pt_1_–Fe_2_O_3_ (10.11 cm^3^ g^–1^) and Fe_2_O_3_ (11.09 cm^3^ g^–1^), which suggests that only the Pt SAs with high-valence state can effectively provide high adsorption capacity for ethanol.

**Figure 5:**
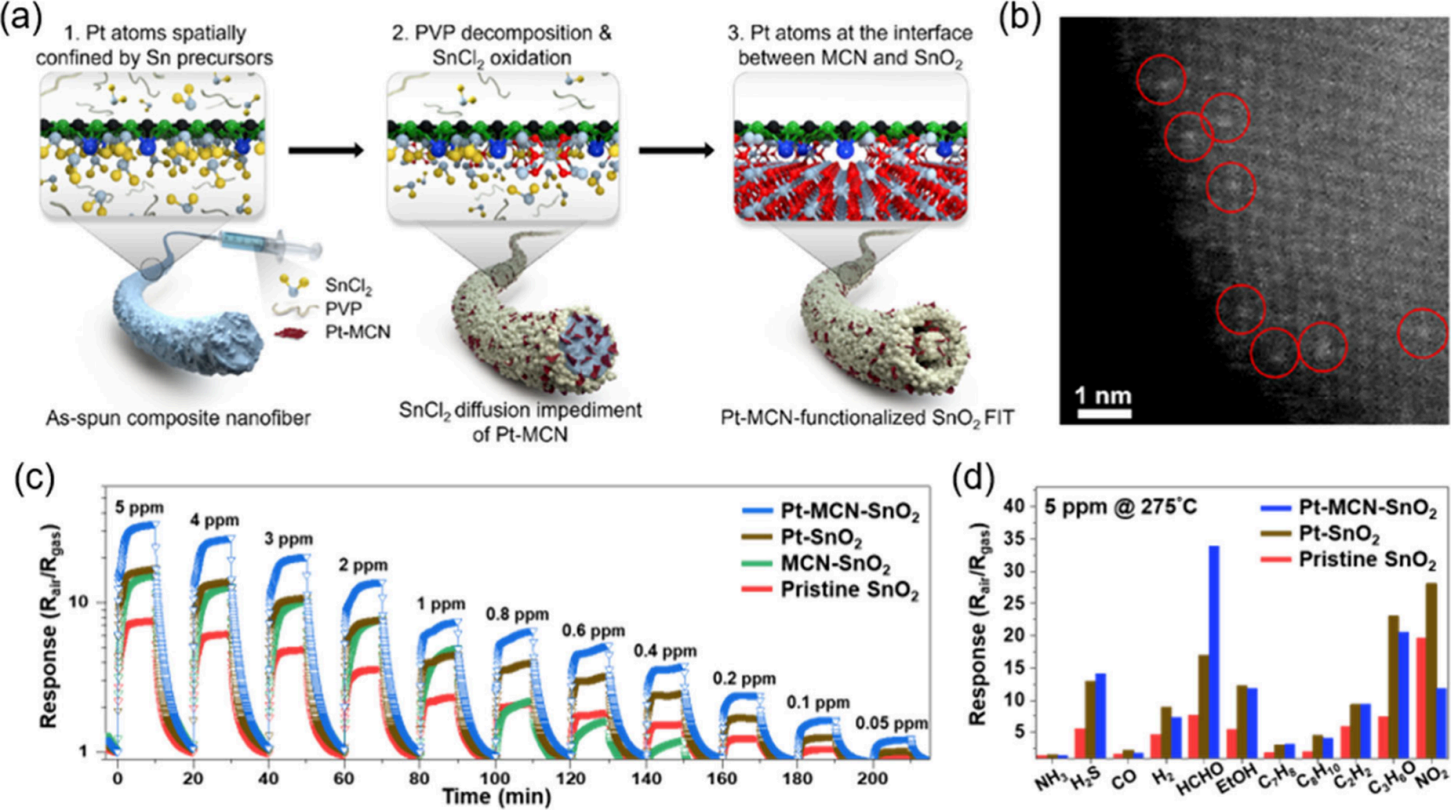
(a) Schematic illustration of the Pt SA delivery process and nanofiber-in-tube (FIT) structure formation, (b) magnified HAADF-STEM image of Pt–MCN-SnO_2_, showing Pt single-atoms on SnO_2_ nanograin, (c) formaldehyde gas sensing results of Pt–MCN-SnO_2_ compared with reference samples, (d) selectivity of Pt–MCN-SnO_2_ toward nine different gas species compared with reference samples. Reproduced with permission from ref (155). Copyright 2020 American Chemical Society.

Pt SACs on metal oxide supports for chemiresistive gas sensing can also be situated in a unique coordination environment. Shin et al. stabilized Pt SAs at the heterojunctions in a one-dimensional nanocomposite, such that the Pt SAs are coordinated by both shredded carbon nitride nanosheets and SnO_2_ nanograins (Pt–MCN-SnO_2_), i.e., the coordination environment of Pt consists of Pt–O, Pt–Sn, and Pt–N/C (Figure 5a,b).^155^ With the Pt SAs and SnO_2_ combination, incorporation of carbon nitride completely alters the gas sensor selectivity; formaldehyde, which Pt SAs-stabilized SnO_2_ thin films had a very weak response to, was found to interact most actively with Pt–MCN-SnO_2_ (Figure 5c,d).

**Figure 6:**
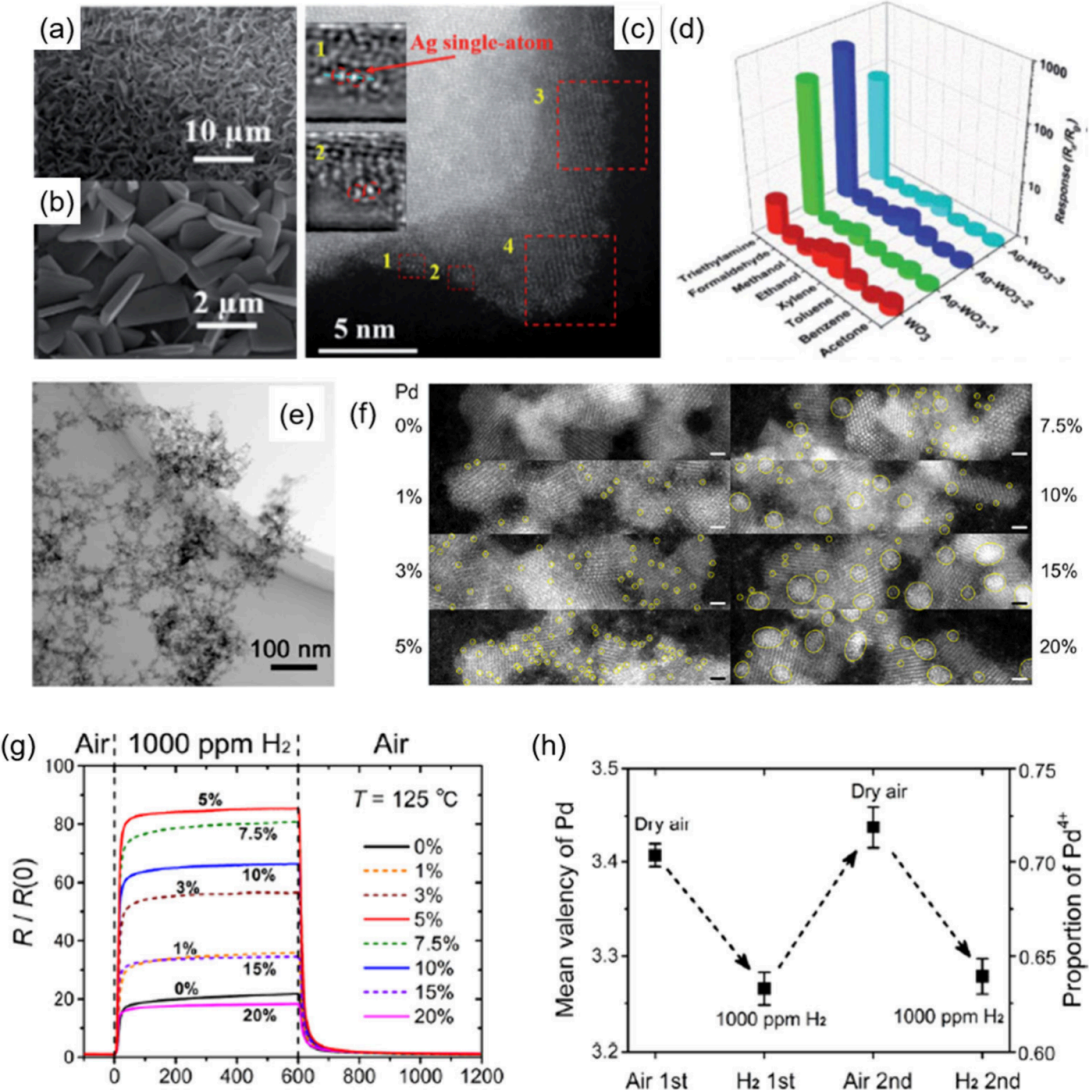
(a,b) FESEM images of Ag single-atoms-loaded WO_3_ nanoplates, (c) HAADF-STEM image of Ag single-atoms-loaded WO_3_ nanoplates, (d) selectivity of Ag-WO_3_ and pure WO_3_ (the concentration of TEA is 10 ppm at 175 °C and that of other gases is 50 ppm at 250 °C). Reproduced with permission from ref (156). Copyright 2021 Royal Society of Chemistry. (e) BF-STEM image of as-grown Co_3_O_4_ NP aggregates with a web-like morphology, (f) HAADF-ac-STEM images of Co_3_O_4_ and Pd–Co_3_O_4_ NPs with different Pd contents. The small and larger yellow circles denote single Pd atoms and PdO_*x*_ clusters, respectively. Each specimen was sampled directly from the corresponding NP film after heat treatment at 200 °C in a dry air flow. Scale bar: 1 nm, (g) response and recovery sensitivity curves acquired at 125 °C for 1000 ppm of H_2_, (h) mean Pd valence states and the Pd^4+^ proportions of the Pd5%–Co_3_O_4_ NP film at 125 °C as a function of the gas exposure sequence. Reproduced with permission from ref (157). Copyright 2020 American Chemical Society.

Not only the metal oxide support or the coordination environment for the SACs can be modified, but also the element for the SACs can be replaced with other noble metals. Zeng et al. employed atomically deposited Ag on WO_3_ nanoplates (AD-Ag-WO_3_) (Figure 6a–c) with different loading amounts of Ag SAs on WO_3_ (AD-Ag-WO_3_-1, AD-Ag-WO_3_-2, and AD-Ag-WO_3_-3).^156^ Regardless of the Ag SAs loadings, functionalization of WO_3_ with Ag SAs resulted in highly selective TEA gas sensors (Figure 6d). In another case, Koga prepared homogeneously dispersed, and mixed Pd^4+^ and Pd^2+^ states of Pd SA on aggregates of Co_3_O_4_ nanoparticles for highly selective hydrogen gas sensor (Figure 6e,f).^157^ To study the role of Pd SA additives in Co_3_O_4_ for the selective sensing of hydrogen, *in situ* XPS and Pd K-edge X-ray absorption spectroscopy were conducted to demonstrate the partial (∼10%) reduction of metastable Pd^4+^ state to Pd^2+^ upon exposure to H_2_ (Figure 6g,h). This reversible catalytic redox reaction (Pd^4+^ ↔ Pd^2+^) allows overcoordinated oxygen species on Pd^4+^ to selectively react with the H_2_ molecules, hence, accelerating the water formation reaction.

From the above studies, it can be concluded that the selectivity of SACs-functionalized metal oxides can be adjusted by (1) changing the support metal oxide (2) modifying the coordination environment of the SAs, or (3) employing SAs of different noble metals. Unlike the conventional chemical and electronic sensitizations reported on the NPs-based catalysis, the SACs offer a new pathway for sensitizations of chemiresistive gas sensors by providing selective adsorption sites for desired gas species. Chemiresistive gas sensing with SACs is a relatively new research topic to the field and currently there has not been many reports. There still remain numerous possible assemblies of nanostructures, metal oxides, and SACs to be explored, and we anticipate that soon there will be a full library of SACs-based gas sensors targeted for gas species desired for detection.

#### 3.2.3 Future Prospects in Metal Oxide Functionalization with Catalysts

The primary goals in using noble metal catalysts for chemiresistive gas sensing is increasing the catalytic efficiencies, which has been partially accomplished by the utilization of NPs, and further with the SACs. Due to the nature of high-valent state of the noble metal SACs on metal oxide supports, their gas sensing sensitivity and selectivity enhancement mechanisms are sharply distinctive from those of NPs. Especially, the main chemical sensitization effect, the spillover of oxygen molecules on the noble-metal NPs, would be diminished in the metal oxide gas sensors functionalized with high oxidation state SACs, and thus, relatively poor response value enhancement toward target gases is rather apparent compared to those functionalized with the NPs. Therefore, the improvement of selectivity and/or catalyst atom efficiency was allegedly at the expense of the response value.

Forming heterojunctions while simultaneously functionalizing with the SACs, as suggested by Shin et al., can be one solution to tackle the challenge.^155^ In addition to the catalytic effect of the Pt SACs, carbon nitride, which was the support material for the Pt SACs before incorporation onto the electrospun SnO_2_ nanofiber-in-tubes, created multiple heterointerfaces with SnO_2_. Creation of the heterointerfaces caused transfer of charge carriers between carbon nitride and SnO_2_, forming electron accumulation layers on SnO_2_ that effectively attract and adsorb a higher concentration of oxygen species. This behavior is similar to that of the spillover effect of Pt NPs, endowed Pt-MCN-SnO_2_ with extremely high response toward formaldehyde, comparable to those metal oxides functionalized with NP catalysts. Metal ensemble catalysts, in fact, are among excellent candidates capable of exhibiting the same phenomenon, in that the SACs on metal oxide nanoparticle supports of the ensemble catalysts can act as the bifunctional catalyst with substantially enhanced selectivity and response.^158,159^

Low-nuclearity metal catalysts can be another ideal solution to optimizing this trade-off.^160,161^ Low-nuclearity metal catalysts are ultrasmall clusters of metal atoms, commonly referring to metal dimers (two-atoms cluster) and trimers (three-atoms cluster) that are precisely controlled and stabilized on the support material surface. These dimers and trimers obviously exhibit distinct properties from that of the NPs, and even from the SACs. The different behaviors of the low-nuclearity metal catalysts from the SACs, presumably, have originated from higher concentration of charge localization induced by addition of metal atoms (i.e., more “metallic” nature than the SACs), and this higher nuclearity is reported to even enhance the durability of the catalysts.^162^ We predict that when the low-nuclearity metal catalysts are employed for chemiresistive gas sensing, they will maintain the benefits of the high-valent metal catalysts, which is providing favorable adsorption sites to desired gas species, as well as improved spillover effect from the more metallic characteristics with the higher charge concentration.

### 3.3 Defect/Phase Engineering in Metal Oxides

#### 3.3.1 Defect Creation and Phase Control

The adsorption of gas molecules on SMO surfaces is a critical aspect in resistive-type sensory devices. Practical evidence proves that defect engineering in SMOs plays a critical role in tuning the electronic structure and electrical conductivity of SMOs, as well as increasing the number of active sites for chemical reactions, altogether leading to enhanced gas absorption characteristics. Specifically, several reports have evidenced how defect engineering improves the sensitivity and selectivity of SMOs toward target gases (Table 2 and references therein). Prominent methods such as chemical reduction, heat treatment, ultraviolet reduction, plasma etching, vacuum activation, and ion beam irradiation can introduce structural defects in SMOs, reducing the coordination number of the surface atoms.^149,150,163,164^ Also nonequilibrium NP synthesis routes that feature steep temperature (e.g., flame spray pyrolysis) gradients yield high defect densities.^165^ The type and quantity of defects can be controlled by the material’s degree of structural purity, growth rate and conditions, and preparation techniques.

**Table 2:** Recently Reported Selectivity of Semiconducting Metal Oxides Based on Defect Engineering Technique

sensing material	defect formation technique	response definition	response/conc (ppm)	target gas	interferents	ambient condition	ref
SnO_2_ NWs	ion-beam irradiation	*R*_g_/*R*_a_	14.24–2 ppm	NO_2_	SO_2_, NH_3_, H_2_, acetone, and ethanol	air, 25 °C	(166)
SnO_2_ NPs	calcination in air	*R*_a_/*R*_g_	103–100 ppm	ethanol	acetone, CO, NH_3_, methanol, toluene, and HCHO	air, 175 °C	(167)
SnO_2_ NPs	calcination in helium	*R*_a_/*R*_g_	20.5–100 ppm	HCHO	acetone, ethanol, CO, NH_3_, methanol, and toluene	air, 200 °C	(167)
SnO_2_ NPs	calcination in oxygen	*R*_a_/*R*_g_	14.8–100 ppm	acetone	ethanol, CO, NH_3_, methanol, toluene, and HCHO	air, 200 °C	(167)
ITO NTs	doping	*R*_a_/(*m*·*R*_g_)^a^	24.5–100 ppm	HCHO	ethanol, acetone, methanol, and CO	helium, 160 °C	(168)
Fe–C codoped WO_3_ microspheres	doping	*R*_a_/*R*_g_	7.3–0.9 ppm	acetone	NO, NH_3_, toluene, methanol, ethanol, and CO	air, 300 °C	(169)
Cu-doped WO_3_ NFs	doping, phase structure design	*R*_a_/*R*_g_	6.43–20 ppm	acetone	*n*-butanol, ethanol, toluene, methanol, isopropyl alcohol, methanal, NH_3_, CO_2_, ethylene glycol	air, 300 °C	(170)
mesoporous Zr-doped In_2_O_3_	doping	*R*_g_/*R*_a_	65–0.5 ppm	NO_2_	H_2_S, NH_3_, ethanol, acetone, SO_2_, Cl_2_, and O_3_	air, 75 °C	(171)
SnO_2_ pellets	quenching	*R*_a_/*R*_g_	24.9–100 ppm	ethanol	chloroform, HCHO, methanol, acetone, and NH_3_	air, 300 °C	(172)
Gd-doped WO_3_/RGO	doping	*R*_a_/*R*_g_	54–50 ppm	acetone	NH_3_, H_2_, ethanol, and LPG	air, 200 °C	(173)
Al-doped ZnO	nonequilibrium synthesis	*R*_a_/*R*_g_	11–1 ppm	acetone	NH_3_, CO and isoprene	air, 450 °C	(174)

a m is the mass of the coated sensing materials.

**Figure 7:**
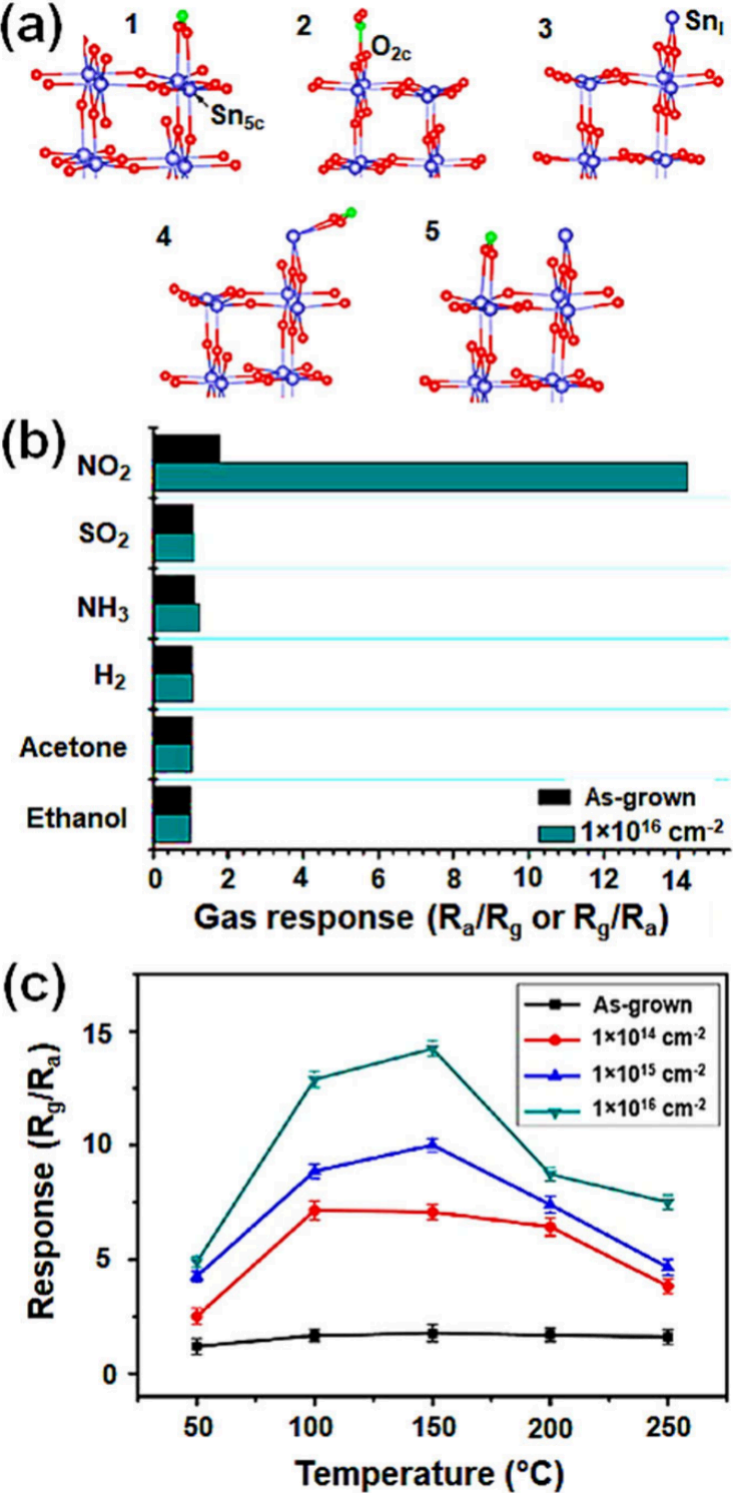
(a) Adsorption geometries for NO_2_ on a perfect SnO_2_ surface [1, 2], surface tin interstitial [3], and NO_2_ on a defective SnO_2_ surface [4, 5]. The blue, red, and green balls indicate tin, oxygen, and nitrogen atoms, respectively. Sn_5c_, O_2c_, and Sn_I_ indicate 5-coordinate tin atom, 2-coordinate O atom, and surface interstitial tin atom, respectively. (b) Sensor response values of unirradiated and irradiated SnO_2_ nanowires (fluence of 1 × 10^16^ ions/cm^2^) to 2 ppm of various gases at 150 °C, and (c) variation of the sensor responses of unirradiated SnO_2_ nanowires and irradiated SnO_2_ nanowires in the range of 25–250 °C. Fluences of 1 × 10^14^, 1 × 10^15^, and 1 × 10^16^ ions/cm^2^ were irradiated on SnO_2_ nanowires. Reproduced with permission from ref (166). Copyright 2016 American Chemical Society.

Atomic-scale defects can be simply created using ion-beam irradiation, which involves the penetration and subsequent collision of high-energy ions or electrons with the nuclei of SMOs.^175,176^ Kwon et al.^166^ irradiated SnO_2_ nanowires with helium (He) ions to form structural defects and tin (Sn) interstitials by reducing the Sn^4+^ species to Sn^2+^ (Figure 7a). In a perfect SnO_2_ (Figure 7a, schematics 1–2), the unsaturated five-coordinate Sn atoms and the two-coordinate O atoms would be the adsorption sites for NO_2_. However, for a defective SnO_2_, active adsorption sites for NO_2_ would be the five-coordinate surface Sn atoms and the interstitial Sn atoms, which possess a higher adsorption energy for NO_2_ compared to the case of the perfect SnO_2_ surface. This mechanism favored a rapid and selective detection of NO_2_ against SO_2_, NH_3_, H_2_, acetone, and ethanol at 150 °C (Figure 7b). Experimental observations revealed that the response to NO_2_ increased with the flux of He, which is corroborated by the fact that the dosage of He ions varied proportionally with the increase of defects in SnO_2_ (Figure 7c).

Different calcination atmospheres can also introduce differing numbers and types of defects in SMOs.^177−179^ Liu et al. tailored the defects in SnO_2_ NPs by calcining them separately in air, helium, or O_2_ atmosphere, and investigated the gas-sensing selectivity of the defects-induced SnO_2_ NPs.^167^ The investigated NPs had the same size and effectively identical in their morphology. Surprisingly, the tetragonal SnO_2_ calcined in air, helium, and O_2_ showed different selectivity properties toward ethanol (*R*_a_/*R*_g_ = 103 at 175 °C), formaldehyde (*R*_a_/*R*_g_ = 20.5 at 200 °C), and acetone (*R*_a_/*R*_g_ = 14.3 at 200 °C) upon exposure to 100 ppm of various gases. Unlike the defect-free oxygen-calcined SnO_2_ NPs that showed the selectivity toward the detection of acetone, the air- and the He-calcined SnO_2_ NPs each exhibited single oxygen vacancies and triple vacancy associates, respectively, that favored selective sensing of ethanol and formaldehyde, respectively as well. The different band structures induced by the defects could lead to higher ionic potentials in SnO_2_, and improved selectivity because the adsorption of a gas is facilitated when its reduction potential matches the band energy of the sensing material. In addition, stronger Brønsted acidic gases such as formaldehyde (p*K*_a_ = 19.3) can remove the strongly chemisorbed oxygen species around the defects that induce the ionic potential change, promoting a further enhancement in the selectivity.

**Figure 8:**
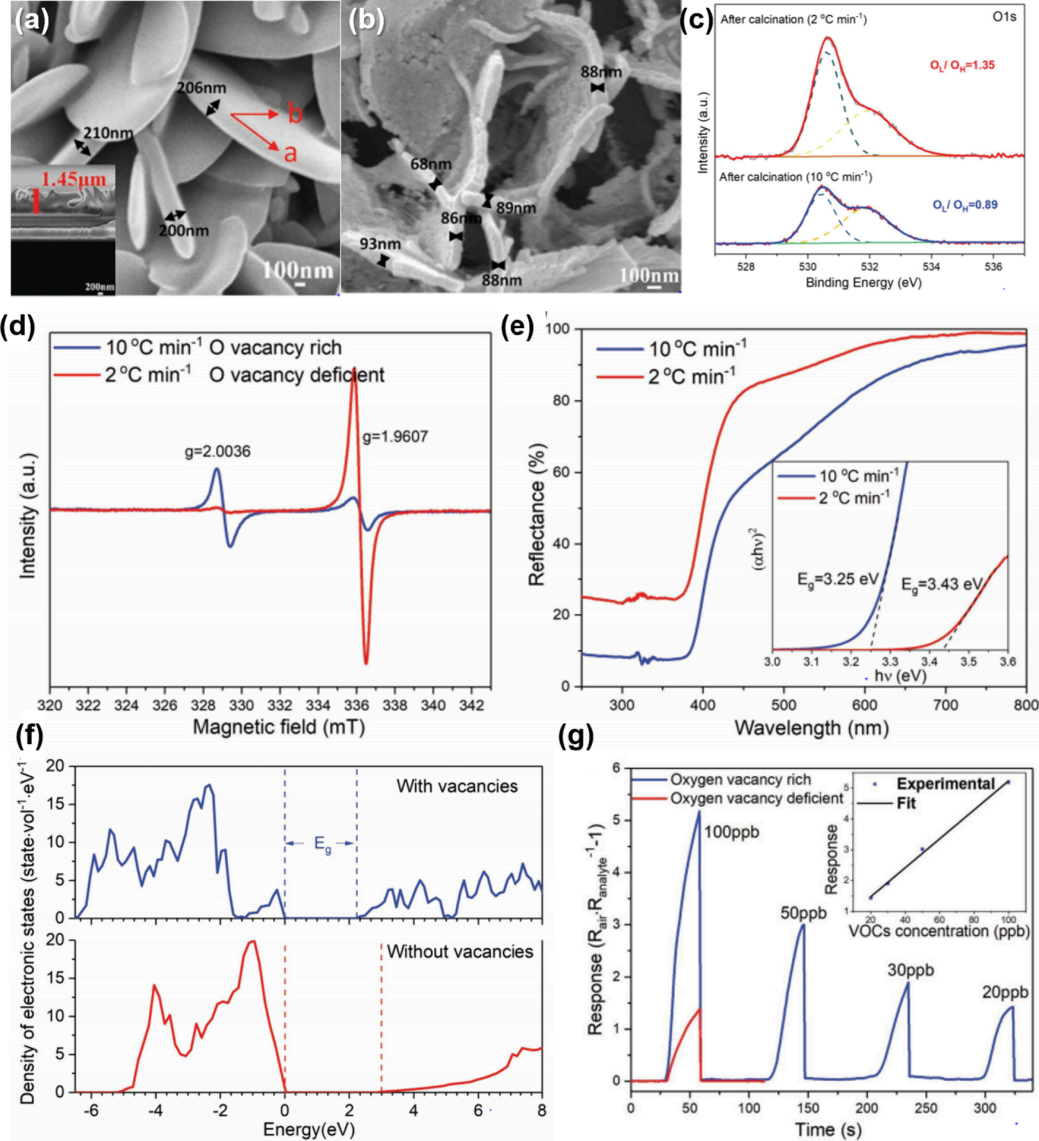
SEM images of ZIF-L sheets (a) before and (b) after calcination in air. (c) XPS analysis in the vicinity of O 1s peak, (d) EPR spectra, and (e) UV–vis reflectance spectra of the samples under different heating rates (2 °C min^–1^ and 10 °C min^–1^, respectively). (f) Calculated density of electronic states (DOS) for ZnO with and without oxygen vacancies. (g) Gas sensing results toward 100–20 ppb CO gas analytes using oxygen vacancy rich and deficient sensors. Reproduced with permission from ref (181). Copyright 2019 Wiley-VCH.

Differently from Liu and coauthors, Zhou et al. achieved selective formaldehyde sensing by tuning the oxygen vacancies in In_2_O_3_/ITO (indium tin oxide) nanotubes by controlling the doping concentration of the Sn precursor.^168^ During calcination in air, the precursors oxidized to form SnO_2_ and In_2_O_3_, which then inter-reacted to form ITO. Specifically, Sn^4+^ ions diffused into In_2_O_3_ to replace In^3+^ ions, creating positive charges therein. The positive charge was compensated with surrounding interstitial oxygen anions, generating oxygen vacancies with its concentration varying linearly with the amount of Sn^4+^ doping. As a result, enhanced responses were observed for up to 7 mol % of Sn doping (*R*_a_/*R*_g_ = 24.5 toward 100 ppm formaldehyde at 160 °C), and are attributed to the large number of oxygen vacancies that provide preferential adsorption sites for target gas analytes.^180^ Control of the ramping rate during calcination is another method to tune the density of the oxygen vacancy defects in oxides. In this regard, Yuan et al. reported a simple approach to generate adjustable oxygen vacancy defects in ZIF-L (zeolitic imidazolate framework-layered) sheets during the calcination step, through which the electronic structure of the ZnO-based sensing materials can be finely tuned to exhibit high sensing performances. ^181181^ The ZIF-L materials exhibit layered structure (200 nm of thickness) which turned into ZnO nanosheets with a sub-100 nm thickness after calcination at 400 °C in air (Figure 8a,b). Herein, the calcination ramping rate was controlled at 2 and 10 °C min^–1^, respectively, to tune the density of oxygen vacancy defects in ZIF-L-derived ZnO nanosheets. The qualitative and quantitative analyses of oxygen vacancy defects were investigated via various analysis tools including XPS, electron paramagnetic resonance (EPR) spectroscopy, and UV–visible reflectance. The O 1*s* spectra in XPS analysis showed that the peak related to the lattice oxygen dramatically decreased for the sample calcined at 10 °C min^–1^, demonstrating the formation of oxygen vacancy defects (Figure 8c). In addition, EPR spectroscopic measurements exhibit a distinct EPR signal at *g* = 1.9607 for the sample calcined at 10 °C min^–1^, which is related to the shallow donor impurities, oxygen vacancy defects in ZnO in this system (Figure 8d). Note that the magnetic dipoles of unpaired electrons interact under a specific applied magnetic field, showing evidence of intrinsic vacancy or interstitial defects in oxides. Based on the abundant shallow level defects, reduction of the optical bandgap was proven by UV–visible reflectance spectra and calculated density of electronic states (Figure 8e,f). As a result, ZnO sensor with abundant oxygen vacancy defects exhibited highly sensitive and selective CO gas sensing properties (Figure 8g).

High defect concentrations can be also yielded by nonequilibrium synthesis routes like flame-spray pyrolysis. Thereby, sensing NPs are formed by gas–solid conversion through nucleation, coagulation, sintering, aggregation, and agglomeration.^165^ Due to the steep temperature gradients, the high defect densities of elevated temperature are preserved in the product NPs. For instance, Al-doped ZnO prepared by flame spray pyrolysis featured five times higher response compared to hydrothermally made (*R*_a_/*R*_g_ = 246 vs 57–10 ppm acetone at 450 °C, respectively).^174^ This was associated with high oxygen vacancy density of flame-made NPs, as identified by X-ray photoelectron spectroscopy. Such sensors based on fame-made NPs drop-coated onto interdigitated electrodes detected acetone down to 100 ppb with reasonable selectivity (i.e., response ratio >3) over NH_3_, CO, and isoprene.

Besides defect generation, phase control is another key strategy to realize stable sensitivity and selectivity in SMOs. As a well-known oxide, WO_3_ can be engineered into multiple crystalline phases and properties.^182^ Building upon this concept, Shen and co-workers utilized Fe–C codoped ε-phase WO_3_ to attain a stable response (*R*_a_/*R*_g_ = 7.2 toward 0.9 ppm at 300 °C), and a good selectivity toward acetone against several other gases (*R*_a_/*R*_g_ < 1.8).^169^ The ε-phase WO_3_ had been stabilized before also by Cr-^183^ and Si-doping,^184^ and the selectivity was attributed to the ε-phase WO_3_ acentric crystal structure, which possesses a spontaneous dipole moment that strongly attracts polar acetone molecules and therefore accelerates oxidizing reaction of acetone. This implies that maintaining thermal stability during the sensor operation to avoid disrupting the ε-WO_3_ phase is critical to retain the selective sensing property. Doping with Fe was intended to fulfill two purposes; first, to create oxygen vacancies by replacement of W^6+^ with Fe^3+^ ions to form iron–tungsten solid solution for higher response, and second, to contribute to the thermal stability of the ε-WO_3_ phase for maintained selectivity. Similar observations have been reported by Bai et al.^170^ and Kaur et al.,^173^ who observed remarkable selectivity and enhanced response toward acetone upon doping the ε-WO_3_ with copper (Cu) and gadolinium (Gd), respectively. Such ε-WO_3_ have been applied successfully for breath acetone detection in healthy and type-1 diabetes^185^ volunteers during ketogenic dieting^186^ and physical activity,^187^ showing strong correlations to mass spectrometry and blood assay.

#### 3.3.2 Future Prospects in Defect/Phase Control in Metal Oxides

Intrinsic and extrinsic defects in SMOs significantly influence their gas sensing performance as chemiresistors by modulating electronic properties, gas adsorption capacity, and stability. The distribution and stability of oxygen vacancies, determined by synthesis and operating conditions, play a significant role in the sensor’s long-term stability and durability. Oxygen vacancies, the most common intrinsic defect, play a pivotal role in gas sensing by acting as active sites for gas adsorption, facilitating charge transfer, and altering the conductivity of the material.^188,189^ Surface oxygen vacancies primarily enhance adsorption and reaction pathways with target gases, influencing selectivity and sensitivity, while bulk oxygen vacancies affect electronic conductivity and structural stability. However, excessive bulk oxygen vacancies can compromise the stability of SMO crystal structures. At high operating temperatures, oxygen in-diffusion may partially annihilate these defects.^190^

Polymorphism in SMOs further impacts sensing performance by altering defect concentrations, electronic properties, and surface energetics. For instance, the anatase and rutile phases of TiO_2_ exhibit distinct band gaps and stability characteristics, with mixed-phase compositions demonstrating synergistic effects that enhance gas sensing performance by improving charge separation and reaction kinetics.^191^ The incorporation of heteroatom dopants introduces extrinsic defects, such as oxygen or metal vacancies, which can generate additional charge carriers to improve chemisorption and gas sensitivity.^192,193^

For the case of defect-based enhancement techniques, the sensor selectivity still faces prominent challenges despite key achievements. There is limited knowledge regarding the correlation between various types of defects and the related potential reaction mechanisms,^167^ sensor selectivity,^168^ as well as the role of codoping in sensing enhancements.^169^ In order to gain a full understanding of the role of the defects and the different phases of a material toward selective detection of target gas species, a serious amount of studies must be executed to find the general trends in the type of defect, and the gas selectivity it endows. A detailed understanding of the interplay between defect engineering and polymorphism, supported by advanced characterization techniques, is essential for optimizing the design and functionality of SMO-based gas sensors. For instance, insights may be obtained by *in situ* spectroscopic investigations that are surface sensitive and can identify dynamically (1) adsorbed surface species, (2) the type of bonding to vacancies, and (3) analyte interactions. The ultimate aim of the study points toward creating an extensive library of the defect and phase control of metal oxides that are readily available for desired applications.

### 3.4 Integration of Semiconducting Metal Oxides with Gas Separation Filters

Introduction of gas separation filters is a powerful and effective strategy to endow gas sensors with improved selectivity.^194^ By placing the filters either in front of sensors in the form of packed beds or directly on top of sensing materials as overlayers could tune the composition and concentration of gas analytes before reaching the sensing layers. Gas filters are generally classified into physical,^195^ catalytic,^196^ and sorption types.^197^ Physical filters, also known as size-selective filters, selectively allow small target analytes to permeate through while physically discriminating unwanted gases with larger kinetic diameters. On the other hand, chemical filters involve the chemical transformation of unwanted gases into less- or nonreactive forms, allowing only the target gas to reach the detecting layer. Meanwhile, sorption filters enhance the selectivity of the underlying sensing layers by utilizing selective adsorption properties of specific gas molecules based on molecular weight, size of target analytes, polarity, and surface functionality of sorption filters. There have been several attempts to utilize physical, chemical, and sorption filtration media together with conventional sensor materials such as SMOs to attain enhanced selectivity. Filters serve as a first line of defense against unwanted gases. Many commercially available sensors, such as those produced by Figaro, incorporate integrated filters to enhance performance.^198,199^ These examples underscore the practical importance of filter-based solutions in achieving reliable gas sensing under real-world conditions. In this section, we aim to elucidate the filtration strategies, materials, and filtration performance of these filters when integrated with SMO-based gas sensing layers.

#### 3.4.1 Physical Gas Filtration

Physical filters based on zeolites,^200^ covalent organic frameworks (COFs),^201^ metal organic frameworks (MOFs),^202,203^ MOFs/graphene oxide (GO),^204^ perforated graphene,^205^ and carbon^206^ have been commonly employed for the separation of gas mixtures. Typically, the permselectivity depends on the thickness of the filter membrane, the size of pores on the membrane, and the kinetic diameters of gases. Typical kinetic diameters of common gases (shown in parentheses) are as follows: H_2_ (0.289 nm),^202,204,207^ CH_4_ (0.380 nm),^204^ formaldehyde (0.243),^208^ ethanol (0.450 nm), acetone (0.460 nm), toluene (0.585 nm), benzene (0.585),^209^ CO_2_ (0.330 nm),^205,210^ water vapor (0.260 nm),^204^ N_2_ (0.364 nm),^210,211^ propane (0.430 nm),^211^ isoprene (0.550 nm), and NH_3_ (0.330 nm).^212^ Thinner membranes exhibit high permeance, which scales exponentially with the kinetic diameters of gas species.^202,204^ The deposition or growth of these filter membranes on SMOs should be fundamentally effective for the selective infiltration of desirable gas molecules into the sensing layers. Yet, fabricating of coherent (i.e., crack-free) microporous membranes over larger areas remains challenging.^213^

**Figure 9:**
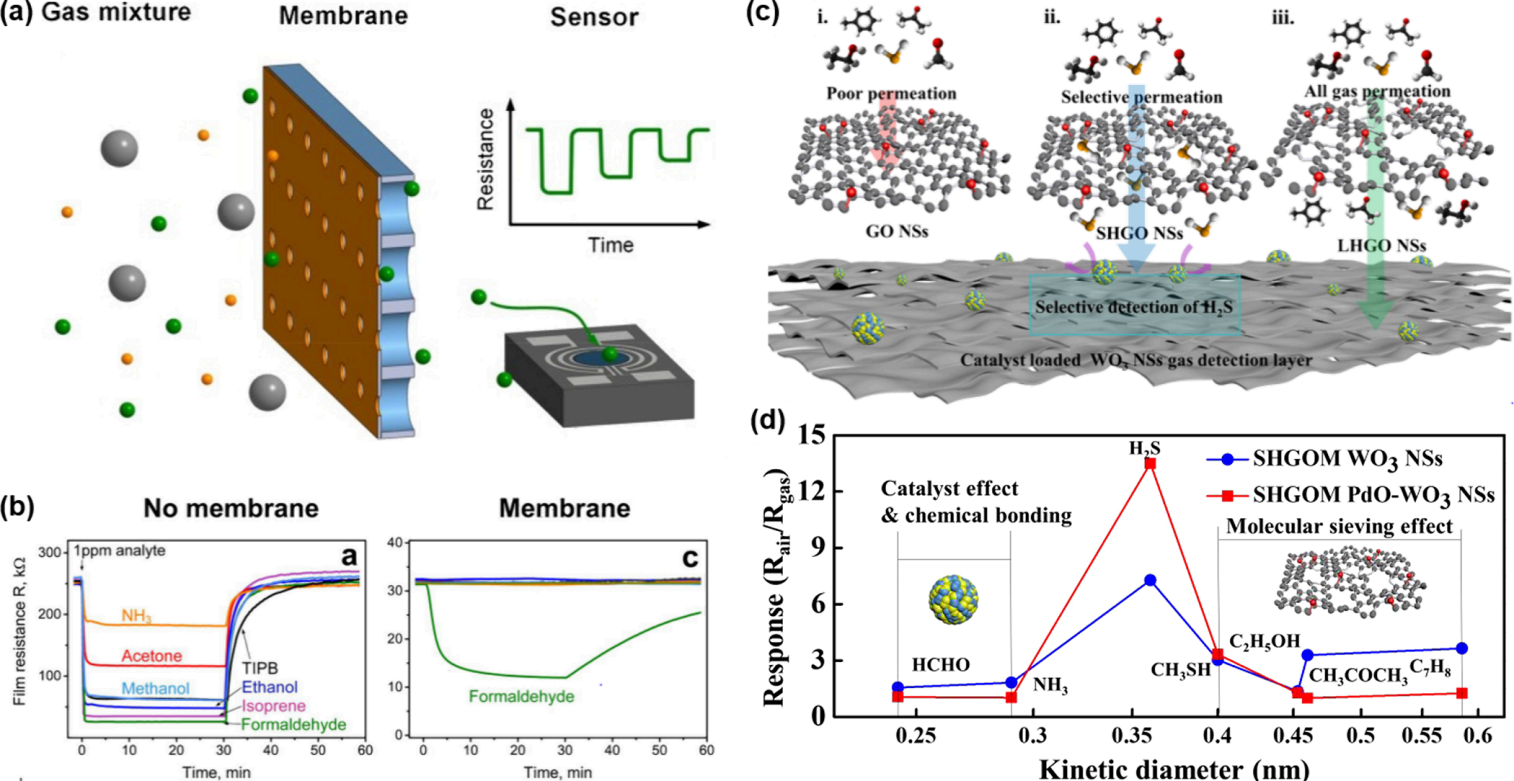
(a) Schematic of filter-sensor assembly. Gas mixtures are preseparated by a microporous filter, allowing only a target analyte (green) to reach the sensor placed downstream. (b) Sensor responses without and with zeolite membrane on Pd-doped SnO_2_ sensors. Reproduced with permission from ref (121). Copyright 2018 Elsevier. (c) Schematic of gas molecular sieving layer by using pore-size-tuned graphene oxide membranes. (d) Sensing responses of small hole–graphene oxide membrane-covered WO_3_ NSs and PdO-WO_3_ NSs sensors. Reproduced with permission from ref (214). Copyright 2020 American Chemical Society.

Various physical gas filtration effects were demonstrated by introducing nanoporous membrane filters on underlying metal oxide-based gas sensing layers. For example, Güntner et al.^212^ placed a zeolite mobile-five (MFI)/Al_2_O_3_ membrane upstream of a nonselective Pd-SnO_2_ sensor to infiltrate formaldehyde as schematically shown in Figure 9a. Since the size of the Al_2_O_3_-free MFI (0.510–0.580 nm) was larger than that of formaldehyde, it was possible that the MFI certainly converged to even smaller pore sizes upon integration with Al_2_O_3_. This composite filter exhibited unprecedented selectivity (up to 1000 times) toward 30 ppb of formaldehyde against 1,3,5-triisopropylbenzene (TIPB) (kinetic diameter of 0.840 nm), toluene, acetone, ethanol, and NH_3_ at 90% relative humidity (RH) compared to the bare Pd-SnO_2_ based sensor (Figure 9b). Yet, integrating Pd-SnO_2_ sensor with the MFI membrane increased the response and recovery times by a factor of 8 due to the MFI/Al_2_O_3_ membrane’s diffusion resistance.

In another strategy, the pore-size tuned GO is considered as an effective gas filtration membranes.^215,216^ Considering that pristine GO features an angstrom-sized pore distribution, effective strategies for tuning the pore-size of GO have been suggested by introducing O_2_ plasma treatment, physical sonication, and chemical etching methods to utilize as gas filtration applications.^217,218^ Jang et al. suggested that the pore-size distribution of GO can be precisely tuned by controlling the concentration of H_2_O_2_ solution treatment.^214^ As a result, small-hole-loaded (0.3–0.4 nm) GO membrane (SHGOM) and large-hole-loaded (0.51–0.52 nm) GO membrane (LHGOM) were prepared, and introduced on the PdO NPs-functionalized WO_3_ nanosheets sensing layers (Figure 9c). Note that the effective kinetic diameters of tested gases, i.e., HCHO, NH_3_, H_2_S, CH_3_SH, C_2_H_5_OH, CH_3_COCH_3_, and C_7_H_8_ are 0.24, 0.29, 0.36, 0.38, 0.45, 0.45, and 0.59 nm, respectively (Figure 9d). In this sense, SHGOM-loaded PdO-WO_3_ NSs sensing layers exhibited highly selective H_2_S sensing properties based on effective physical gas filtration effect.

**Figure 10:**
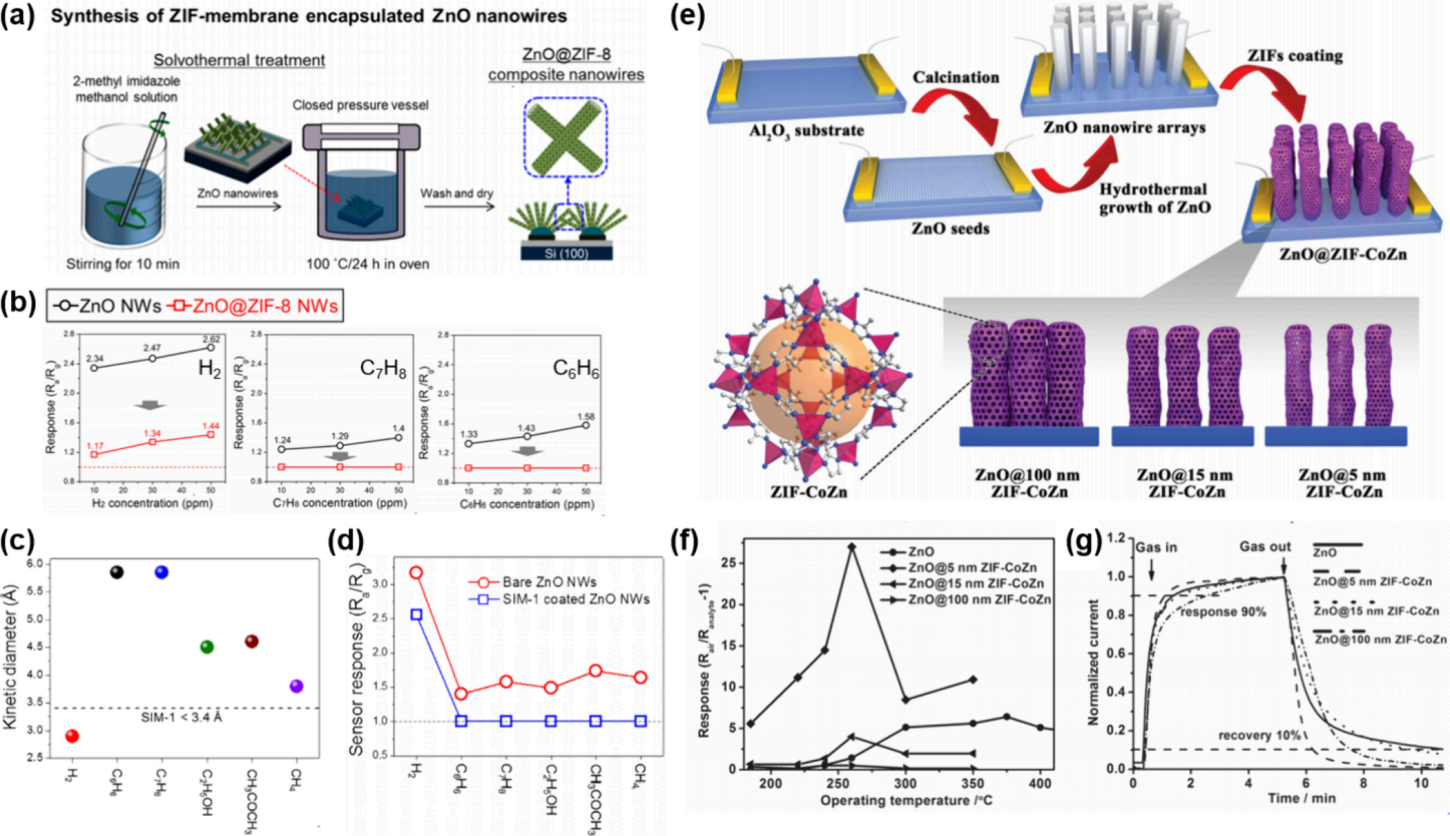
(a) Schematic of the synthesis steps of ZIF-membrane encapsulated ZnO nanowires (ZnO@ZIF-8). (b) Responses of the pristine ZnO nanowires and ZnO@ZIF-8 composite nanowires toward H_2_, C_7_H_8_, and C_6_H_6_ gas molecules. Reproduced with permission from ref (129). Copyright 2016 American Chemical Society. (c) Kinetic diameters of gas analytes in relation with the estimated pore aperture of SIM-1 metal organic framework material and (d) the corresponding sensor response values toward gas analytes using bare ZnO nanowires and SIM-1 coated ZnO nanowires. Reproduced with permission from ref (118). Copyright 2018 Elsevier. (e) Schematic of the synthesis of ZnO@ZIF-CoZn gas sensors. (f) Temperature-dependent responses and (g) response-recovery curves toward acetone molecules. Reproduced with permission from ref (223). Copyright 2016 Wiley-VCH.

In particular, MOFs such as zeolite imidazolate frameworks (ZIFs) have gained great attention as a new class of sieving materials with large surface area and numerous pores that endow them with notable molecular sieving capabilities.^219,220^ Thus, integrating these micro-/mesoporous membranes on highly sensitive SMOs-based sensing layers could yield sensors demonstrating remarkable selectivity. Ideally, such combination allows the separation of gas mixtures within the sieving layer, enabling the target gas to permeate downstream to the gas-sensing layer. Drobek et al.^221^ demonstrated the advantage of these features by coating ZnO NWs (≈85 nm thick and ≈25 μm long) with a thin ZIF-8 membrane for H_2_ sensing. The ZnO NWs were partially dissociated into Zn^2+^, followed by a reaction with 2-methylimidazole in methanol (at 100 °C) to form ZIF-8, the sieving membrane, while the NW-NW architectural contacts remained intact to maintain continuous electrical pathways (Figure 10a). Unlike a bare ZnO-based sensing layer, the ZnO@ZIF-8 based sensor showed remarkable selectivity toward H_2_, effectively filtering out toluene and benzene at 300 °C (Figure 10b). While gas molecules with kinetic diameters larger than the pore size of ZIF-8 of 0.340 nm (e.g., toluene and benzene are both 0.585 nm) cannot permeate through, the kinetic diameter of H_2_ is smaller (0.289 nm).^204,222^ In a similar work, the same group investigated the sieving of H_2_ against acetone, CH_4_, toluene, benzene, and ethanol using a substitutional imidazolate material-1 (SIM-1), a material that is isostructural with ZIF-8.^209^ The response of ZnO@SIM-1 to H_2_ was higher compared to that observed for the large-size molecules such as CH_4_, ethanol, acetone, toluene, and benzene (Figure 10c,d).

In another work, hydrophobic ZIF-CoZn thin-film was grown on ZnO nanorods via simple solution method with controlled thickness (5, 15, and 100 nm) of ZIF-CoZn membrane by changing the concentration of 2-methylimidazole (Figure 10e).^223^ The sensing tests were evaluated using pristine ZnO nanorods and ZIF-CoZn-coated ZnO nanorods from 200 to 350 °C toward 10 ppm acetone gas. Pristine ZnO nanorods exhibited the highest sensing response at high operating temperature of 375 °C, while ZIF-CoZn-coated ZnO nanorods showed relatively lower optimal temperature of 275 °C (Figure 10f). During acetone sensing at an elevated temperature, Co^2+^ ions in ZIF-CoZn partially oxidize into p-type CoO_*x*_ nanoparticles which can contribute to accelerate dissociation and activation of oxygen molecules due to the enhanced acetone oxidation reactions. Moreover, gas response and recovery speeds were accelerated based on catalytic effect of ZIF-CoZn membrane (Figure 10g). This work highlights that introduction of ZIF-CoZn membrane imparts not only gas selectivity via the physical filtration effect, but also enhanced response, reaction speeds, and lowering of optimal operating temperature. Very recently, Jo et al. proposed exclusive and ultrasensitive detection of HCHO gas at room temperature by introducing mixed matrix membrane composed of ZIF-7 nanoparticles and polymers on TiO_2_ sensing films.^224^ The selective detection of carcinogenic HCHO gas using oxide-based sensing layers without the interference of highly reactive ethanol has been a challenging issue. Based on effective physical molecular sieving effect to screen out ethanol gas, high selectivity (response ratio >50) and response (*R*_air_/*R*_gas_ > 1100) at 5 ppm of HCHO gas were obtained. The physical filtration approach is highly tailorable to different experimental contexts because available materials cover a wide range of pore sizes. This allows for selective separation of different gases by applying the appropriate filtration membranes. However, the additional permeation resistance of the physical filter has to be considered and can reduce sensitivity and slow down response kinetics.

#### 3.4.2 Catalytic Gas Filtration

The gas separation techniques introduced in the previous section depend only on the kinetic diameters of gases and pore sizes of the filtration membranes, which limit the practical scope of their application. In particular, gases of similar kinetic diameters will not be effectively discriminated by physical membranes. For example, water vapor has a kinetic diameter of 0.260 nm which is similar to that of H_2_ (0.289 nm) while being substantially smaller than kinetic diameters of most other gases. Therefore, even with the filtration membrane, the sensing surface would still exhibit poor cross-sensitivity to moisture in highly humid environments. Thus, apart from the physical filters introduced so far, catalytic filters, which involve the interfacing of SMOs with an overlayer (e.g., directly deposited on top of the sensing film by flame spray pyrolysis)^225,226^ or packed bed^227^ preceding the sensor and reactive to interfering gases, have also been investigated.^196,228^

**Figure 11:**
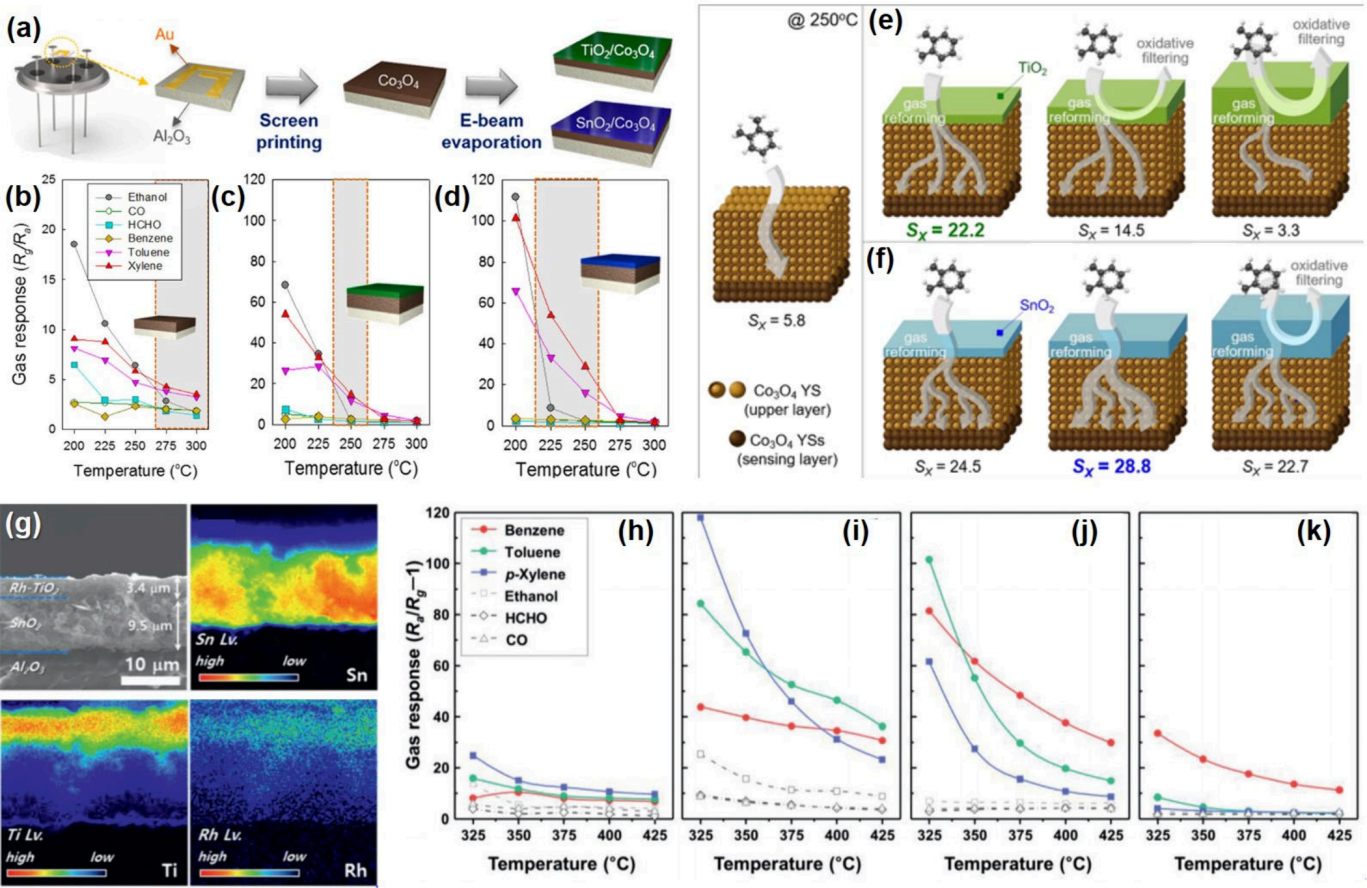
(a) Schematic diagram for the fabrication of Co_3_O_4_, TiO_2_/Co_3_O_4_, and SnO_2_/Co_3_O_4_ sensors. Gas-sensing characteristics and gas responses of (b) Co_3_O_4_ (c) 5 nm-thick TiO_2_/Co_3_O_4_, and (d) 5 nm thick SnO_2_/Co_3_O_4_ sensors to 5 ppm of various gases. Schematic diagram of mechanism of catalytic filtering effect during xylene sensing using (e) TiO_2_/Co_3_O_4_ and (f) SnO_2_/Co_3_O_4_ sensing layers. Reproduced with permission from ref (105). Copyright 2017 American Chemical Society. (g) Cross-sectional SEM image and electron probe microanalysis element mapping images of Rh-TiO_2_/SnO_2_ sensing layers. Sensing results of (h) pristine SnO_2_, (i) 0.5Rh-TiO_2_/SnO_2_, (j) 1Rh-TiO_2_/SnO_2_, and (k) 2Rh-TiO_2_/SnO_2_. Reproduced with permission from ref (230). Copyright 2021 Wiley-VCH.

To this end, coating SMOs-based overlayers on active gas sensing surfaces represents a viable method to filter out interfering species by their catalytic oxidation to inactive species. Jeong et al. coated a uniform SnO_2_ or TiO_2_ nanoscale overlayer on a screen-printed layer of mesoporous Co_3_O_4_ yolk–shell spheres (Figure 11a) to suppress the cross-sensitivity of Co_3_O_4_ toward interfering gas analytes.^229^ The coating of Co_3_O_4_ with TiO_2_ or SnO_2_ enhanced the response to xylene and toluene without affecting the p-type conduction mechanism of the Co_3_O_4_ underlayer (Figure 11b1–b3). The response and selectivity properties of the heterolayers varied with the thickness of the overlayers (in a range of 2–20 nm), which was attributed to the reformation and/or the oxidation of analytes. A comparison of sensing performance between xylene and ethanol at 250 °C showed that thinner (2 nm-thick) TiO_2_ and SnO_2_ overlayers catalytically transformed the less-reactive xylene into more reactive species as it diffused inward (Figure 11c1–c2). For thicker overlayers, both the reformation and the oxidative mechanisms were present, resulting in low response. Typically, ethanol and other interfering gases such as CO, formaldehyde, and benzene are more reactive than xylene; thus, they can be oxidized into less- or nonreactive species regardless of the overlayer type (SnO_2_ or TiO_2_), resulting in enhanced selective detection of xylene. However, this filtration setup still showed a significant response to toluene, implying that its use may be limited in the presence of toluene.

In contrast to this work, the same group had suggested an e-beam evaporation method of a catalytic Co_3_O_4_ overlayer (oxidative filter) onto Pd-SnO_2_ yolk–shell microspheres to achieve selective and sensitive detection of chemically stable benzene against toluene, *p*-xylene, ethanol, formaldehyde, and CO.^231^ Upon isolated exposures to these gases at 375 °C, the Co_3_O_4_-coated Pd-SnO_2_ microspheres converted the benzene into more reactive species followed by their subsequent transfer to the underlying sensing layer, while Co_3_O_4_ and Pd chemically filtered toluene, *p*-xylene, ethanol, formaldehyde, and CO into ineffective CO_2_ and H_2_O. For reference, bare Pd-SnO_2_ microspheres did not exhibit such sensing properties. In another work, the same group recently reported the effect of introducing Rh-TiO_2_ catalytic overlayer on SnO_2_ for the selective detection, discrimination, and analysis of aromatic compounds including *p*-xylene, benzene, and toluene analytes toward interfering VOCs including ethanol, formaldehyde, and carbon monoxide analytes.^230^ At first, SnO_2_ hollow spheres were prepared as sensing layers by ultrasonic spray pyrolysis and subsequent annealing process. After screen printing of SnO_2_ sensing layers on the Al_2_O_3_ sensor substrate, Rh-doped TiO_2_ powders were additionally coated on SnO_2_ layers. The successful construction of Rh-TiO_2_/SnO_2_ bistacked layers was confirmed by cross-sectional SEM and electron probe microanalysis element mapping images (Figure 11d). While the previous studies focused on the effect of the overlayer thickness, this work focused on the effect of the mass loading of catalytic Rh nanoparticles in a TiO_2_ overlayer (0.5, 1, and 2 wt %). The sensing tests were conducted using pure SnO_2_ and Rh-TiO_2_/SnO_2_ sensors (Figure 11e). Pure SnO_2_ sensors exhibited poor selectivity, while Rh-TiO_2_/SnO_2_ sensors showed precisely tunable gas selectivity toward *p*-xylene, toluene, and benzene analytes, dictated by the operating temperature and amount of Rh (Figure 11e1–e4). These results are attributed to the role of catalytic Rh-TiO_2_ overlayers, which either reform the gas analytes into more active species or completely oxidize the gas analytes into less-reactive forms.

Highest benzene selectivity (>200) over toluene, xylene, ketones, alcohols, and aldehydes has been achieved by a packed bed of catalytic WO_3_ NPs.^232^ As identified by pyridine adsorption, WO_3_ surfaces feature a high abundance of Lewis acid sites with stronger π-electron interactions with the aromatic rings of xylene and toluene than benzene. This is associated with the additional methyl groups of xylene and toluene that donate electrons to the aromatic ring.^233^ When applied as packed bed for prescreening a Pd-doped SnO_2_ sensor, benzene was detected down to 13 ppb with high robustness to 10–80% RH. When integrated into a hand-held device, this benzene was detected in spiked indoor air covering various national exposure limits (e.g., USA 100 ppb),^234^ as confirmed by proton transfer reaction time-of-flight mass spectrometry (PTR-TOF-MS). More recently, a packed bed of metastable CoCu_2_O_3_ nanocrystals produced by nonequilibrium flame spray pyrolysis has demonstrated similarly promising benzene selectivity in gas mixtures.^235^

Such catalytic packed beds are also quite effective for selective acetone sensing to remove critical confounders, for instance, ethanol in breath analysis.^236^ More specifically, a 0.2 mol % Pt/Al_2_O_3_ packed bed at 135 °C preceding a Si-doped ε-WO_3_ sensor enabled acetone sensing down to 50 ppb with high selectivity (>250) over H_2_, ethanol, isoprene, ammonia, and CO.^237^ This filter-sensor assembly quantified acetone accurately in 146 breath samples of volunteers during exercise with bias and precision of 25 and 169 ppb, respectively, as revealed by mass spectrometry.^238^ Note that the packed bed can be operated also at 40 °C by increasing the Pt content to 3 mol %.^239^

Overall, the flexibility to tune the type of sensing layer, overlayer thickness, catalyst content in overlayer or packed bed, and sensing temperature makes this approach more versatile than the physical sieving membranes. This is because the reactivity of the gases with the catalytic overlayers or packed beds can effectively discriminate different gas species that have similar kinetic diameters via gas reforming and oxidative filtering reactions.

#### 3.4.3 Sorption Gas Filtration

**Figure 12:**
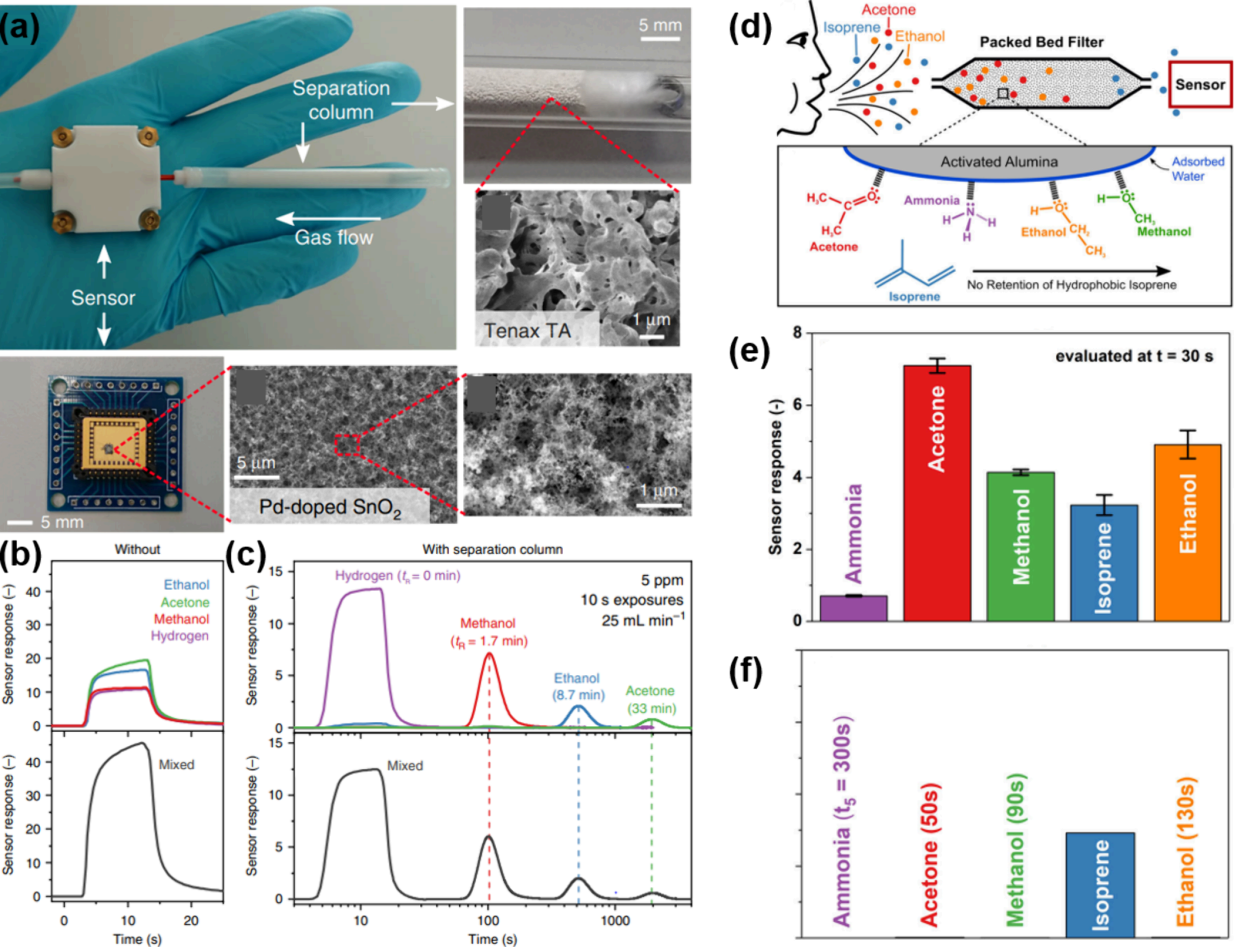
(a) Images of the hand-held methanol detector which consists of Pd-doped SnO_2_ sensing layers connected with separation column (Tenax TA). Responses toward 10 s exposures to 5 ppm ethanol, acetone, methanol, and hydrogen gases as well as their mixtures (b) without and (c) with Tenax TA separation column. Reproduced with permission from ref (242). Copyright 2019 Springer Nature Group. (d) Schematic of filter–sensor concept for selective detection of isoprene in complex gas mixtures. The breath is filtered through a packed bed filter placed upstream of a highly sensitive but nonspecific gas sensor. Unlike hydrophobic isoprene, which does not interact with the filter, hydrophilic analytes are adsorbed and retained on the activated alumina and on the adsorbed water layer. (e) Selectivity of a single Pt-doped SnO_2_ sensor without a filter after 30 s of introducing analyte gases, and (f) selectivity of a Pt-doped SnO_2_ sensor integrated with activated alumina filter after 30 s of introducing gas analytes. Note that (e) and (f) share the same vertical scale. Reproduced with permission from ref (103). Copyright 2018 American Chemical Society.

In addition to physical and catalytic gas filtration membranes, sorption-type filters have been widely studied using various materials such as carbon-based materials (e.g., activated carbon, graphene),^240^ porous polymers (e.g., Tenax TA),^241^ silica, activated alumina, MOFs, and zeolites, which feature high porosity and large surface area. The filtration efficiency can be varied depending on the surface groups (polar, nonpolar, or analyte specific), surface area, and porosity of adsorbent filters. In the case of nonpolar sorption-type filters, adsorption takes place through nonspecific dispersion physical forces, i.e., weak van der Waals, which are proportional to the molecular weight of gas analytes. In addition, the gas diffusivity or flow rate of gas analytes are also affected by the molecular weight of gas analytes. In this sense, time-dependent gas discrimination properties can be obtained using nonpolar sorption filters which are analogous to the gas chromatography column system. Broek et al. reported a hand-held gas sensor which can selectively detect methanol over ethanol by introducing nonpolar Tenax-based separation column in front of the sensors coated with Pd-doped SnO_2_ nanoparticles (Figure 12a).^242^ The separation column is formed by a packed bed of Tenax TA particles as the stationary phase, somehow similar to a miniaturized gas chromatography column. The sensing tests were conducted using sensors with and without introduction of the separation column. Pristine Pd-doped SnO_2_ sensor without the separation column exhibited nonselective sensing properties toward 5 ppm ethanol, acetone, methanol, and hydrogen analytes with responses of about 10–20 (Figure 12b). Upon exposure to mixed gases, the sensor response increased up to 45, showing that the sensor cannot differentiate the target gas (methanol) in the presence of interfering gas analytes. When the Pd-doped SnO_2_ sensor is combined with the separation column, four different gas analytes were clearly differentiated with distinct separation of sensor responses as in chromatography (Figure 12c), enabling selective multitracer detection. Since larger gas molecules feature higher van der Waals force with Tenax filters, the gas response and retention time depends upon the type of gas analytes. In this regard, excellent selectivity was obtained despite the slight retention toward methanol gas in the separation column that reduced the sensor response. This sensor concept has been readily integrated into a hand-held device^101^ and tested successfully for the analysis of alcoholic beverages^97^ and hand sanitizers^98^ during COVID-19 for methanol contamination and even for intoxication screening from exhaled human breath.^99^

Meanwhile, polar sorption-type filters adsorb gas analytes through dipole–dipole or hydrogen bonding reactions. Recently, Broek et al.^243^ introduced a porous, activated Al_2_O_3_ filter on the upstream of Pt-doped SnO_2_ sensor film, which is capable of separating concentrated breath mixtures into hydrophilic compounds (ammonia, acetone, methanol, and ethanol) and hydrophobic isoprene (Figure 12d). Unlike the nonselective bare Pt-SnO_2_ sensor film, the Al_2_O_3_-attached Pt-SnO_2_ sensor film exhibited high isoprene selectivity (>100) against the interfering species and fast detection (response time = 4.2 s) of 500 ppb in a highly humid ambient (90% RH) after a typical breath test that was conducted for 30 s (Figure 12e,f). Typically, Al_2_O_3_ possesses polar sites because of unsaturated (electron deficient) Al and O atoms, rendering it hydrophilic and able to filter out many hydrophilic gas species by hydrogen bonding.^244^ Combining such activated Al_2_O_3_ with Si-doped ε-WO_3_ sensors quantified the breath isoprene dynamics in volunteers during muscle activity and rest.^245^ In applications where several hydrophobic gases are present at elevated concentrations, such hydrophilic filters may need to be combined with other concepts, like size-selective zeolites^221^ or perforated graphene.^205^Table 3 summarizes some of the recently reported gas sensing materials as well as their corresponding filtration membranes and sensing performances.

**Table 3:** Recently Reported Semiconducting Metal Oxides Based Gas Sensors Integrated with Various Physical/Chemical Filters

sensing material	sieving/filtration membrane	response definition	response/conc (ppm)	target gas	interferents	ambient condition	ref
ZnO NWs	ZIF-8	*R*_a_/*R*_g_	2.64–50 ppm	H_2_	toluene and benzene	air, 300 °C	(221)
Pt-doped SnO_2_	activated Al_2_O_3_	(*R*_a_/*R*_g_) – 1	∼3 at 500 ppb	isoprene	acetone, NH_3_, and ethanol, methanol	air, 400 °C	(243)
WO_3_	indigo carmine	*R*_a_/*R*_g_	58% for 1 ppm	NO_2_	ozone	air, 275 °C	(246)
In_2_O_3_ NFs	ZIF-8	*R*_g_/*R*_a_	16.4 at 1 ppm	NO_2_	moisture	N_2_, 140 °C	(247)
Pd-doped SnO_2_ NPs	MFI/Al_2_O_3_	(*R*_a_/*R*_g_) – 1	1.85 at 1 ppm	HCHO	TIPB, isoprene, acetone, ethanol, and methanol	air or N_2_, 400 °C	(212)
Co_3_O_4_ spheres	SnO_2_	*R*_a_/*R*_g_	28.8 at 5 ppm	xylene	ethanol, HCHO, toluene, CO, and benzene	N_2_/air, 250 °C	(229)
WO_3_ powder	H-ZSM-5	*R*_g_/*R*_a_	107 at 400 ppb	NO_2_	CO	air, 350 °C	(248)
Pt@In_2_O_3_ NFs	SBA-15	*R*_a_/*R*_g_	14 at 5 ppm	acetone	hydrothion, NH_3_, 1-hexanol, NO_2_, toluene, ethanol, methanol, and humidity	N_2_, 340 °C	(249)
ZnO nanorods	ZIF-8	(*R*_a_*R*_g_)/*R*_a_	34.35% at 5 ppm	H_2_S	H_2_, CO, and CH_4_	air, 25 °C	(250)
SnO_2_ film	APTES-ester	G/Go	1.45 at 100 ppm	NH_3_	ethanol and acetone	air/N_2_, 25 °C	(251)
Si-doped ε-WO_3_	Tenax	(*R*_a_/*R*_g_) – 1	1.1 at 250 ppb	limonene	acetone, 2-propanol, methanol, hydrogen,	air, 25 °C	(252)
Pd-doped SnO_2_	Tenax	(*R*_a_/*R*_g_) – 1	∼7 at 5 ppm	methanol	hydrogen, ethanol, acetone	air, 25 °C	
Pd-doped SnO_2_	Tenax	(*R*_a_/*R*_g_) – 1	0.9 at 500 ppb	formaldehyde	hydrogen, methanol, acetaldehyde, ethanol	air, 25 °C	
Si-doped ε-WO_3_	Pt/Al_2_O_2_	(*R*_a_/*R*_g_) – 1	4.2 at 1 ppm	acetone	hydrogen, ethanol, isoprene, ammonia, CO	air, 135 °C (filter)	(237)
Pd-doped SnO_2_	WO_3_	(*R*_a_/*R*_g_) – 1	1.98 at 1 ppm	benzene	xylene, toluene, acetone, acetaldehyde, isoprene, methanol, ethanol, CO, hydrogen, formaldehyde	air, 240 °C (filter)	(232)

#### 3.4.4 Future Prospects in Integration of Metal Oxides with Gas Separation Filters

Gas separation filters offer an effective approach to mitigate cross-sensitivity in SMO-based chemiresistors, particularly when multiple gases are present in a complex mixture. By selectively filtering out interfering gases before they reach the sensing layer, separation filters can enhance the apparent selectivity of SMO sensors. However, their integration presents certain challenges, such as increased response time due to gas diffusion delays and additional system complexity.

Recent advances in filter materials, such as microporous or nanoporous membranes and functionalized coatings that were described in the previous sections, have shown promise in improving gas separation efficiency while minimizing these trade-offs. For example, porous materials tailored with specific adsorption properties can preferentially separate target gases from interfering species, allowing for a more controlled and selective gas sensing process. Further research into optimizing filter structures, such as hierarchical pore designs or hybrid material coatings, is critical to balancing gas selectivity and response time.

While intrinsic material design remains a primary focus for improving SMO-based gas sensor selectivity, integrating gas separation filters offers a complementary strategy for mitigating cross-sensitivity in complex environments. Future advancements in filter materials and structures, such as nanoporous membranes, functionalized coatings, and hierarchical architectures, will be essential for achieving selective gas separation without significantly compromising response time. Additionally, addressing challenges such as system complexity and ensuring compatibility with miniaturized sensors will be critical for practical applications. Combining these approaches with novel SMO sensor materials could lead to highly selective and efficient gas sensing systems.

That being said, there are still many challenges to the filter integrations. Filter solutions may not improve the humidity interference that most SMO sensing materials (e.g., SnO_2_)^253^ suffer from. For example, moisture passed the aforementioned zeolite/Al_2_O_3_ membrane,^212^ so higher humidity levels still lowered the sensor’s response to formaldehyde. This implies that the humidity can constantly affect the sensing performance regardless of the existence of the filtration membrane. A pronounced effect can be observed when SMOs, such as SnO_2_, with some degree of cross-sensitivity to moisture are used as sensing layers. To this end, membranes such as zeolites (e.g., MFI) and MOFs (e.g., ZIF-8) could be explored, as they possess internal acidic sites that can react with gas mixtures even before reaching the sensing layer surface.^212,254^ Another problem is the stacking of filtration membranes. For example, the two-dimensional GO sheets readily form a stacked structure, which hinders the movement of the target gases through the GO membrane and degrades the response value and selectivity.^204^ Since more permeable membranes are less selective and *vice versa*,^205,210,255^ an optimal number of GO layers needs to be assembled without affecting the diffusion kinetics as well as the sensor’s response and recovery speed. What is more, sieving efficiency of 2D materials depends on ordering of nanochannels on neighboring stacked sheets. This implies that the filter design and the structure/composition can both influence sensor’s selectivity.

Ongoing and future research may certainly consider preseparation of gas mixtures by adopting a judicious combination of chemical and physical filter on the same sensing layer. One such strategy is to decorate gas-specific reactive particles on physical sieving membranes to additionally confer chemical filtration properties. For instance, CaO, CuO, and BaO show outstanding affinity to CO_2_, and react quickly to form CaCO_3_, CuCO_3_, and BaCO_3_, respectively.^256,257^ Thus, under controlled life cycle performance,^258^ CaO, CuO, and BaO could be useful chemical filters in sensor systems where CO_2_ easily interferes with intended analyte. Another option could involve sandwiching a chemical filter between a physical filter (as a top layer) and an active sensing material placed downstream, or expanding this design to incorporate multiple intercalated layers. In a broader sense, implementing these approaches could mediate selectivity adjustment and optimization, independently of sensor design and composition. However, it is important to consider the factors such as the dependence of the permeance on temperature^210,254^ and poor stability of the membranes at relatively high temperatures (the operating temperature ranges of most SMO-based chemiresistive sensors) to realize physical membranes with improved selectivities.

### 3.5 Overall Challenges in Semiconducting Metal Oxides-Based Chemiresistive Sensors

Pristine SMOs have critical limitations that hinder the efforts to improve their gas sensing performances, such as limited receptor availability and nonspecific absorption when exposed to many analytes. Despite many endeavors to address these limitations, deviations in the selectivity still exist due to several factors such as the loading amount and the shape of catalysts, the morphology and crystallite size of SMOs, the types of defects, the presence of impurities, and the sensing conditions (humidity and temperature).^38^ In humid environments, water molecules compete with target gases for adsorption sites, reducing response and causing baseline resistance drift. Elevated temperatures, while improving response and recovery speeds, can lead to the agglomeration of catalytic nanoparticles, thereby diminishing long-term stability of the sensors. Innovative approaches, such as humidity-resistant coatings like CeO_2_ nanoclusters, dynamically stabilize adsorbed oxygen ions and mitigate the effects of humidity. Similarly, ex-solved catalytic nanoparticles enhance high-temperature stability and provide resistance to sulfur-containing gas analytes. These nanoparticles remain strongly anchored in the matrix, preventing agglomeration and maintaining sensor performance under harsh conditions. Moreover, the size and distribution of catalytic NPs on SMOs surfaces are major factors for sensitivity enhancement that have much room for further optimization. On the other hand, the selectivity of SMO-based sensors can be easily degraded by the presence of impurities.^19,20^ An example is given by Pt-loaded SnO_2_ NFs/nanotubes (NTs), which are well-known for selective acetone sensing.^14,15,259^ However, trace impurities of SiO_2_ in Pt-decorated SnO_2_ NTs act as promotors for selective detection of H_2_S, which in turn devalues the acetone selectivity of the sensor. Ideally, SiO_2_ impurities should not detect H_2_S but only act as localized moisture adsorption sites, while H_2_S can preferentially interact with the SnO_2_ surface because H–S bond is weaker than the H–O bond in moisture.^20^ It can be concluded that further investigation is required in understanding the selectivity mechanisms of SMOs in general, with respect to (1) the amount, composition, and shape of catalysts, (2) the morphology of host SMOs, and (3) the sensing conditions. These strategies, combined with ongoing material innovations, aim to achieve reliable performance and balanced selectivity, response, and response kinetics in diverse and harsh environmental conditions.

## 4 Selectivity in Metal- and Nonmetal-Based Chemiresistive Sensors

Performance metrics such as response, selectivity, and response/recovery rates of metal- and nonmetal-based sensors depend on the nature of the elements’ reactivity upon interaction with gas analytes. While many elements have negligible affinities to gas molecules or show very weak chemical interactions, exceptional candidates such as palladium, platinum, and silicon can exhibit dramatic selective sensing properties toward various gases.^260−262^ As shown in Table 4, different morphologies of materials in this category can be synthesized using a variety of innovative ways and show great promise for use as chemiresistive sensors displaying high selectivity. In this part, we limit our discussion to research endeavors dedicated to application of pristine metals, nonmetals, and metal alloys/composites as chemiresistive chemical sensors with specific selectivities.

**Table 4:** Recently Reported Gas-Selective Metal and Metal Alloy/Composite Based Chemiresistive Sensors

sensing material	response definition	response/conc (ppm)	target gas	interferents	ambient condition	ref
Pd NP/single layer graphene hybrid with PMMA sieve	Δ*R*/*R*_O_	66.37% for 2%	H_2_	methane, CO, and NO_2_	N_2_, RT	(263)
Pd NPs film with PMMA sieve	Δ*G*/*G*_O_	2.9% at 1000 ppm^a^	H_2_	CO and CH_4_	CO/CH_4_/N_2_, RT	(264)
Pd NWs with ZIF-8 sieve	Δ*R*/*R*_O_	3.55 for 1%	H_2_	O_2_ and N_2_	air, RT	(222)
Pd hemitubes	Δ*R*/*R*_O_	2.1% for 1%	H_2_	NH_3_, CO, NO_2_, and H_2_S	N_2_/air (4:1), RT	(265)
Pd-decorated Si mesh	[(*I* – *I*_0_)/*I*_0_]	10% at 0.1%	H_2_	H_2_S, NO_2_, toluene, CO, and ethanol	N_2_/air (4:1), RT	(266)
Pd-capped Mg bimetallic film	[(*R*_g_ – *R*_a_)/*R*_a_]	≈3.5% at 1%	H_2_	N_2_, CO_2_, CO, NO_2_, and O_2_	N_2_, RT	(267)
Pt NWs networks	Δ*G*/*G*_O_	261.4 ± 46.5% at 0.5%	H_2_	humidity vapor and CO	air, RT	(268)
Pd_0.75_–Au_0.25_ NWs	Δ*I*/*I*_O_	88% at 1%	H_2_	not investigated	Ar, RT	(269)
Pd/MgPd alloy film	Δ*R*%	3.3% at 2 bar	H_2_	not investigated	Ar, RT	(270)
Ag-modified Si NWs	*R*_a_/*R*_g_	1.8–0.33 ppm	NH_3_	H_2_, ethanol, acetone, methane, and methanol	air, RT	(271)
porous Si NWs	*R*_g_/*R*_a_	1.16–10 ppm	NO_2_	H_2_, SO_2_, ethanol, toluene, and benzene	air, RT	(272)
Pd NP film	Δ*I*/*I*_O_	≈5.5% for 0.1%	H_2_	not investigated	Air, RT	(273)
Pd–Ag hollow NWs	Δ*R*/*R*_O_	0.89% at 900 ppm	H_2_	not investigated	air, RT	(117)
Pd coated Si NWs	Δ*G*/*G*_O_	>300% for 2%	H_2_	not investigated	air, RT	(274)
Pd_0.92_–Y_0.08_ alloy nanosheets	Δ*R*/*R*_O_	18.67% for 0.5%	H_2_	not investigated	N_2_, RT	(275)
Al/p-Si/Al structure	*R*_g_/*R*_a_	≈1.3 at 5 ppm	NH_3_	CH_4_, H_2_, ethanol, acetone, HCl	air, RT	(276)

a concentration of gas mixtures.

### 4.1 Pure Metal-Based Chemiresistive Sensors

#### 4.1.1 Palladium-Based Chemiresistors

**Figure 13:**
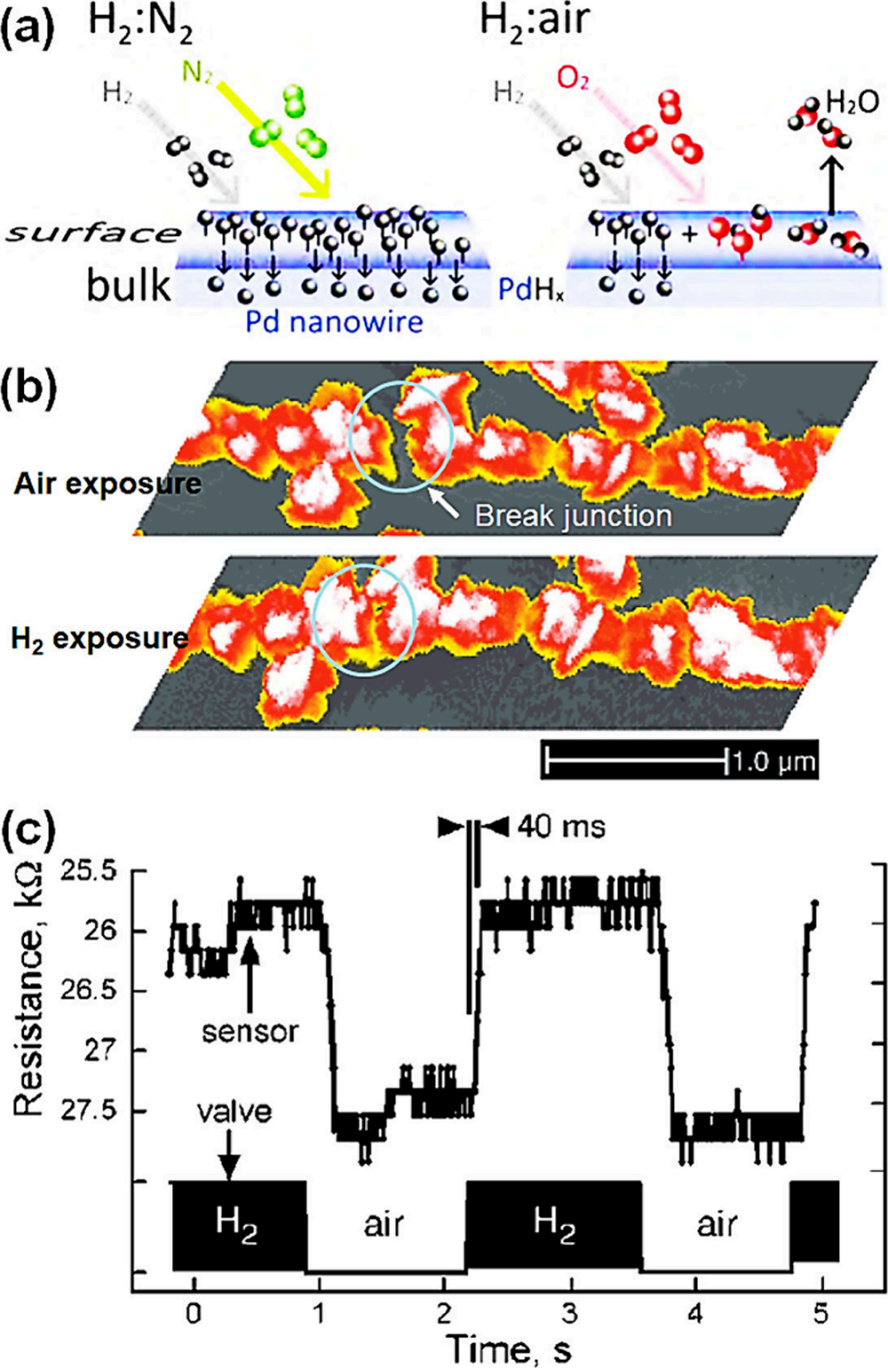
(a) A schematic indicating the influence of oxygen in air on the response of Pd NWs toward H_2_. The response of the NWs to H_2_ and the speed of the response and recovery of the resistance decrease in air as compared with N_2_. Reproduced with permission from ref (202). Copyright 2015 American Chemical Society. (b) AFM images of Pd mesowires in air and in a stream of hydrogen gas, respectively, (c) sensor resistance versus time response obtained upon on–off hydrogen exposures. During the off state, air was introduced. Reproduced with permission from ref (280). Copyright 2002 American Chemical Society.

Palladium (Pd) is among the most widely investigated metal-based materials for chemiresistive sensing. Hitherto, ubiquitous investigations are triggered by a strong affinity of Pd toward H_2_ while being relatively inert to other species. Compressed H_2_ is prone to leakage, posing an explosion hazard at concentrations exceeding 4%, making its early detection very important for safe handling. Ideally, Pd reacts with H_2_ reversibly to form palladium hydride (PdH_*x*_), which exhibits higher resistivity than pure Pd,^277,278^ the amount of which depends on the ambient of Pd exposure.^202^ In N_2_ ambient for example, only the adsorption of H_2_ onto Pd takes place followed by their dissociation and chemisorption to form Pd–H, whereas catalytic water formation takes place on Pd surface in air (oxygen) ambient, reducing the number of adsorption sites for H_2_ (Figure 13a). However, without H_2_ exposure, only atmospheric oxygen can be adsorbed, accompanied by a reduction in Pd resistivity. Adsorption of H_2_ on Pd in air, and subsequent formation of water occur according to reactions in eqs 10 and 11.^279^
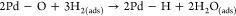
10
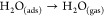
11

Two crucial phases of PdH_*x*_ can be distinguished when Pd is exposed to H_2_: the formation of α-phase when H_2_ gas diffuses into interstitial site of Pd (*x* < 0.015 for H_2_ concentrations <1%) and the formation of β-phase when H_2_ gas diffuses into substitutional site of Pd (0.015 < *x* < 0.7 for H_2_ concentrations >1%).^202,280−282^ Formation of the β-phase PdH_*x*_ leads to volumetric expansion, which could even lead to fracture in the case of Pd NWs.^281^ It should be noted that the fracture of Pd NWs/films produced by H_2_ exposure and subsequent α–β phase transitions brings about a H_2_ sensing mechanism of its kind, which has been widely elucidated in literature.^278,280,283^ Typically, however, nanosized gaps/pores are intentionally introduced at the time of Pd NWs/films fabrication to prevent such fractures for reliable sensor operation. For example, arrays of mesoscopic Pd NWs prepared by electrodeposition were shown to act as hydrogen-activated ‘‘on–off” switches when exposed to hydrogen.^278,280^ Atomic force microscopy studies confirmed that the discontinuities (or rather “break junctions”) in Pd meso-wires can be restored upon desorption of H_2_, and subsequent contraction of Pd lattice in ambient air, further supporting the “on–off” mechanism.^280^ This is to say, the closing and the reopening of the nanogaps in mesoscopic Pd NWs occur in a cyclic manner following H_2_ and air exposure, respectively (Figure 13b). Thereby, the resistance to the follow-on current in the external circuit decreases/increases when the flow of H_2_ is on/off (Figure 13c), respectively, and the nanorods could be connected and disconnected within a second. The closure of mesopores or intentionally introduced nanogaps upon formation of the β-PdH_*x*_ phase completes the electrical circuit; the intensity of the current or extent of nanogap closure depends on concentrations of H_2_ exposure. From this standpoint, a threshold of detectable concentrations is the limiting factor of this approach. Advanced demonstrations of this H_2_ sensing mechanism approach employ flexible substrates, with either sputtered, spin-coated or electrodeposited Pd nanoparticles.^284−286^ Moreover, Shim et al. introduced nanogap-controlled coating of metal oxides/silicon with Pd.^287^ While their results still indicate that H_2_ concentrations below 1% are insufficient to close the nanogaps or even trigger α-β phase transition when the nanorods are distantly spaced or nanogaps are significantly wide, smaller nanogaps between adjacent Pd-coated nanorods can be readily closed even upon exposure to diminutive H_2_ concentrations, triggering continuity of the electrical path among nanowires. By optimizing the size of the nanogap, ultrafast response and recovery (≈2.8 and <1 s on average, respectively) were achieved toward concentrations of H_2_ in the range of 0–2.0%. For some synthetic practices where Pd nanorods/NPs are grown on substrates (such those coated with Au), unstable ohmic contacts (Au–Pd contacts) are expected during the formation of a high resistivity PdH_*x*_, adding fluctuations to the sensor signals and impairing the sensor response/recovery rates.^288^ However, in other practical demonstrations, response and recovery rates can be improved by joule self-heating of Pd NWs^289^ that elevates the nanowire temperature’s, which accelerates the H_2_ adsorption, diffusion, and desorption on and from Pd surface. Therefore, sensing characteristics of Pd structures can be tuned by structure, morphology control, heating, and appropriate choice of synthetic method.^287^

Detection of H_2_ over a broader range of concentrations with ultralow LOD is possible by taking advantage of the resistivity variations in a continuous Pd sensing element or film.^117,260,288^ Sequential exposure of Pd nanostructures to pristine air and H_2_-containing environments show opposing effects as elucidated in eq 10; the resistivity of Pd NWs decreases or increases, respectively. Accordingly, the sensor response correlates linearly with the concentration of H_2_ until a plateau is reached.^288^ The uniqueness of this chemiresistive interaction of H_2_ with Pd is the basis for selective H_2_ sensing while remaining insensitive to a variety of other gases.^281^ To demonstrate this approach, Yang and co-workers utilized lithographically patterned nanowire electrodeposition (LPNE) technique to electrodeposit H_2_-sensitive Pd NWs on substrates.^260^ To avoid lattice expansion and fracture of NWs during α–β phase transition, electrodeposition was carried out from an EDTA (ethylenediamine tetraacetic acid)-containing Pd precursor solution under conditions favoring direct formation of the β–phase PdH_*x*_. The results revealed that, as the thickness of the electrodeposited NWs got smaller, the response to H_2_ increased to about 2–3 times that of larger NWs for exposures less than 1% H_2_. In addition, Pd NWs with smaller diameters exhibited rather faster and higher response values than that of thicker NWs. This is because the saturation rate of Pd grains by H_2_ depends on the thickness of Pd NWs. Furthermore, response and recovery speeds of Pd NWs toward H_2_ (5 ppm–10%) were faster compared to Pd-based films of the same thickness.

**Figure 14:**
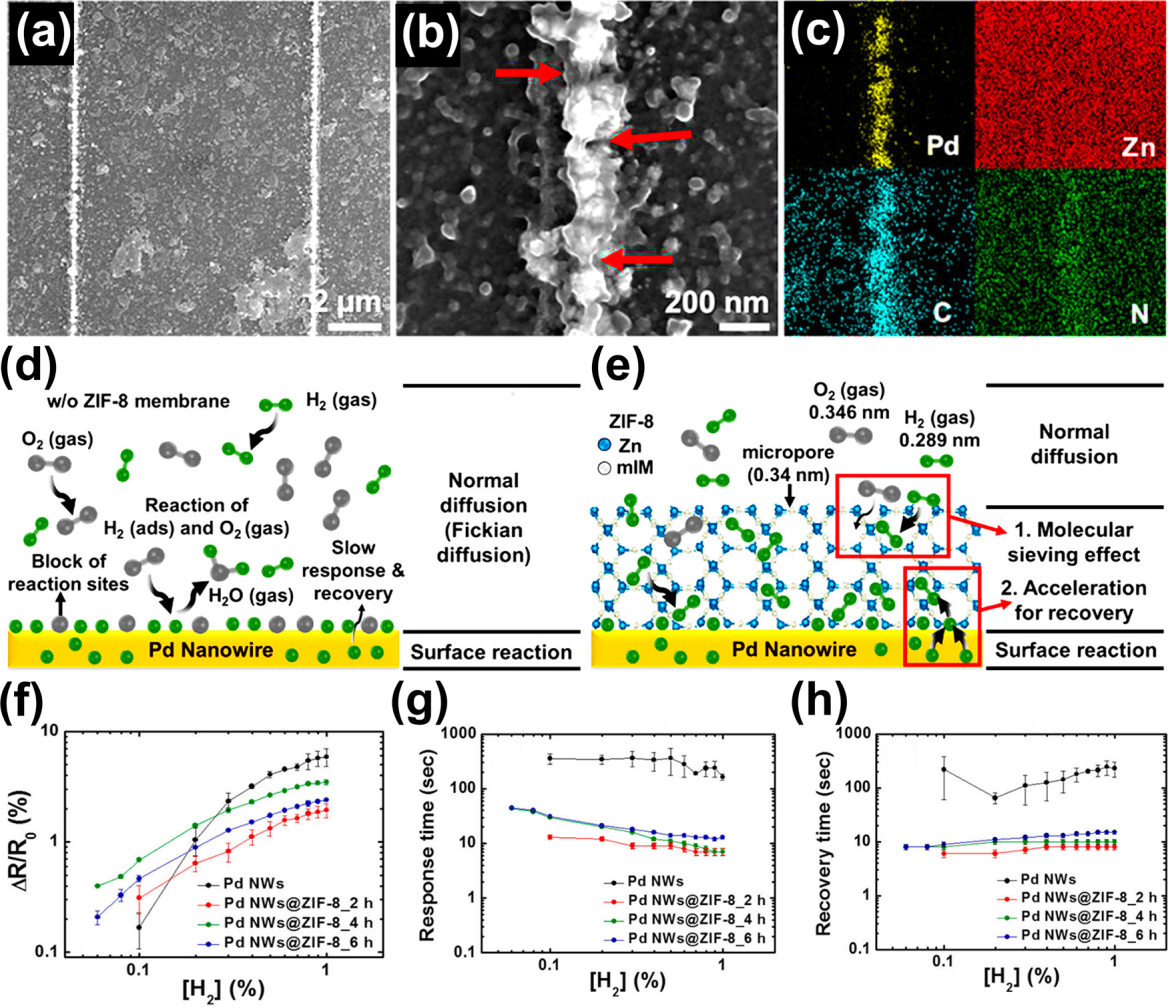
(a–c) SEM and EDS elemental images of Pd NWs@ZIF-8_2 h. (d,e) Schematics of the sensing model for Pd NWs (d) without and (c) with ZIF-8 membrane. The ZIF-8 (0.34 nm in size) membrane filters out O_2_ (0.346 nm), allowing only H_2_ (0.29 nm) to reach the Pd NWs. (f) Response, (g) response times, and (h) recovery times of Pd NWs and Pd NWs/ZIF-8 against H_2_ concentrations in air. Various thicknesses of ZIF-8 onto Pd NWs were achieved by varying the assembly time at 2, 4, and 6 h. Reproduced with permission from ref (222). Copyright 2017 American Chemical Society.

Typically, some gas species such as O_2_, CO, H_2_S, NH_3_, and H_2_O can irreversibly chemisorb on metal surfaces, impairing their sensing properties.^266,289^ For example, O_2_ adsorbed on Pd blocks active sites for adsorption of H_2_, leading to poorer response and recovery characteristics. Likewise, the adsorption of water vapor (H_2_O) and the catalytic formation of water on the Pd surface both increase the LOD. To address this issue, Koo et al.^222^ proposed the integration of LPNE-grown Pd NWs with a multipored ZIF-8 based nanofiltration layer (i.e., Pd NWs@ZIF-8) to screen out the interfering O_2_. Pd NWs were patterned on glass substrates and then were coated with ZIF-8 for 2 h (Figure 14a,b), uniformly covering the entire surface of Pd NWs and the substrate (Figure 14c). During exposure of the Pd NWs@ZIF-8 to H_2_ (kinetic diameter = 0.289 nm) in air ambient, ZIF-8 (pore size of 0.34 nm) selectively allowed permeation of H_2_ to Pd NWs while blocking O_2_ (kinetic diameter = 0.346 nm) in air (Figure 14d,e). Although the response of bare Pd NWs remained higher (Δ*R*/Δ*R*_max_ = 5.9%) than that of Pd NWs@ZIF-8 (Δ*R*/Δ*R*_max_ = 3.5%) toward 1% H_2_ (Figure 14f), the response time of Pd NWs decreased from 164 to 7 s for Pd NWs@ZIF-8 (Figure 14g). Similarly, the recovery time decreased from 229 to 10 s for Pd NWs and Pd NWs@ZIF-8, respectively (Figure 14h).

Pd is among the most extensively studied metal-based materials for chemiresistive sensing, particularly for hydrogen detection, due to its strong affinity for H_2_, which leads to the formation of PdH_*x*_, and its relative inertness to other chemical species. However, in air, adsorbed oxygen and the catalytic formation of water on the Pd surface can block active sites, impeding H_2_ adsorption and degrading response and recovery characteristics. To address these challenges, molecular sieving membranes can be employed to selectively block larger molecules (e.g., O_2_ and N_2_) while allowing H_2_ to penetrate, thereby enhancing selectivity, response, and response speed. At elevated temperatures, Pd-based sensors exhibit faster response and recovery speeds but face challenges such as an increased limit of detection due to the reduced solubility of H_2_.^290,291^ Furthermore, repeated exposure to high concentrations of H_2_ (>4%) can cause cyclic volume expansion and contraction associated with the α-to-β phase transition of PdH_*x*_.^278^ This repetitive structural stress can lead to cracking and degradation of the sensor over time. To improve durability, flexible polymer-based substrates incorporating Pd with nanogaps have been developed.^292,293^ These nanogaps effectively relieve stress and accommodate volume changes, enhancing the sensor’s long-term stability under harsh conditions. Such strategies highlight the inherent trade-offs between selectivity, response, and long-term stability in metal-based chemiresistive sensors, particularly under harsh operating conditions (e.g., elevated temperatures, repeated exposure to high concentrations of H_2_), underscoring the need for optimization to achieve reliable performance in challenging environments.

#### 4.1.2 Platinum-Based Chemiresistors

**Figure 15:**
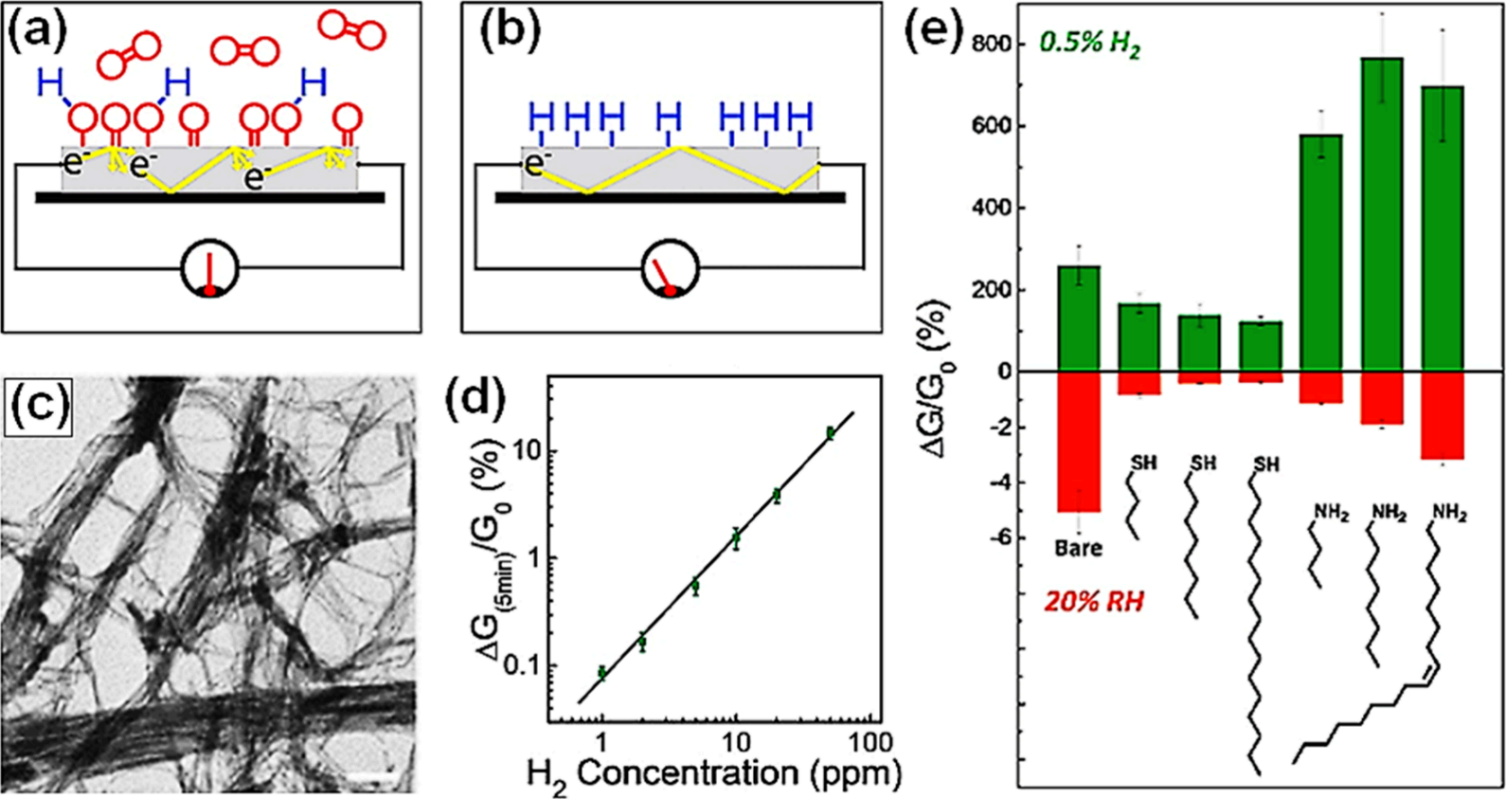
(a) Interaction of hydrogen with oxygen-terminated Pt surface in air and (b) hydrogen-terminated Pt surfaces. Formation of hydrogen-terminated surface leads to prominent diffuse electron scattering compared to oxygen-terminated surface. Reproduced with permission from ref (261). Copyright 2012 American Chemical Society. (c) TEM image of as-prepared ultrathin Pt NWs with diameter of ≈3 nm, and responses of Pt NWs (d) upon 5 min exposure to various concentrations of argon-balanced hydrogen gas and (e) with different ligand coating upon 30 min exposure to 0.5% argon-balanced hydrogen gas. Reproduced with permission from ref (268). Copyright 2017 Wiley-VCH.

A myriad of reports show that pristine Pt-based sensors exhibit intriguing H_2_ sensing properties. In the H_2_ ambient, prechemisorbed oxygen atoms on Pt are displaced with H_2_ to form a unique hydrogen-terminated (Pt–H) surface.^261,294,295^ Reversibly, oxygen atoms are rechemisorbed upon exposure of the Pt–H surface to air. Typically, electron scattering cross-section at the Pt–H surface is smaller than for the oxygen-terminated (Pt–O) surface in air, thus a displacement of preadsorbed oxygen with H atoms increases the conductivity of Pt (Figure 15a,b). Although the sensitivity and selectivity toward H_2_ are generally not as high as observed for pristine Pd-based sensors, surface modification can be applied to realize extraordinarily high sensitivity and selectivity toward small H_2_ concentrations. To this end, Ding et al.^268^ demonstrated ultrahigh sensitivity and selectivity of ultrathin (≈3 nm) Pt NWs toward H_2_. Typically, reducing the dimension of the metal to sizes below the mean free path of electrons significantly affects the resistance upon interaction with adsorbates.^296^ This property is completely different from that of macroscopic wires, whose resistivity relates linearly to the cross-sectional area and inversely to the length, and are generally insensitive to chemisorption. The ultrathin Pt NWs (Figure 15c) indicated minimal charge transport barriers, and low sheet resistance (≈500 Ω·sq^–1^) upon drop casting on prefabricated gold electrodes. Adsorbed H_2_ on Pt NWs acted as diffusive scattering centers for electrons, resulting in a large change in conductivity. Figure 15d indicates a linear increase in response of Pt NWs with H_2_ concentration in the range of 1–50 ppm. In fact, a relatively high response (Δ*G*/*G*%) of 261.4 ± 46.5% was achieved toward 0.5% H_2_. To improve the selectivity of Pt NWs, their surfaces were modified by anchoring ligands such as 1-butanethiol, 1-octanethiol, 1-hexadecanethiol, 1-butylamine, 1-octylamine, and oleylamine to create hydrophobic overlayers, which altered the dynamics of H_2_ diffusion. Despite the decrease in response of bare Pt NWs when overlaid with thiol-terminated alkanes, their moisture (20% RH) discrimination was higher than that observed for amine-terminated ligands (Figure 15e). On the other hand, the performance of Pt NWs overlaid with amine-terminated ligands outweighed that of bare and thiol-coated Pt NWs, reaching as high as 768.0 ± 108.6% for 1-octylamine coated Pt NWs. The differences in response of amine- and thiol-coated Pt NWs toward humidity and H_2_ can be explained by the fact that thiols bond with metal surfaces via strong covalent bonding,^297^ creating more densely packed separation overlayers. Unlike thiols, amines interact via weak covalent bonding, wherein shorter molecular lengths provide a more uniform coating than longer ones.

#### 4.1.3 Silicon-Based Chemiresistors

Micro-/mesoporous crystalline silicon possesses higher surface activity than bulk silicon, which makes it suitable as an active material in gas sensors.^298,299^ Controllable fabrication of porous silicon provides a network of nanometer-sized structures with a large internal surface area in a small volume (≈500 m^2^·cm^–3^) that can allow massive gas adsorption at room temperature. Importantly, gas-stimulated modification of silicon surfaces leads to a significant change in the electrical conductivity when exposed to gaseous chemicals.^299−301^ Peng et al. have attained selective, fast-responding, highly reversible, subppm of NO-sensitive porous silicon nanowires via a metal-assisted chemical etching method.^299^ On the other hand, Mirzaei et al.^302^ investigated the performance of vertically aligned nanowires for H_2_ sensing, which occurs on silicon via the extraction of the holes from silicon to form ionized hydrogen, causing an increase in the resistance. Enhanced response was attributed to chemical reactivity and smaller bond energy of H_2_, as well as strength of interaction with silicon. The response to H_2_ was just about twice that of ethanol and three times that of H_2_S in the concentration range of 10–50 ppm at 100 °C. Also, it was found that the adsorption kinetics of H_2_ onto silicon was relatively slow due to slow surface reactions. Moreover, a study by Field et al.^303^ has reported irreversible adsorption upon exposure of aligned Si NWs to both NH_3_ and NO_2_. Intrinsically p-type Si NWs exhibit increase and decrease of the resistance upon exposure to electron-donating NH_3_ and electron-withdrawing NO_2_, respectively.

Practically, the reactivity of single-crystalline silicon toward specific gases is relatively low, unless chemical-sensitive layers are incorporated on surfaces. To this end, Shehada et al.^304^ investigated discrimination of various gastric cancer linked VOCs (6-methyl-5-hepten-2-one, 2-propenenitrile, furfuraldehyde, 2-ethyl-1-hexanol, and nonanal) using modified silicon nanowires. Si NWs were subjected to various molecular modifications, including trichloro-(phenethyl)silane (TPS), to create independent and cross-reactive gas sensory responses. Modification with trichloro-(phenethyl) silane (TPS) resulted in a sensitive and selective sensor toward 6-methyl-5-hepten-2-one, with 25% of real breath samples, indicating 71% sensitivity, 89% specificity, and 85% accuracy. The high specificity and accuracy values could make the breath test reliable and useful.

### 4.2 Metal Alloy/Composite-Based Chemiresistors

**Figure 16:**
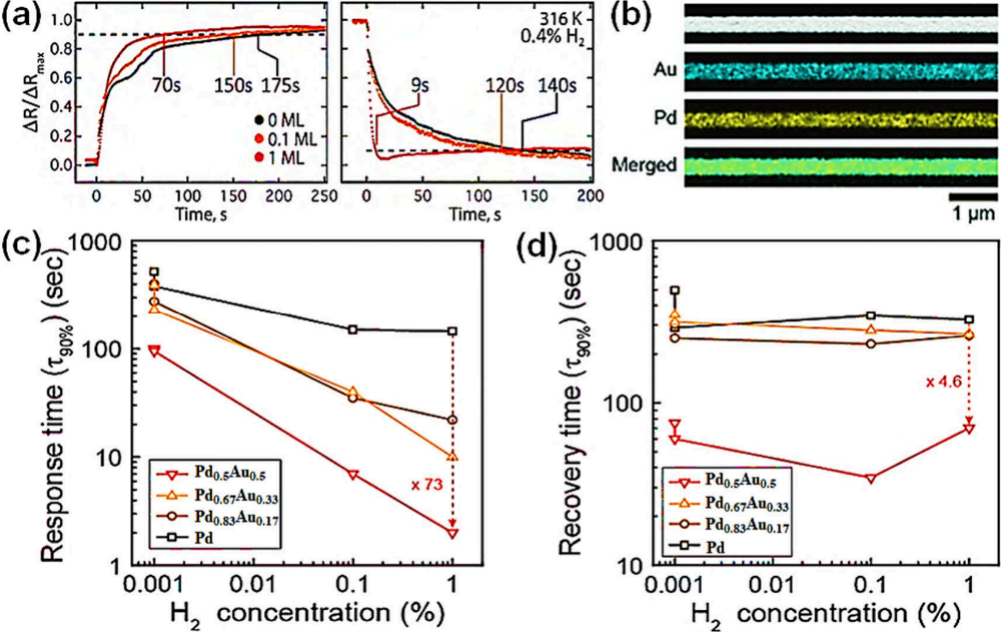
(a) Normalized resistance versus time plots for the response (left) and recovery (right) of a Pd nanowire and two Pd@Pt nanowires showing response and recovery times. Shown are data for Pt = 0 ML (pure Pd), 0.1 ML, and 1.0 ML as indicated. *T* = 316 K, and the hydrogen concentration is 0.4% in air. Reproduced with permission from ref (202). Copyright 2015 American Chemical Society. (b) EDS images of PdAu nanopatterns, (c) response and (d) recovery times of sensors (Pd, Pd_0.83_Au_0.17_, Pd_0.67_Au_0.33_, and Pd_0.5_Au_0.5_) for 0.001–1% H_2_. Reproduced with permission from ref (305). Copyright 2018 Wiley-VCH.

Several studies suggest that metal alloys/composites provide improved sensing performance compared to pure metals and nonmetals. Li et al.^202^ have reported that enhanced response and recovery speeds can be achieved by electrodeposition of a conformal ultrathin Pt overlayer on LPNE-grown Pd NWs. Under optimized coverage of Pt overlayer (1.0 monolayer (ML)), the response and recovery speeds of Pd NWs were accelerated because of synergetic functionalities (Figure 16a). In particular, the response and recovery times generally decreased after a slight increase of temperature to 376 K; however, there was no significant variation in response in this temperature range (i.e., 294–376 K). When a thicker Pt overlayer (10 ML) was deposited, the sensor became almost insensitive to H_2_, whereas layers thinner than 1.0 ML had no influence on H_2_ response. These composite nanostructures represent promising combinations for enhancing the response and recovery times of Pd nanostructures. However, high performance composites or multimetallic nanostructures of various compositions with intricate morphologies are difficult to achieve.

Recently, Jung et al.^305^ introduced a generic approach involving plasma ion bombardment (using low-energy Ar^+^) and reactive ion etching to fabricate ordered multielemental linear nanopatterns for H_2_ sensing. With this method, freestanding nanosheets with smaller thicknesses can be produced. To obtain the nanosheets, layers of target metal films were sequentially deposited onto rectangular patterned polystyrene (PS) using e-beam evaporation technique. Afterward, the layers were subjected to Ar^+^ ion bombardment, resulting in etching and resputtering of metal particles on the sidewalls of the PS patterns. Exposure of as-obtained patterns to oxygen plasma removed the PS, generating freestanding high-aspect-ratio ultrathin (10 nm thick) nanosheets with uniformly distributed metallic components (Figure 16b). Because of these features, Pd_0.5_Au_0.5_ nanosheets revealed an ultrafast response (2 s) and a recovery (70 s) toward 1% of H_2_ (Figure 16c,d). These nanostructures exhibit sensitivity to concentrations as low as 1 ppm of H_2_. For the case of PdPt nanosheets, the Pd_0.67_Pt_0.33_ showed a response (Δ*R*/*R*_a_) > 1 toward 0.001% H_2_, which is higher than those of pure Pd-based sensors. Typically, in a system of bimetallic sensing materials such as PdPt, numerous adsorption sites would be created at Pt–Pt interfaces as the amount of Pt increases.

**Figure 17:**
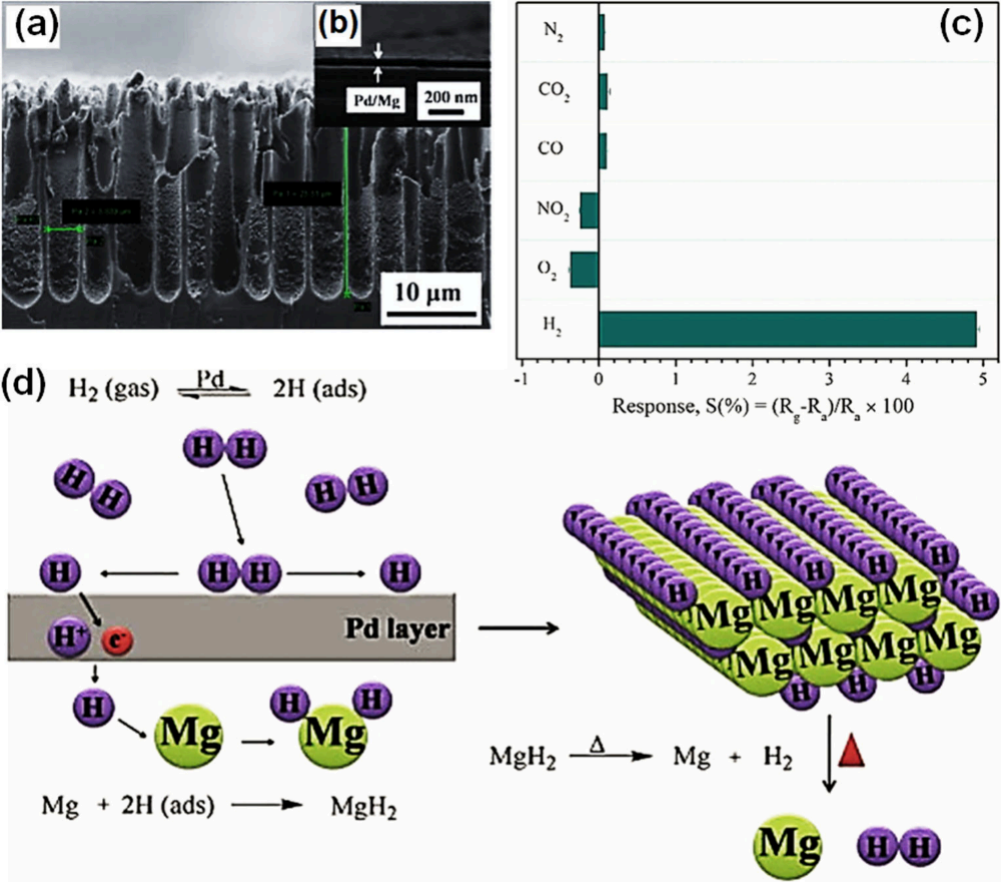
(a) Magnified cross-sectional view of Pd/Mg coated porous silicon. The inset shown as (b) is the magnified view for the cross-section of Pd/Mg thin film. (c) Selectivity histogram of the Pd/Mg nanowires mesh with various test gases, (d) schematic view of the reaction mechanism for hydrogenation and dehydrogenation of Pd/Mg thin film. (a,b,d) Reproduced with permission from ref (308). Copyright 2015 Elsevier. (c) Reprroduced with permission from ref (309). Copyright 2017 Elsevier.

Several research groups have reported that doping Pd with metals such as magnesium (Mg)^267,270,306^ and yttrium (Y)^307^ could be an effective strategy to increase hydrogen uptake. For Mg–Pd alloys, the simultaneous occurrence of two events is expected: First, exposure of Pd to H_2_ shifts its Fermi level, and second, Pd acts as a catalyst to facilitate hydrogenation of the Mg–Pd phase through the formation of MgH_2_. Although a larger resistance increase is achieved when H_2_ is adsorbed on Mg–Pd sensing layers, as compared to pure Pd layers, the recovery characteristics of Mg–Pd are rather poor as a result of high thermodynamic stability of MgH_2_ that shows slow dehydrogenation process, which makes it necessary to increase the operating temperature to above room temperature.^267^ In addition, cyclic exposure of these sensors to H_2_ results in undesirable decay of sensing performance, limiting their uses to low-concentration sensors such as in leak detectors.^306^ To circumvent these deleterious effects, controlling the amount of Mg in Mg–Pd systems and the degree of purity have been reported to play an important role. In addition, reproducibility of measurements (up to several months) can be obtained if the degree of purity and hydrophobicity of the sensing materials is increased.^267^ To this end, Sanger et al.^308^ reported a fast and reversible Pd/Mg based sensor modified by a hydrophobic Si substrate. A nearly 90% porous Si substrate was prepared via electrochemical anodization of p-type Si wafer followed by a room temperature deposition of Pd/Mg film via a direct current (DC) magnetron sputtering system. The cross-sectional view of the sputtered film is shown in Figure 17a and the inset thereof (Figure 17b). As shown in Figure 17c, Pd/Mg NWs are highly selective toward H_2_, while having much weaker responses to interfering gases such as N_2_, CO_2_, CO, NO_2_, and O_2_. Interestingly, the exposure of Pd/Mg NWs to low pressure hydrogen (0.5 bar) did not instigate the formation of the PdH_*x*_ phase. Instead, orthorhombic γ-MgH_2_ was formed, the mechanism of which was attributed to catalytic dissociation of H_2_ onto Pd, and dissolution of atomic H into Mg, forming high resistivity MgH_2_ (Figure 17d). During dehydrogenation, a decrease in resistivity was achieved by the removal of hydrogen atoms, which acted as electron scattering centers. The recovery of Pd/Mg sensors became about 13 times faster upon increasing the operation temperature to above room temperature (RT). This observation is similar to that of Hassan et al., who observed a decrease in recovery time from 33 to 15.3 s when the temperature was elevated from RT to 75 °C during dehydrogenation of 10,000 ppm of H_2_.^267^ The presence of hydrophobic porous Si minimized the moisture poisoning, where the porosity induced the formation of higher charging sites for easy adsorption and desorption of H_2_ on the sensing film. A separate investigation indicated that quantum size (domain size of 6 nm) of Pd/Mg NPs endows Pd/Mg NWs with more highly charged sites and increased hydrophobicity, resulting in enhanced H_2_ sensing.^309^

Other combinations demonstrating synergistic functionalities among metals and nonmetals involve metal overlayers or nanoparticles on silicon surfaces. Several authors have implemented surface modifications of pristine silicon by incorporating ultrathin, active sensing layers such as metallic Pd overlayers,^272,310,311^ NPs,^266,312^ and inorganic functionalities,^304^ enabling gas-specific detection. Gao and co-workers demonstrated that Pd-decorated n^–^-doped silicon (Pd-decorated n-Si) shows fast response rates, high selectivity, low LOD, and can give stable and highly repeatable results, little affected by humidity.^266^ Specifically, the electron-conducting n^–^-Si can be depleted of electrons when decorated with Pd NPs owing to the high work function of Pd despite a native SiO_2_ layer that forms on the surface of n^–^-Si. It follows that the H atoms diffusing into Pd become polarized in the vicinity of the native insulation layer, eliminating the depletion layer and consequently modulating the electrons concentration in n^–^-Si. This materials arrangement resulted in a response of up to 27% toward 0.8% H_2_, and ultrahigh selectivity against NO_2_, toluene, CO, and ethanol, whereas bare Si films remained almost insensitive to H_2_ under the same conditions. Notably, the gas-sensing performance of silicon-modified nanostructures was shown to depend on the type of current carriers in Si. An investigation by Baek et al.^311^ revealed that the response of Pd-coated n-type Si arrays was nearly 23 times higher than that of Pd-coated p-type Si arrays when both were exposed to 1% H_2_ under the same conditions. The Pd on both the n-type Si and the p-type Si NWs interact with H_2_ to form PdH_*x*_ but each of the Pd coated on n-type Si NWs and Pd coated on p-type Si NWs exhibit opposite sensing mechanisms. Formation of PdH_*x*_ on n-type Si lowers the work function of Pd, accompanied by electrons transfer to n-type Si and a decrease in resistivity. On the other hand, for p-type Si, neutralization of hole carriers takes place as electrons transfer from PdH_*x*_, increasing the resistivity. This explains the enhanced response toward H_2_ for Pd-coated n-type Si NWs. In addition, reduction in the work function of Pd is due to the formation of PdH_*x*_, which indicates that these sensors can be highly selective toward H_2_.^311^

### 4.3 Challenges in Metal-Based Chemiresistive Sensors

Despite efforts made to explore metal-based chemiresistive sensors, several challenges remain unsolved. For example, while Pd NWs can offer fast response and recovery speeds, their response is limited to a narrow range of resistivity variation (typically, 1–10%) upon exposure to H_2_.^277^ This is because the dissolution of H_2_ in Pd to form PdH_*x*_ is limited, and a plateau in resistivity is reached at *x* ≈ 0.7. In addition, the two sensing mechanisms of Pd toward H_2_ can coexist and negate the effect of each other, unless one of the mechanisms is extremely predominant.^313^ As observed, H_2_ uptake of Pd, and hence response, can be increased by combining Pd with other elements such as magnesium and yttrium. However, the proportions of these elements in Pd are limited to diminutive amounts to avoid high resistivity and high temperature sensor operation. In addition, because of the slow hydrogenation and dehydrogenation processes involved, their response and recovery times are somewhat poorer than those of pristine Pd at room temperature. Likewise, Pd–Pt sensors generally exhibit slow recovery. Another issue is that except few findings such as those reported elsewhere,^271,272,276^ metals and metal alloys have been exclusively explored for H_2_ sensing. This is because most metals and metal-based alloys are not chemisorptive to a wide variety of gases. There remains therefore a room to explore alternative metal-based materials for chemiresistive sensing of various gases.

## 5 Selectivity in Conjugated Polymer-Based Chemiresistive Sensors

### 5.1 Pure Conjugated Polymers

Conjugated polymers (CPs) with sp^2^ hybridized π-conjugated electronic structures interact with gas molecules through direct charge transfer or analyte–receptor interactions, resulting in changes in their conductivity. Upon interaction with oxidizing or reducing gas analytes, these gases can act as electron acceptors or donors, similar to doping effects, which modulate the carrier concentration and resistance in CPs.^314^ Additionally, conductivity modulation can occur through swelling mechanisms,^314^ where specific gas molecules absorb onto CPs chains, causing chain separation. This increases the hopping distance of charge carriers, thereby reducing electrical conductivity. CPs are typically wide-bandgap semiconductors in their pristine state, exhibiting semiconducting or insulating properties with low electrical conductivity. To address these limitations and enhance the sensing performance of CPs, various strategies have been explored. Modulating the gate voltage can effectively tune the charge carrier concentration within CPs-based active layers, leading to an enhanced sensor response. Doping, another promising strategy, modulates the charge density of pristine CPs, improving the conductivity of insulating CPs to achieve either n-type or p-type semiconducting behaviors or even a highly conductive state.^315^ This is attributed to the reduction or oxidation of the π-conjugated systems of CPs.^316^ For instance, redox or protonic acid doping process can remove electrons from the CPs backbones, increasing the major carrier densities in p-type CPs and thereby enhancing their conductivity.

Pure CPs often exhibit low electrical conductivity and a high affinity for various gas analytes, leading to low response and poor selectivity. Despite extensive research over the past decades to utilize CPs in room-temperature gas sensors, these limitations have hindered their practical application in gas sensing. Commonly studied pure CPs include polyaniline (PANI),^317^ polypyrrole (PPy),^318^ polythiophene (PT),^319^ and poly(3,4-ethylenedioxythiophene) (PEDOT).^320,321^ Gas selectivity in pure CPs depends on several intrinsic factors such as molecular architecture, chain conformation, and chemical composition. Structural features including the polymer backbone, side chains, and substituents play pivotal roles in determining the sensor’s interaction with gas molecules. Tailoring these parameters allows for precise modulation of gas selectivity based on factors such as molecular size, shape, polarity, and chemical reactivity. Additionally, the polarity and active sites provided by the polymer side chains significantly impact gas selectivity. Engineering the side chains of conjugated polymers is critical for optimizing gas sensing properties, particularly selectivity.^322^ Side chains significantly influence molecular packing, interchain interactions, and binding affinity with target gas molecules. Functional groups such as carboxylic acids, thymine, and ethyl esters have demonstrated their ability to promote specific interactions, such as hydrogen bonding, salt formation, or coordination with metal ions, enabling selectivity toward gases like ammonia, nitrogen dioxide, and carbon monoxide.^323−326^ To enhance the sensing performance of pure CPs, various strategies have been investigated, such as increasing the surface area by introducing low-dimensional structures, particularly electrospun nanofiber structures.^327−329^ One-dimensional nanofiber networks of CPs can significantly enhance the gas sensing performance by increasing the surface area and enhancing diffusion properties compared to conventional dense film-type CP sensors. Moreover, incorporating hybrid systems by combining CPs with carbon, metals, and porous inorganic materials can not only tune the electronic structures of CPs but also provide preferential gas adsorption sites, effectively enhancing gas selectivity. In this section, we will discuss state-of-the-art strategies for improving the gas sensing properties of CPs, with a particular focus on achieving high gas selectivity.

### 5.2 Doping and Functionalization in Conjugated Polymers

**Figure 18:**
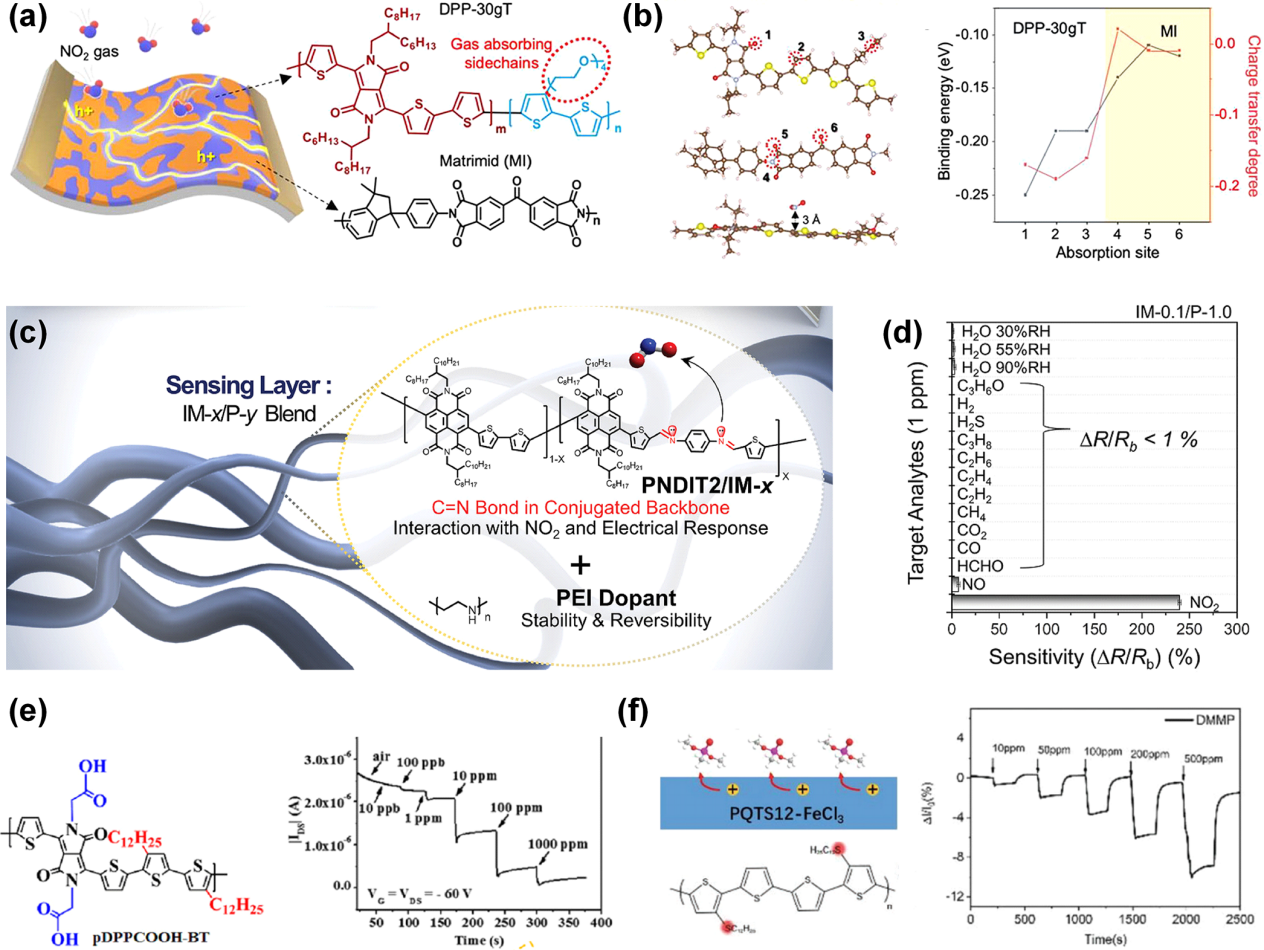
(a) Schematic illustrations of a gas sensor based on semiconducting polymer composites with chemical structures of DPP-30gT and Matrimid. (b) NO_2_ adsorption sites and respective calculated binding energies with DPP-30gT–Matrimid composites. Reproduced with permission from ref (335). Copyright 2023 American Chemical Society. (c) Schematic illustrations of imine incorporated PNDIT2-based sensors with PEI dopants. (d) Selective gas sensing properties toward 14 different gas analytes. Reproduced with permission from ref (336). Copyright 2022 Wiley-VCH. (e) Structures and NH_3_ sensing graphs of pDPPCOOH-BT-based sensors. Reproduced with permission from ref (337). Copyright 2016 American Chemical Society. (f) Structures and DMMP sensing mechanisms of PQTS12-FeCl_3_-based sensors. Reproduced with permission from ref (338). Copyright 2024 Wiley-VCH.

Heeger et al. first demonstrated that doping polyacetylene (PA) with halogens could lead to tunable electrical conductivity^330,331^ and optical properties. The incorporation of dopant molecules into conjugated polymers with properly controlled dopant types and concentrations could lead to effective modulations of the electrical conductivity of CPs ranging from metallic to semiconducting and even insulating.^332^ Doping or functionalizing conjugated polymers introduces specific chemical moieties or additives that interact selectively with target gas analytes, thereby enhancing gas selectivity.^333,334^ For example, the presence of polar functional groups can enhance the selectivity of the sensor toward polar analytes. Yang et al., reported that the incorporation of gas-adsorbing polar side chains, such as triethylene glycol (TEG), into diketopyrrolopyrrole (DPP)-based organic mixed ionic-electronic conductors increased the binding energy toward polar NO_2_ molecules, thereby increasing the degree of charge transfer as well as the response values (Figure 18a,b).^335^ In particular, triggering a spinodal-like blending morphology of DPP within a high *T*_g_ (glass transition temperature) polyimide matrix enabled sensitive and selective detection of NO_2_ gas even at high operating temperatures (∼170 °C), with excellent thermal stability.

Functional groups appended to the polymer backbone can exhibit preferential affinity toward certain gas species, facilitating specific molecular recognition and binding. Furthermore, dopants or additives can modify the electronic structure of the polymer, leading to changes in charge carrier concentration and conductivity in response to gas adsorption. In this regard, it was found that both functionalization with imine groups (C=N) and doping with poly(ethylenimine) (PEI) could enhance electrical conductivity, interaction with polar NO_2_ gas, sensing stability, and reversibility when compared to the unmodified naphthalenediimide-based conjugated polymers (Figure 18c,d).^336^ Through ex situ X-ray photoelectron spectroscopy and Raman spectroscopy analyses conducted before and after exposure to NO_2_ gas, researchers demonstrated that the high response and selectivity observed were primarily due to the strong interaction of imine (C=N) bonds with NO_2_ analytes, facilitated by the electron-withdrawing nature of oxidative NO_2_ gas.

Other dopants and functional groups can be integrated into conjugated polymers to impart gas selectivity toward different target gases. For instance, n-type boron β-diketone-containing conjugated polymers display highly selective and sensitive NH_3_ sensing capabilities due to the electron-deficient nature of boron β-diketone heterocycles, which effectively modulate the charge distribution of active centers (Figure 18e).^339^ This modulation enhances the adsorption of electron-donating ammonia gas, resulting in the highest response (*R*_a_/*R*_g_ > 1500 @ 40 ppm of NH_3_) and excellent selectivity compared to state-of-the-art conjugated polymer-based sensing materials. In a separate study, doping poly(bisdodecylthioquaterthiophene) (PQTS12) conjugated polymers with FeCl_3_ doping significantly improves electrical conductivity by 5 orders of magnitude.^338^ PQTS12-FeCl_3_-based sensing materials exhibit high selectivity toward dimethyl methylphosphonate (DMMP) gas species, attributed to the strong hydrogen bond between the P=O group on DMMP and the C–H group on the PQTS12 side chain (Figure 18f). These studies underscore the significance of doping and incorporating functional side chains on the polymer backbone to enhance electrical conductivity and induce selective chemical reactions and bonding with specific target gases. Controlled doping concentration within the polymer matrix allows for improved selectivity toward specific gases. For example, introducing electron-withdrawing dopants such as metal halides or organic molecules with strong electron affinity enhances sensitivity and selectivity toward analytes that readily accept electrons. Conversely, electron-donating dopants facilitate interactions with electron-deficient gases. By carefully selecting and optimizing dopants, the sensing performance and selectivity of conjugated polymer-based chemiresistive sensors can be significantly enhanced.

### 5.3 Conjugated Polymer-Based Hybrids

To further enhance selectivity, conjugated polymers can be blended with other materials to form hybrid systems that exhibit synergistic properties. Incorporating heterostructures composed of diverse materials such as carbons, metals, metal oxides, or porous inorganic frameworks into conjugated polymer-based sensors provides a versatile approach for optimizing gas selectivity. These heterostructures serve as complementary components that augment the sensor’s performance by providing additional sites for gas adsorption, diffusion, and catalytic reactions. The synergistic interactions between the conjugated polymer and heterostructured materials facilitate selective gas recognition and promote specific chemisorption or physisorption processes. Moreover, the engineered interfaces between heterostructured materials can modulate charge transfer kinetics, surface reactivity, and gas diffusion pathways, thereby enhancing the sensor’s sensitivity, selectivity, and response dynamics for a wide range of analytes.

#### 5.3.1 Conjugated Polymers-Carbon-Based Hybrids

**Figure 19:**
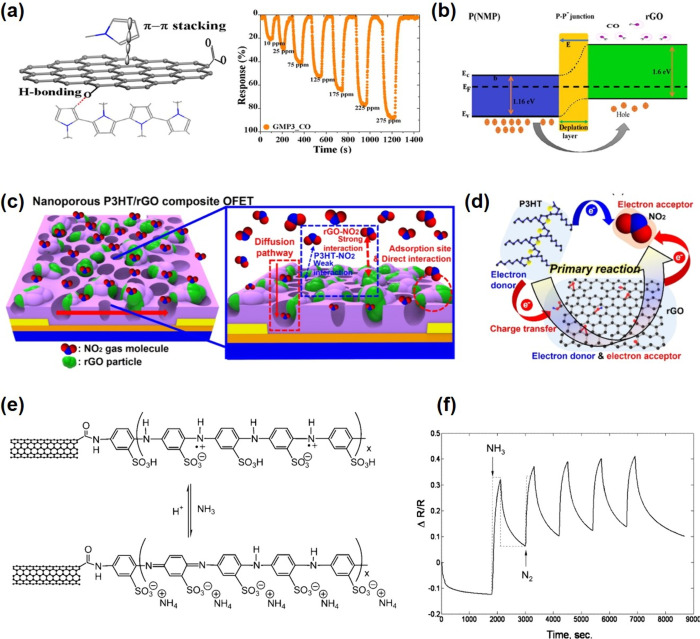
(a) Schematic illustrations of p(NMP) and rGO composites sensors for reversible and selective CO sensors. (b) Energy band structure of p(NMP) and rGO upon CO exposure. Reproduced with permission from ref (340). Copyright 2022 Elsevier. (c) Schematic illustrations of nanoporous P3HT/rGO composite-based OFET sensors for NO_2_ sensing and (d) NO_2_-sensing mechanisms of charge transfer interactions between P3HT, rGO, and NO_2_ analytes. Reproduced with permission from ref (341). Copyright 2023 American Chemical Society. (e) Schematic illustrations of PABS polymer wrapped SWCNT for NH_3_ sensing, and (f) dynamic NH_3_ sensing properties of the composite sensors. Reproduced with permission from ref (342). Copyright 2004 American Chemical Society.

The integration of conjugated polymers with carbon-based materials, such as carbon nanotubes (CNTs), graphene, graphene oxide (GO), and reduced graphene oxide (rGO), has been investigated to enhance gas sensing performance due to their large specific surface area and favorable electrical conductivity.^343,344^ Combining conjugated polymers with CNTs or graphene introduces additional sensing sites, increases the specific surface area, and improves the electrical conductivity of the hybrid systems. This synergistic effect results in enhanced selectivity by facilitating charge transfer and interactions with specific gas molecules. For example, Mohammad et al. demonstrated a highly selective and sensitive CO sensor utilizing poly(*N*-methyl pyrrole) (P(NMP)) and rGO composite systems (Figure 19a).^340^ These composites were synthesized using a self-assembled micellar soft template protocol in the presence of rGO layers. The P(NMP)/rGO composites exhibited excellent CO response (231.5% @75 ppm of CO) with good selectivity, attributed to improved reaction sites and electrical conductivity (Figure 19b). To further optimize the incorporation of carbon-based additives, it is crucial to minimize the amount of additives embedded in the CP-based film. In this context, Shin et al. presented a blending system where rGO was integrated into nanoporous poly(3-hexylthiophene) (P3HT) films using the shear coating-assisted phase separation technique (Figure 19c).^341^ Specifically, nanoporous P3HT film was prepared using a sacrificial templating route with polystyrene beads. The P3HT/rGO blend systems demonstrated high NO_2_ response with excellent selectivity, attributed to the synergistic effects of finely distributed rGO flakes throughout the entire CPs and effective charge transfer from P3HT to rGO (Figure 19d). This interaction with NO_2_ gas involved the donation of electrons from rGO to NO_2_, dramatically modulating the electrical conductivity.

One of the most extensively studied hybrid systems involves CNT/CPs composites, driven by the significant interest in CNT-based electronic devices. Pure CNTs lack sensitivity and selectivity due to the limited gas reaction sites on their surface. Therefore, wrapping CNTs with CPs, either covalently or noncovalently, presents an effective strategy to introduce gas reaction sites with selectivity. For instance, the covalent attachment of poly(*m*-aminobenzenesulfonic acid) (PABS) onto CNTs enables rapid detection of NH_3_ gas analytes (Figure 19e).^342^ Upon exposure to NH_3_ gas, the deprotonation of PABS side chains forms a hole depletion layer, leading to a decrease in the conductivity of the p-type CNT/PABS composites (Figure 19f). Similarly, direct polymerization of aniline in SWCNT results in a 60-fold improvement in NH_3_ response compared to pure SWCNT, with ultralow detection limits (50 ppb) and rapid recovery kinetics.^345^

Noncovalent functionalization of CNTs with CPs also enhances gas sensing performance. For example, CNTs coated with polymeric perfluorinated sulfonic acid ionomer (Nafion) and polyethylenimine (PEI) exhibit selective sensing of NH_3_ and NO_2_, respectively.^346^ In the case of PEI-coated CNTs, the PEI coating alters the CNTs’ semiconducting properties from p-type to n-type, enabling sensitivity to trace amounts (100 ppt) of NO_2_ gas. Meanwhile, Nafion-coated CNTs with p-type semiconducting properties demonstrate good NH_3_ sensing results. Overall, blending CPs with various types of carbon-based materials not only affects the electronic properties of CPs through charge transfer but also enhances gas reaction sites, thereby improving the overall gas sensing performance of pure CPs.

#### 5.3.2 Conjugated Polymers–Metal-Based Hybrids

Doping metal nanoparticles into the conjugated polymers can significantly modulate the electrical properties and gas adsorption/desorption capacities. The resulting metal–polymer hybrids engage in specific chemical interactions with target gases, thereby improving selectivity and response. For example, incorporating metal nanoparticles such as gold or silver induces localized surface plasmon resonance, which facilitates the selective detection of gases through surface-enhanced interactions. Key factors influencing gas sensing performance include the nanoparticles’ size, dispersibility, concentration, and surface coverage. Additionally, the work function difference between the conjugated polymers and metal nanoparticles can provide preferential gas adsorption sites due to energy level alignments.

**Figure 20:**
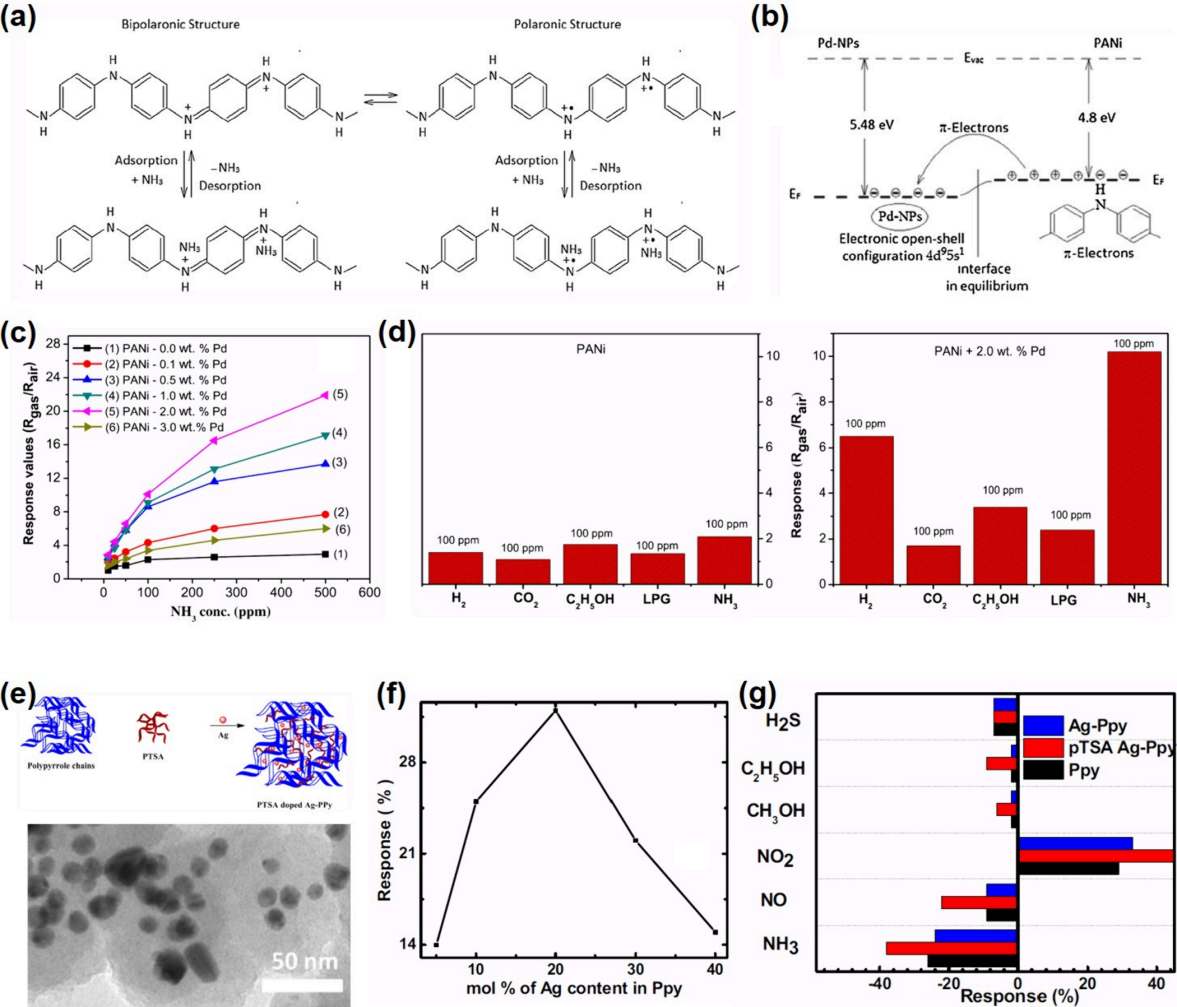
(a) Schematic illustrations of the NH_3_ sensing mechanisms of PANi, and (b) Pd nanoparticles decorated PANi sensors. (c) Effect of Pd content on NH_3_ sensing response of Pd nanoparticles decorated PANi sensors. (d) Selectivity of pristine PANi and Pd nanoparticles decorated PANi composites. Reproduced with permission from ref (347). Copyright 2017 Elsevier. (e) Schematic illustrations of pTSA doped and Ag nanoparticles decorated Ppy composite sensors. (f) Effect of Ag content on NO_2_ sensing response of Ag nanoparticles decorated Ppy sensors. (g) Selectivity of pristine Ppy, Ag-Ppy, and pTSA Ag-Ppy sensors. Reproduced with permission from ref (349). Copyright 2017 Elsevier.

Polyaniline (PANi), a well-known conjugated polymer, can reversibly interact with ammonia gas by adsorbing and desorbing NH_3_ in both bipolar and polaronic forms (Figure 20a).^347^ Protonation of nitrogen atoms in PANi leads to NH_3_ molecules to act as electron donors, resulting in resistance changes. Although PANi is an effective ammonia sensing material, its selectivity and response are limited, and the response/recovery times are usually slow. Enhancing the sensing characteristics by decorating PANi with Pd nanoparticles, which have an open-shell electronic configuration (4d^9^5s^1^), provides effective gas adsorption sites, particularly for the reducing nature of NH_3_ molecules (Figure 20b). Meanwhile, the energy level of Pd is lower than that of the π-electrons in PANi layers, promoting electron transfer from PANi to Pd. This electron transfer generates positive charges or holes on PANi, creating active sites for NH_3_ adsorption. Consequently, the introduction of an appropriate amount of Pd nanoparticles significantly improves NH_3_ sensing response and selectivity (Figure 20c,d). In another work, functionalizing PANi with Ag nanoparticles significantly enhances its ethanol sensing properties. This improvement is attributed to hydrogen bonding between the −OH groups of ethanol and the amine and imine groups in PANi layers.^348^ Fourier transform infrared spectroscopy demonstrated notable structural modifications by increased absorption peak intensities related to the C=N stretching of quinoid and the C=C stretching of benzenoid upon exposure to ethanol. In another work, thin film sensors based on *p*-toluenesulfonic acid (pTSA)-doped polypyrrole (Ppy) decorated with silver nanoparticles showed a significant response to NO_2_ molecules (Figure 20e).^349^ The size and concentration of Ag nanoparticles are crucial for achieving optimal NO_2_ sensing properties (Figure 20f). Compared to pristine Ppy sensors, pTSA-doped Ag-Ppy composites demonstrated greatly enhanced response and selectivity toward NO_2_ molecules (Figure 20g).

To sum up, introducing metal nanoparticles can enhance the sensing properties of conducting polymers for several reasons: first, metal nanoparticles alter the conductivity of the polymers. Second, some metal nanoparticles have a chemical affinity for specific gases due to their electronic configurations. Finally, the effective surface area of the nanocomposites that interact with target gases is increased by incorporating metal nanoparticles into the conjugated polymers. This synergy between metal nanoparticles and conjugated polymers not only boosts sensitivity but also offers tailored solutions for diverse sensing applications.

#### 5.3.3 Conjugated Polymers–Porous Inorganic Materials-Based Hybrids

Combining conjugated polymers with porous inorganic materials, such as zeolites and metal–organic frameworks (MOFs), has emerged as a promising approach to achieve enhanced response and selectivity. The porous structure of these materials provides a large surface area and high porosity, offering enhanced gas adsorption sites and interactions. This unique feature also allows for the selective binding of target gases within the pores of the inorganic materials.

**Figure 21:**
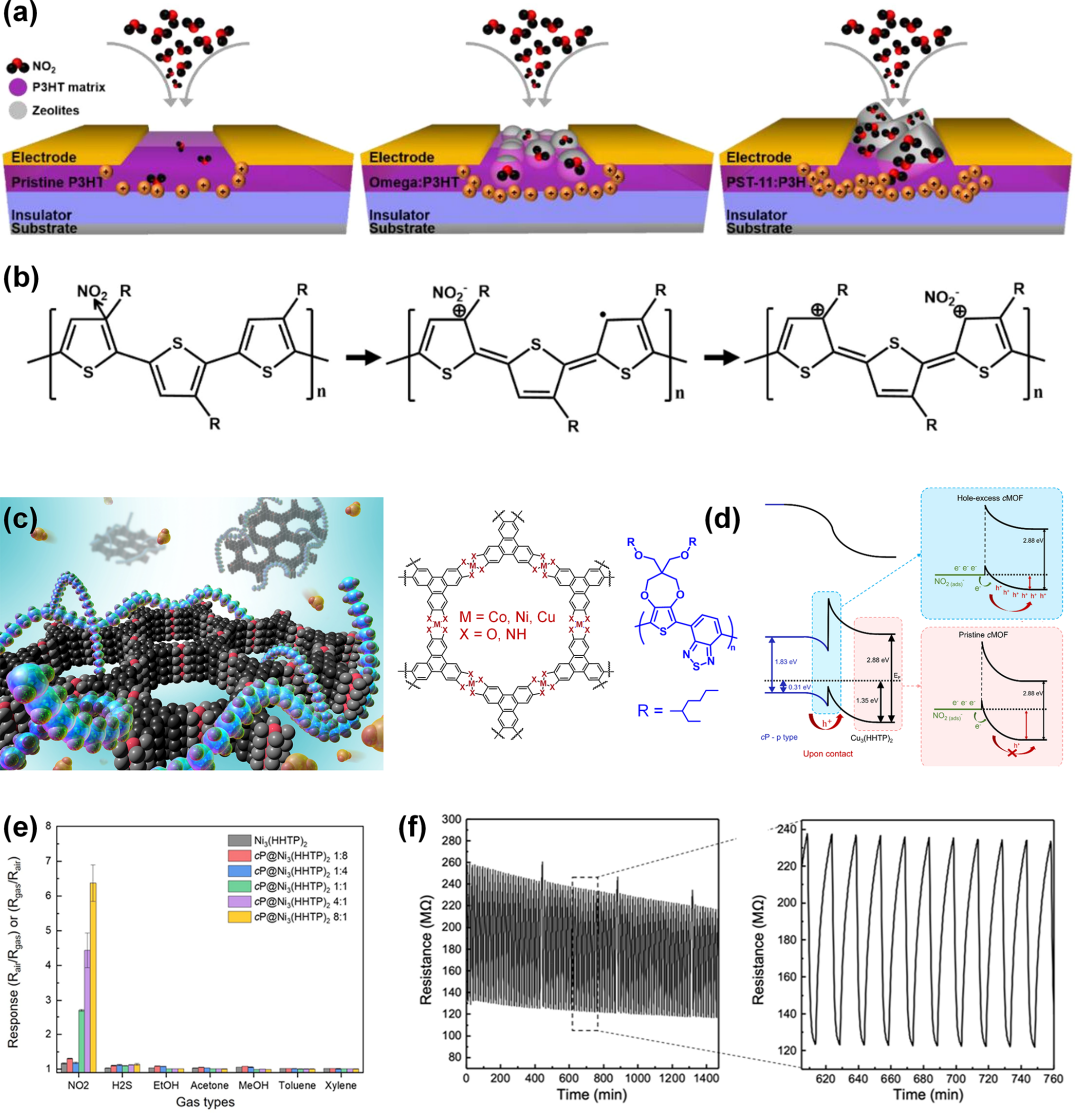
(a) Schematic illustrations of NO_2_ adsorption by pristine P3HT, Omega:P3HT, and PST-11:P3HT. (b) P3HT doping mechanisms by adsorption of NO_2_ gas species. Reproduced with permission from ref (350). Copyright 2021 Elsevier. (c) Schematic illustrations of ProDOT-BTD CP and conducting MOFs composites-based sensors. (d) Energy level diagram of CPs and cMOFs upon contact and proposed mechanism for enhanced recovery upon NO_2_ exposure. (e) Selective gas sensing properties of pristine cMOFs and CP@cMOFs composites. (f) Long-cycling NO_2_ sensing stability of CP@cMOFs sensors. Reproduced with permission from ref (351). Copyright 2024 Wiley-VCH.

Zeolites, with their well-defined microporous structure and affinity for certain gas molecules, can be utilized in conjugated polymer-based hybrids to achieve enhanced selectivity. The conjugated polymer can be coated onto the zeolite surface or infiltrated into the pores, enabling selective interactions with target gases. Kwon et al. reported the fabrication of a novel organic–inorganic hybrid gas sensor by blending zeolite with CPs. They incorporated PST-11 (MEI) and Omega (framework type MAZ)-based zeolites into poly(3-hexylthiophene) (P3HT)-based CPs (Figure 21a).^350^ Zeolites, particularly PST-11 and Omega-based ones with low Si/Al ratios exhibit strong gas adsorption toward hydrophilic molecules. Consequently, NO_2_, the target gas, could effectively be captured within the zeolite pores due to the robust interaction between the cation sites of zeolites and NO_2_ gas molecules. The adsorption of NO_2_ on both zeolites and P3HT polymers induces a p-type doping effect, thereby enhancing electrical conductivity. This effect arises from the oxidative nature of NO_2_ gas, which extracts electrons from the CPs’ backbone, leading to a transformation of the P3HT backbone from a benzoid to a quinoid structure (Figure 21b). With increasing amounts of adsorbed NO_2_ gas, facilitated by the presence of zeolite layers, a greater extent of transformation from benzoid to quinoid structure occurs, consequently improving response and selectivity.

Recently, highly reversible and selective NO_2_ gas sensors were demonstrated by utilizing blends of CPs with electrically conductive metal–organic frameworks (cMOFs) (Figure 21c).^351^ As gas adsorption sites, cMOFs were prepared using 2,3,6,7,10,11-hexahydroxytriphenylene (HHTP) and 2,3,6,7,10,11-hexaiminotripphenylene (HITP) ligands with varied metal nodes (Co, Cu, and Ni) and mixed with p-type CPs consisting of 3,4-propylenedioxythiophene and 2,1,3-benzothiadiazole building units.^352^ In the composite system, both cMOFs and CPs provide abundant gas adsorption sites and electrical conductivity. Especially, p-type CPs actively provide holes to cMOFs upon hybridization based on energy band alignments at the heterojunctions between cMOFs and CPs (Figure 21d). The excess hole density in cMOFs reduces the binding energy between NO_2_ and cMOFs, thereby enhancing the desorption kinetics of NO_2_ gas. This gas is typically strongly chemically adsorbed on the surface of cMOFs, generally resulting in dosimetric sensing behaviors. As a result, the sensors exhibited excellent NO_2_ gas selectivity as well as long-term stability, which has rarely been reported in pristine CPs and cMOFs (Figure 21e,f).

In summary, the integration of conjugated polymers with porous inorganic materials, such as MOFs or zeolites, offers an effective strategy to achieve enhanced selectivity in gas sensing. The synergistic combination of the conjugated polymer’s electrical properties and the selective adsorption capabilities of the porous inorganic material leads to improved gas-sensing performance, opening new possibilities for selective detection of target analytes.

### 5.4 Challenges in Conjugated Polymer-Based Chemiresistive Sensors

Conjugated polymers and their composites hold great promise for high-performance chemiresistive gas sensors due to their unique electronic structures, but they still face several critical challenges. One major limitation is the inherently low electrical conductivity of CPs in their pristine form, which restricts the broad selection of CP materials that are effective as resistive-type sensors. Additionally, achieving high selectivity toward specific gases is challenging due to the broad range of possible interactions with different analytes. To address these issues, strategies such as doping and functionalization are employed to enhance the electrical conductivity of CPs and tailor their reactivities with specific target gases. However, these modifications can introduce complexity and variability in sensor performance.

The integration of CPs with other materials, such as carbon nanomaterials, metals, and porous inorganic frameworks, can improve selectivity and sensitivity by providing additional active sites and facilitating charge transfer upon reactions with gas analytes. Nanopatterning the backbone of CP materials and the in situ growth of thin-layered porous inorganic materials such as MOFs and zeolites as overlayers to construct heterogeneous structures could be promising strategies to further enhance gas sensing properties by effectively constructing heterogeneous contacts and creating sieving membranes to control gas selectivity. The conjugated polymer contributes to the electrical conductivity and signal transduction, while the porous inorganic material acts as a host matrix, enabling selective gas adsorption and confinement. Despite these advantages, these hybrid systems face challenges related to optimizing the hybrid structures, ensuring material compatibility, and maintaining long-term stability and reproducibility. To develop more consistent and reliable sensors, it is essential to create advanced hybrid materials with finely tuned properties, explore novel doping agents and functional groups, and implement elaborate fabrication techniques.

Moreover, achieving long-term stability remains a key challenge due to issues like polymer aging and thermal degradation. Elevated temperatures can destabilize polymer backbones, reducing sensing performance over time. Recent advancements, such as incorporating polar side chains into thermally stable matrix polymers to create spinodal-like morphologies, have improved stability and gas selectivity under high-temperature conditions.^353^ Additionally, single-component polymeric mixed ionic-electronic conductors with ionic pendant groups have shown promise for enhancing thermal stability, reducing water absorption, and ensuring robust performance in harsh environments.^354^ Future research should focus on developing conjugated polymers with enhanced thermal resilience, cycling durability, and compatibility with environmentally friendly processing methods to further improve the stability and selectivity of gas sensors. This could be achieved through a deeper understanding and precise engineering of side chains, as well as the elaborate design of conjugated polymer blends with complementary electronic, thermal, and mechanical properties. Addressing these challenges will be crucial for the practical application of CPs-based chemiresistors in real-world gas sensing scenarios.

Bioinspired polymers offer innovative strategies for enhancing gas selectivity through specific chemical interactions, protein-based templates for dispersing catalytic nanoparticles on metal oxide nanostructures, and other advanced applications. For example, polydopamine (PDA), synthesized through dopamine self-polymerization, mimics the adhesive properties of natural mussel proteins and features functional groups such as amine, imine, and catechol, enabling strong chemical interactions with gas analytes. Its facile polymerization and functionalization make it highly adaptable for integration with various materials, such as metal oxide nanostructures, significantly enhancing gas sensing performance.^355^ For instance, PDA functionalized on metal oxides has been shown to improve formaldehyde detection through specific chemical bonding with target analytes. Beyond PDA, bioinspired conducting polymers like PEDOT:PSS in organic electrochemical transistors (OECTs) have demonstrated exceptional sensitivity and selectivity for gas analytes like H_2_S by leveraging tunable ion-electron interactions.^356^ Furthermore, frameworks such as apoferritin-based hollow protein cages provide efficient solutions for uniform nanoscale catalyst dispersion, further enhancing the selectivity of metal oxide-based gas sensors.^357^ By integrating bioinspired polymers with advanced nanostructures, future gas sensing technologies can overcome challenges related to sensitivity, selectivity, and multifunctionality, paving the way for robust and reliable systems capable of detecting a wide range of analytes under real-world conditions.

## 6 Selectivity in 2D Materials-Based Chemiresistive Sensors

The demand for advanced nanoscale sensing materials that are highly sensitive, selective, and fast responding/recovering has led to intensive investigations on the use of 2D materials, in particular, graphene, graphene derivatives, and transition metal dichalcogenides as sensing layers.^127^ Ideally, the intriguing features of 2D materials such as exceptional charge transport, atomic scale thickness, and large specific surface area endow them with abundant reaction sites, an important prerequisite for gas absorption. In this section, recent optimized strategies for improvement in selectivity of 2D materials toward various gases are elaborated, with greater emphasis on material design, surface modification and functionalization, and defect engineering.

### 6.1 Transition Metal Dichalcogenides (TMDs)

Recently, 2D TMDs have emerged as a new class of materials due to their large surface-to-volume ratio, high reactivity, tunable electrical properties, and operation at low temperature.^358,359^ Several studies have shown that the sensing mechanisms for TMD-based sensors highly rely on the charge transfer between adsorbed gas molecules and TMDs. For instance, Li et al. first reported MoS_2_ field-effect-transistor (FET)-based chemical sensors.^360^ Single- or few-layered MoS_2_ films showed high response to NO at room temperature with a low detection limit (0.3 ppm). After this pioneering work, various TMDs such as MoS_2_,^361,362^ WS_2_,^363,364^ SnS_2_,^136,365^ and MoSe_2_^366^ have been studied for their applications as chemical sensing layers. It should be noted that most TMDs show high reactivity to polar gas molecules such as NO_*x*_, NH_3_, and H_2_O due to their high reactivity even at low temperature.^367^ In this section, we discuss the strategies to achieve improved selectivity for TMD-based chemiresistors, which include (1) rational design of TMD-based nanostructures, (2) surface modification of TMDs by light irradiation, and (3) functionalization of TMDs with catalysts or selectors.

#### 6.1.1 Design of TMD-Based Nanostructures

**Figure 22:**
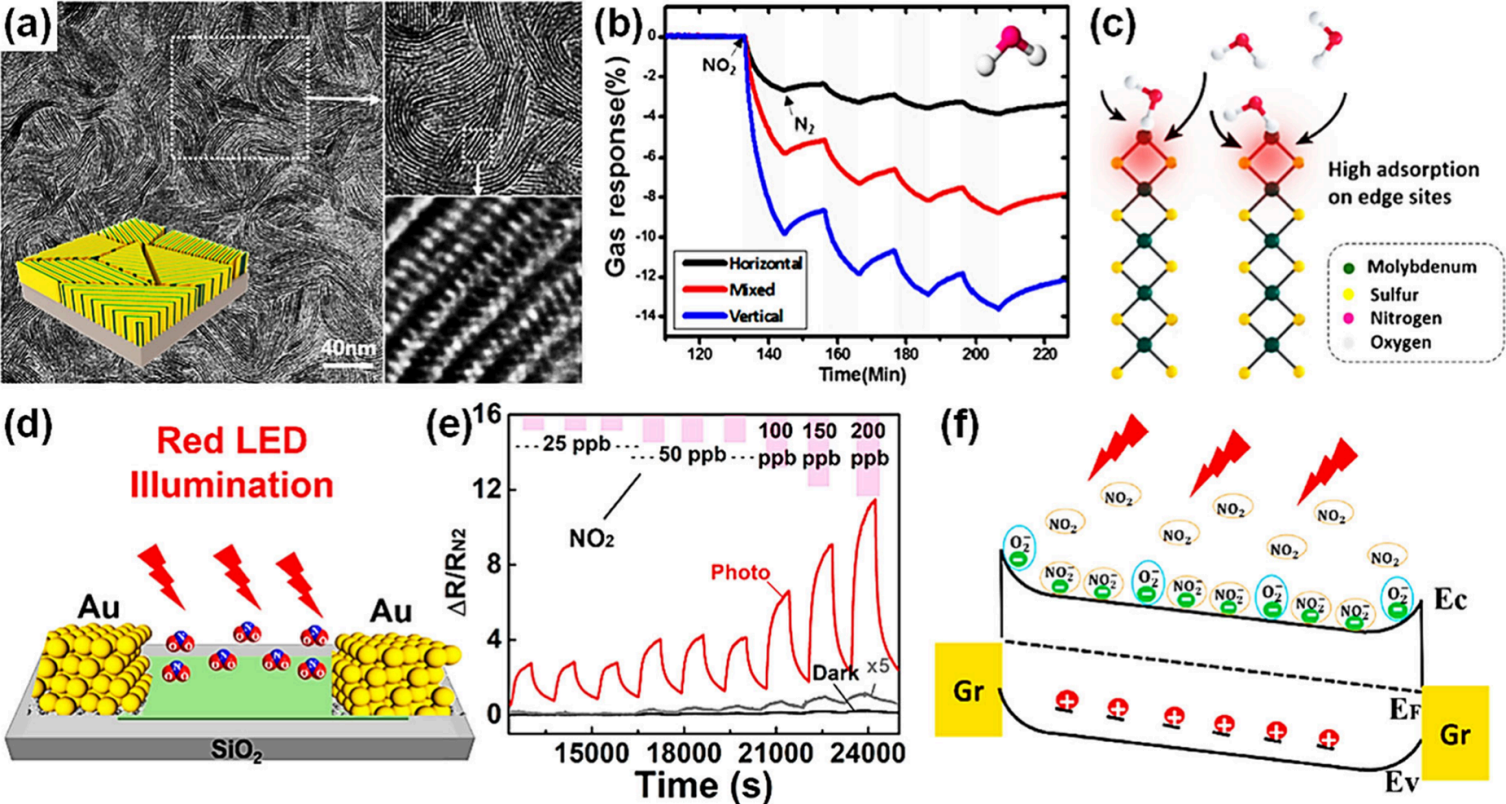
(a) TEM characterization of vertically aligned MoS_2_ layers synthesized by using sulfurization method of CVD process. (b) Gas response traces of MoS_2_ layers with different alignment upon exposure to NO_2_ 100 ppm. (c) Schematic illustration of NO_2_ adsorption on the edges of MoS_2_. Reproduced with permission from ref (368). Copyright 2015 American Chemical Society. (d) Schematic illustration of MoS_2_-based sensors with red LED illumination. (e) Response traces of MoS_2_ to NO_2_ exposure under red light illumination. (f) Band diagram of the devices and NO_2_ reactions on MoS_2_ under red light. Reproduced with permission from ref (372). Copyright 2019 American Chemical Society.

One of the most representative techniques for selectivity enhancement in TMDs is to implement a rational design for engineering TMD-based nanostructures. Nanostructures having a high surface area to volume ratio can effectively promote surface reactions on TMDs, resulting in high selectivity toward polar gas molecules (NO_*x*_, NH_3_, or H_2_O). The selectivity of TMDs is often governed by the binding energy between the opened adsorbed sites of edge atoms on TMDs and polar molecules, which can be calculated using density functional theory (DFT).^33^ For example, it has been reported that ultrathin structures of TMD-based sensors significantly improve their gas sensing properties.^360^ Single-layered or few-layered TMDs are highly sensitive to surface gas adsorption based on the charge transfer mechanism, whereas their stability is inferior compared to thicker TMDs.^362,366,368−370^ To achieve high NO_2_ response with high stability, Cho et al.^368^ synthesized vertically aligned MoS_2_ nanosheets-based chemical sensors. The alignment of MoS_2_ nanosheets was controlled by modulating the thickness of predeposited Mo seed layers. After rapid sulfurization using chemical vapor deposition (CVD), the Mo seed layers were converted to MoS_2_ layers with horizontal, mixed, or vertical alignments. The surface of vertically aligned MoS_2_ showed a number of edge sites (Figure 22a), while that of horizontally aligned MoS_2_ consisted of MoS_2_ basal planes. As shown in Figure 22b, MoS_2_ with a vertical alignment showed 5-fold higher NO_2_ response at room temperature than that of MoS_2_ with a horizontal alignment. The edge sites of MoS_2_ exhibit higher reactivity than the basal planes due to their unsaturated coordination bonding.^371^ Therefore, NO_2_ adsorption was promoted at the edges of MoS_2_ (Figure 22c), leading to improved sensing properties. Even though vertically aligned MoS_2_-based sensors exhibit significantly improved gas response, their reversibility remains suboptimal, likely due to the strong chemisorption of NO_2_ gas at the edge sites of MoS_2_.

Hence, to utilize the high reactivity of edge sites in TMDs, confinement of TMD nanoplates in carbon materials was developed to generate edge-abundant TMD-based nanostructures.^363,364^ For instance, Koo et al.^364^ demonstrated improved NO_2_ sensing properties by forming edge-abundant WS_2_ nanoplates confined in MOF-templated carbon composites. The WS_2_ precursor (ammonium tetrathiotungstate, [NH_4_]_2_WS_4_) was infiltrated into the cavities (∼11.6 Å) of Co-based zeolite imidazole framework (ZIF-67) by electrostatic interactions between the WS_2_ precursors (WS_4_^2–^) and open metal sites (Co^δ+^) in ZIF-67. The subsequent pyrolysis produced few-layered WS_2_ nanoplates-functionalized carbon composites because the growth of WS_2_ was suppressed by the cavity of ZIF-67. The edge-abundant WS_2_-loaded carbon composites exhibited higher NO_2_ response (Δ*R*/*R*_g_ = 48.2% upon exposure to 5 ppm of NO_2_) at room temperature in air as compared to bulk WS_2_ nanosheets (Δ*R*/*R*_g_ = 14.3%). Also, the NO_2_ response is about 8-fold higher than that of interfering molecules including hydrogen sulfide, ammonia, acetone, ethanol, and toluene. These studies demonstrated that the design of ultrathin TMDs with numerous open edge sites significantly improves NO_2_ sensing properties of TMDs.

#### 6.1.2 Surface Modification of TMDs by Light Irradiation

Interestingly, irradiating TMD-based sensors with light has been shown to improve their sensing characteristics.^358^ Photons with adequate energies can release the oxygen species adsorbed on active sites of TMDs, which renders these sites available for target gas adsorption. To this end, Kuman and co-workers recently demonstrated UV-activated MoS_2_-based sensors with fast and reversible NO_2_ detection.^373^ The NO_2_ response (ΔR/R) of MoS_2_ flakes was improved from 28% (100 ppm of NO_2_) to 35% under UV-light illumination (1.2 mW/cm^2^) at room temperature. Moreover, it followed that these sensors exhibited improved NO_2_ selectivity against interfering gases (NH_3_, H_2_, H_2_S, CO_2_, and CH_4_). The response time of MoS_2_-based sensors at 100 ppm of NO_2_ was also reduced from 249 to 28 s with UV irradiation. In addition, Pham et al. reported MoS_2_-based optoelectronic gas sensors prepared under red light illumination (Figure 22d).^372^ Since the photon energy of red light matches the band gap of single-layered MoS_2_, the channel resistance of MoS_2_-based sensors decreased upon red light irradiation. As a result, the response of irradiated MoS_2_ upon exposure to 25–200 ppb of NO_2_ were dramatically higher compared to those of MoS_2_ without red light exposure (*S* = 4.9%/ppb, Figure 22e). When the sensors were operated in the dark (without light illumination), adsorbed oxygen molecules at the surface of MoS_2_ would block the interaction of NO_2_ with MoS_2_. However, with light illumination, adsorbed oxygen molecules were eliminated from the surface of MoS_2_, upon which the red-light-induced increase in the electron density further promoted the adsorption of NO_2_ molecules (electron acceptors) (Figure 22f). As a result, the MoS_2_-based optoelectronic gas sensors detected ppb-levels of NO_2_ molecules with high response, low theoretical LODs (0.1 ppb), and fast recovery.

#### 6.1.3 Functionalization of TMDs with Catalysts or Selectors

**Figure 23:**
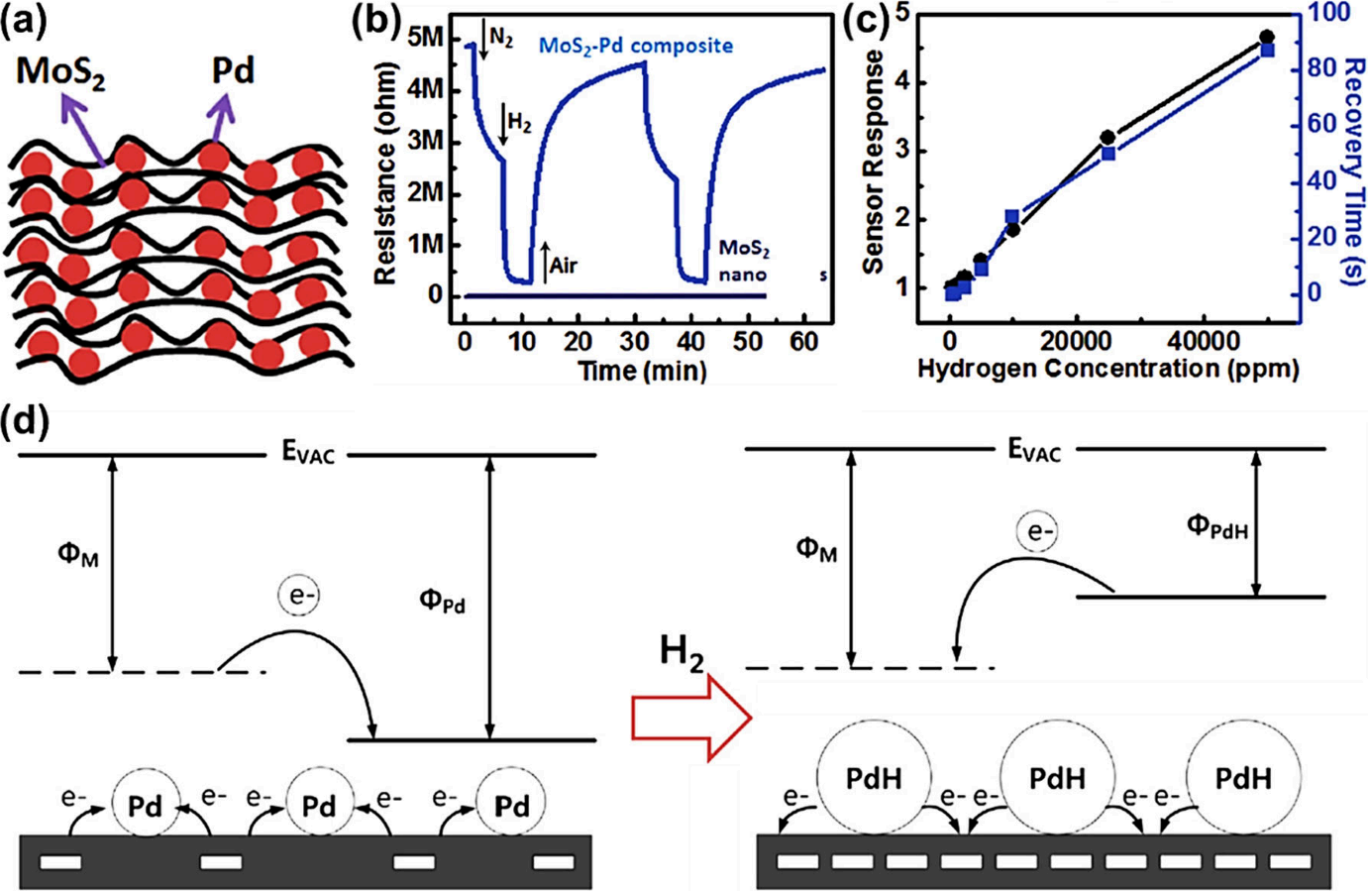
(a) Schematic illustration of the structure of Pd-decorated MoS_2_. (b) Resistance changes of Pd-decorated MoS_2_ upon H_2_ (5%) exposures and (c) their normalized response to H_2_. Reproduced with permission from ref (376). Copyright 2015 Wiley-VCH. (d) Band diagram and schematic illustration of Pd-decorated MoS_2_ before and after H_2_ exposures. Reproduced with permission from ref (379). Copyright 2017 Elsevier.

While TMDs have great potential as high-performance sensors due to their high chemical activity, most TMD-based sensors are limited by their low reactivity to gas molecules other than some polar molecules (NO_*x*_, NH_3_, and H_2_O).^374^ To realize the detection of diverse gas molecules using TMD-based sensors, it is imperative to functionalize TMDs with catalysts or selectors. The use of catalysts or selectors promotes reactions with specific target gases, causing the electrons transfer between gas molecules and sensing layers.^375^ In particular, the decoration of TMDs with noble metal NPs has been considered as a viable solution to improve the selectivity of TMD-based chemiresistive sensors. A variety of composite materials consisting of noble metal NPs and TMDs, including Pd-MoS_2_,^376^ Pt-MoS_2_,^377^ Au-MoS_2_,^378^ and Ag-WS_2_,^370^ have been explored for the detection of H_2_ and acetone. For instance, Kuru et al.^376^ reported Pd-functionalized MoS_2_ nanosheets for H_2_ sensors. A simple drop casting of MoS_2_–PdCl_2_ solution and subsequent annealing produced MoS_2_ nanosheets functionalized with Pd NPs (MoS_2_–Pd) (Figure 23a). Interestingly, MoS_2_–Pd exhibited resistance changes (*R*_a_/*R*_g_ = 4.5) upon exposure to 5% H_2_ at room temperature, whereas pristine MoS_2_ nanosheets did not show any response (Figure 23b). The MoS_2_–Pd nanosheets detected 500 ppm of H_2_ with a noticeable response (Figure 23c). The sensing mechanism of MoS_2_–Pd is described by the phase transition of Pd NPs. The phase transition from Pd to PdH_*x*_ caused a change in the electronic band structure of MoS_2_–Pd (Figure 23d),^379^ inducing selective H_2_ sensing properties. In detail, the work function difference between MoS_2_ and Pd causes electron transfer from MoS_2_ to Pd resulting in the formation of a hole accumulation layer in MoS_2_ surface. When exposed to H_2_ gas, PdH_*x*_ is formed which leads to lowering the work function, and electron transfer in the opposite direction, from PdH_*x*_ to MoS_2_. In addition, Cho et al.^378^ reported Au NPs-loaded MoS_2_ nanosheets for selective sensing of VOCs. Due to the difference in the reduction potentials between Au precursors (AuCl_4_^–^, 1.002 V vs SHE) and MoS_2_ (5.2–5.4 eV),^380^ Au precursors could be spontaneously reduced on MoS_2_ flakes. The Au-decorated MoS_2_ nanosheets exhibited distinct sensing behavior toward oxygen-containing VOCs such as ethanol and acetone as compared to pristine MoS_2_ based on effective modulation of electronic structures of MoS_2_ by Au NPs. Likewise, the decoration of metal oxide NPs on TMDs can induce the reactions of adsorbed oxygen species (O_2_^–^ and O^–^) on metal oxides with H_2_O and ethanol, thereby realizing the selective detection of H_2_O^381^ and ethanol.^382^

**Figure 24:**
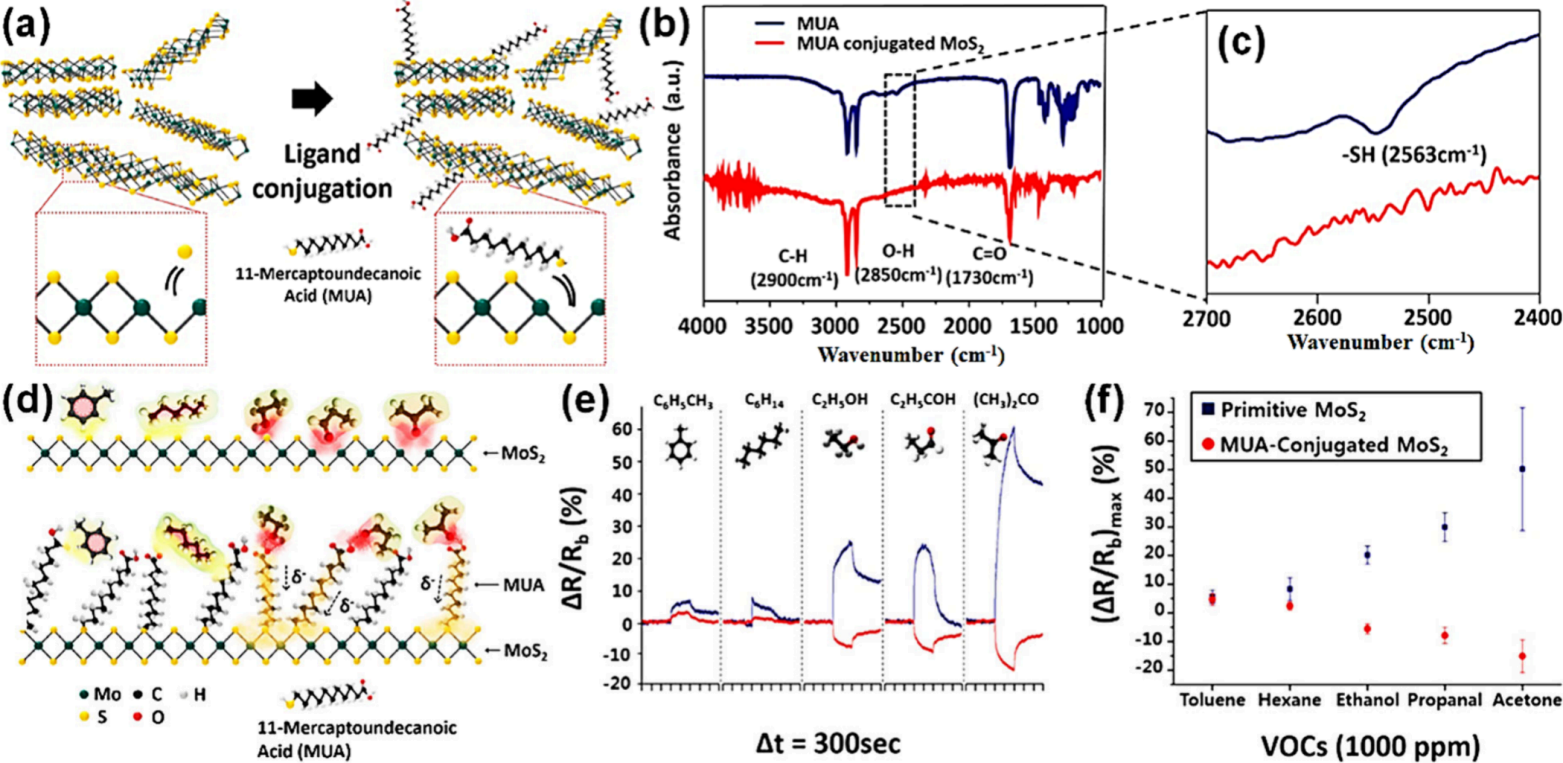
(a) Schematic illustration of the preparation of MUA-conjugated MoS_2_. (b) FT-IR spectra of the samples and (c) enlarged FT-IR spectra in the wavenumber range of 2400–2700 cm^–1^. (d) Schematic description of sensing mechanism of MUA-conjugated MoS_2_. (e) Response traces of pristine MoS_2_ (blue line) and MUA-conjugated MoS_2_ (red line) to various analytes (1000 ppm), (f) and their corresponding responses to various analytes. Reproduced with permission from ref (383). Copyright 2014 American Chemical Society.

In addition, the functionalization of TMDs with organic ligands as selectors can effectively tune the selectivity of TMD-based sensors. Kim et al.^383^ demonstrated that thiolated ligand-conjugated MoS_2_ nanosheets can exhibit selective gas sensing properties to various VOCs. In particular, mercaptoundecanoic acid (MUA) was conjugated with exfoliated MoS_2_ nanosheets via a simple solution mixing process (Figure 24a). FT-IR spectra of MUA and MUA-conjugated MoS_2_ clearly indicated that the thiol groups in MUA were conjugated with sulfur vacancy sites in exfoliated single-layered MoS_2_ (Figure 24b,c). In general, oxygen-containing VOCs strongly interact with sulfur vacancy sites in MoS_2_. However, in the case of MUA-conjugated MoS_2_, VOCs with oxygen groups were adsorbed on MUA instead of MoS_2_ since the surface sites were conjugated with thiol groups in MUA (Figure 24d). As a result, an opposite trend of resistance change was observed when the sensors were exposed to ethanol, propionaldehyde, and acetone (Figure 24e,f), demonstrating that the selective detection of VOCs can be realized by the functionalization of MoS_2_ with organic ligands.

### 6.2 Graphene and Its Derivatives

Recently, graphene has attracted a great deal of attention for gas sensing applications due to its low toxicity as well as excellent electronic and mechanical properties.^384^ Graphene has unique 2D morphological characteristics arising from its large surface area; most significantly, every constituent atom is exposed on the surface and can fully interact with analyte gases.^33^ The adsorption of analyte gases onto graphene can induce a charge transfer that shifts the Fermi energy level of graphene, leading to a change in the resistance. Because of this unique electronic property, graphene has been extensively explored for detecting various gases including NH_3_ and NO_2_. In addition, patterning or generation of pores in graphene sheets have been attempted to maximize structural advantages.^385−388^ However, graphene-based materials exhibit low response for practical use in chemical sensors due to the chemically inert property of pristine graphene, which has a zero or quasi-zero bandgap. To improve the sensing characteristics of graphene, several strategies such as defect engineering, chemical doping, oxidation of graphene into graphene oxide (GO), or reduction of GO to form reduced GO (rGO), have been proposed, and these strategies directly alter the electrical or chemical properties of graphene.^34,389−391^ In addition, the functionalization of graphene or modified graphene with catalytic metal NPs, as well as the combination of SMOs with graphene or modified graphene, are essential approaches for the development of highly sensitive and selective graphene-based sensors.^392−394^ Herein, we review recently reported studies, particularly highlighting strategies adopted to enhance the response and selectivity of graphene with respect to (1) chemical modification and defect engineering, (2) functionalization of graphene with metal NPs, and (3) the combination of graphene with SMOs.

#### 6.2.1 Chemical Modification and Defect Engineering of Graphene

**Figure 25:**
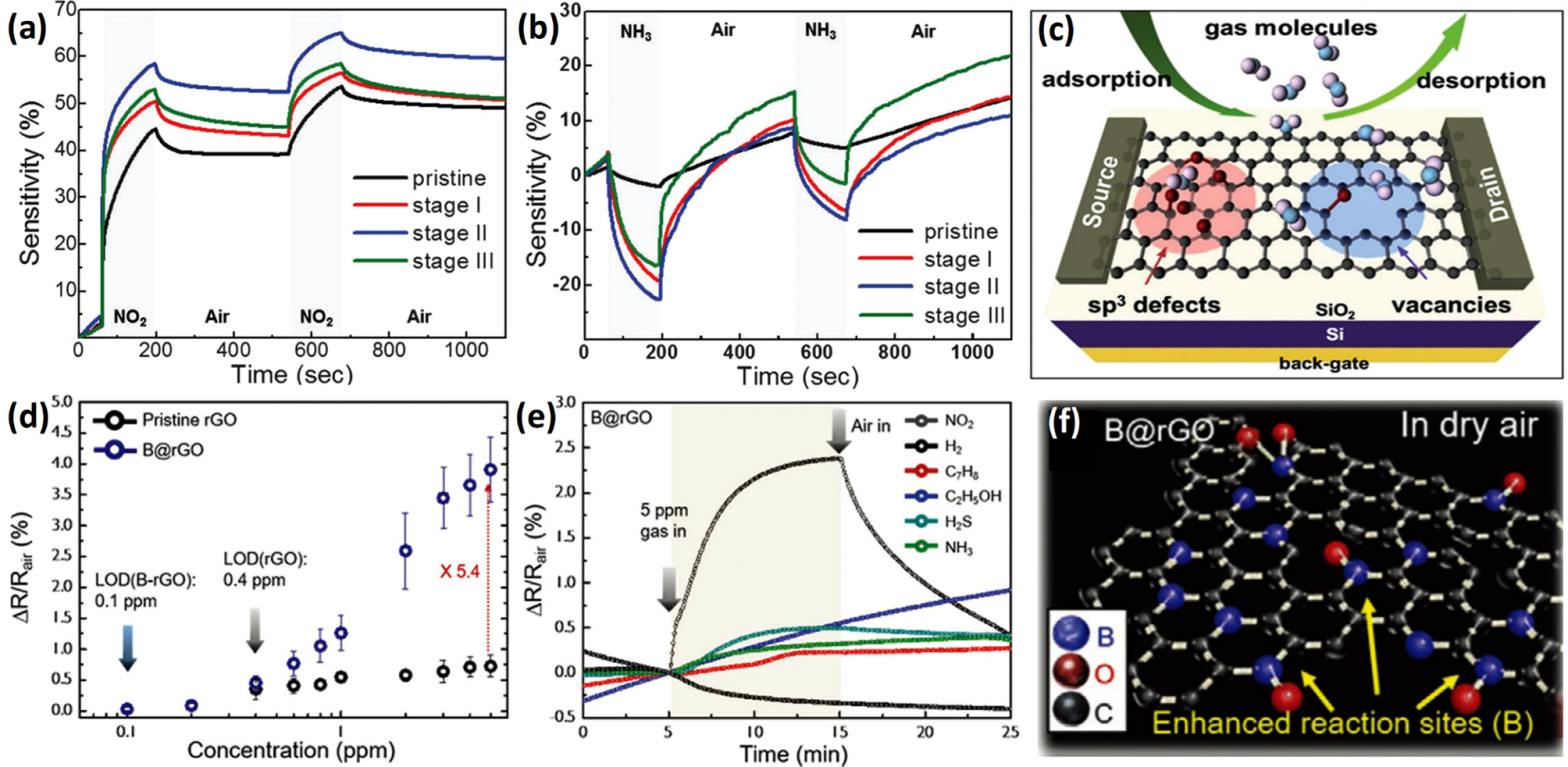
Sensor responses of pristine graphene and defect-engineered graphene sensors for (a) NO_2_ and (b) NH_3_, and (c) schematic of the gas sensing in defect-engineered graphene sensors. Reproduced with permission from ref (399). Copyright 2016 The Royal Society of Chemistry. (d) Response of the pristine rGO and B@rGO to 0.1–5 ppm of NO_2_ at 80% RH. (e) Response of B@rGO to 5 ppm of six different gases, and (f) schematic image of NO_2_ sensing mechanism of B@rGO. Reproduced with permission from ref (401). Copyright 2020 Wiley-VCH.

The unique structural and morphological properties of graphene favor its application in chemical sensors. Although graphene is chemically inert and weakly adsorbs analytes, these issues could be overcome by tailoring its electrical and/or chemical properties by chemical modification and defect engineering. It is well documented that modified graphene displays stronger adsorption ability and higher response.^395−397^ Zhang et al. simulated the interactions of graphene-based sensors (pristine, boron-doped graphene, nitrogen-doped graphene, and defective graphene) with small analyte molecules (CO, NO, NO_2_, and NH_3_) and demonstrated that the response and selectivity of graphene-based sensors can be dramatically improved by introducing dopants and/or defects in graphene.^398^ Lee et al. controlled defects in graphene using reactive ion etching system, and classified the defect states such as sp^3^-type defects (*I*_D_/*I*_G_ < 1 and *I*_2D_/*I*_G_ > 1) and vacancy-type defects (*I*_D_/*I*_G_ > 1 and *I*_2D_/*I*_G_ < 1) according to the ratios *I*_D_/*I*_G_ and *I*_2D_/*I*_G_.^399^ Stage I is defined in sp^3^-type defect, whereas stage II and III are defined by the number of vacancies in vacancy-type defects. The DFT calculations showed that, with an increasing number of defects, the adsorption energy of analyte molecules increased while the hole mobility decreased. As a result, the response for both NO_2_ and NH_3_ was enhanced up to stage II with higher defect density. However, the response decreased at stage III due to the low hole mobility (Figure 25a–c). Especially, it was shown that the response toward NH_3_ would be highly improved compared to that of NO_2_ because of charge transfer between defective graphene and NH_3_ molecules that originally did not exist between defect-free graphene and NH_3_ molecules. In addition to these results, Cui et al. demonstrated the effect of defect engineering in controlling the response and selectivity in graphene-based sensors.^400^

Furthermore, chemical modification by doping is an effective strategy to modify graphene. Lv et al. reported highly improved response toward NO_2_ and NH_3_ of boron-doped graphene, synthesized by chemical vapor deposition (CVD).^402^ Recently, Cha et al.^401^ demonstrated the highly sensitive and selective NO_2_ sensing characteristics of boron-doped rGO (B@rGO), which was prepared by simultaneous reduction of GO while doping with boron via fast Xenon flash irradiation with intense pulsed light. The resultant B@rGO exhibited a superior NO_2_ detection limit as low as 100 ppb, and a 5.4-fold enhanced NO_2_ response compared to that of pristine rGO owing to abundant B active reaction sites in B@rGO, especially under highly humid conditions (80% RH) (Figure 25d). In addition, B@rGO exhibited high selectivity against H_2_, toluene, ethanol, H_2_S, NH_3_, and CO (Figure 25e). These results were attributed to the B-doped active sites, which can provide stronger binding and more abundant sites for reactions with NO_2_ molecules (Figure 25f). In addition, layers of water molecules partially block the direct chemisorption of NO_2_ gas on the B@rGO surface, while electron tunneling can still occur, resulting in resistance modulation during NO_2_ sensing. This inhibition of direct chemisorption facilitates easier desorption during recovery, as supported by enhanced adsorption/desorption kinetics and ex-situ XPS analysis of N 1s peaks for chemisorbed NO_2_^–^ and NO_3_^–^ species. Although chemical modification and defect engineering of graphene is a facile and general strategy for the improvement of the sensing performance of graphene, there is still room for more investigation to further understand the selectivity mechanisms of modified-graphene-based sensors.

#### 6.2.2 Functionalization of Graphene with Metal Catalysts

**Figure 26:**
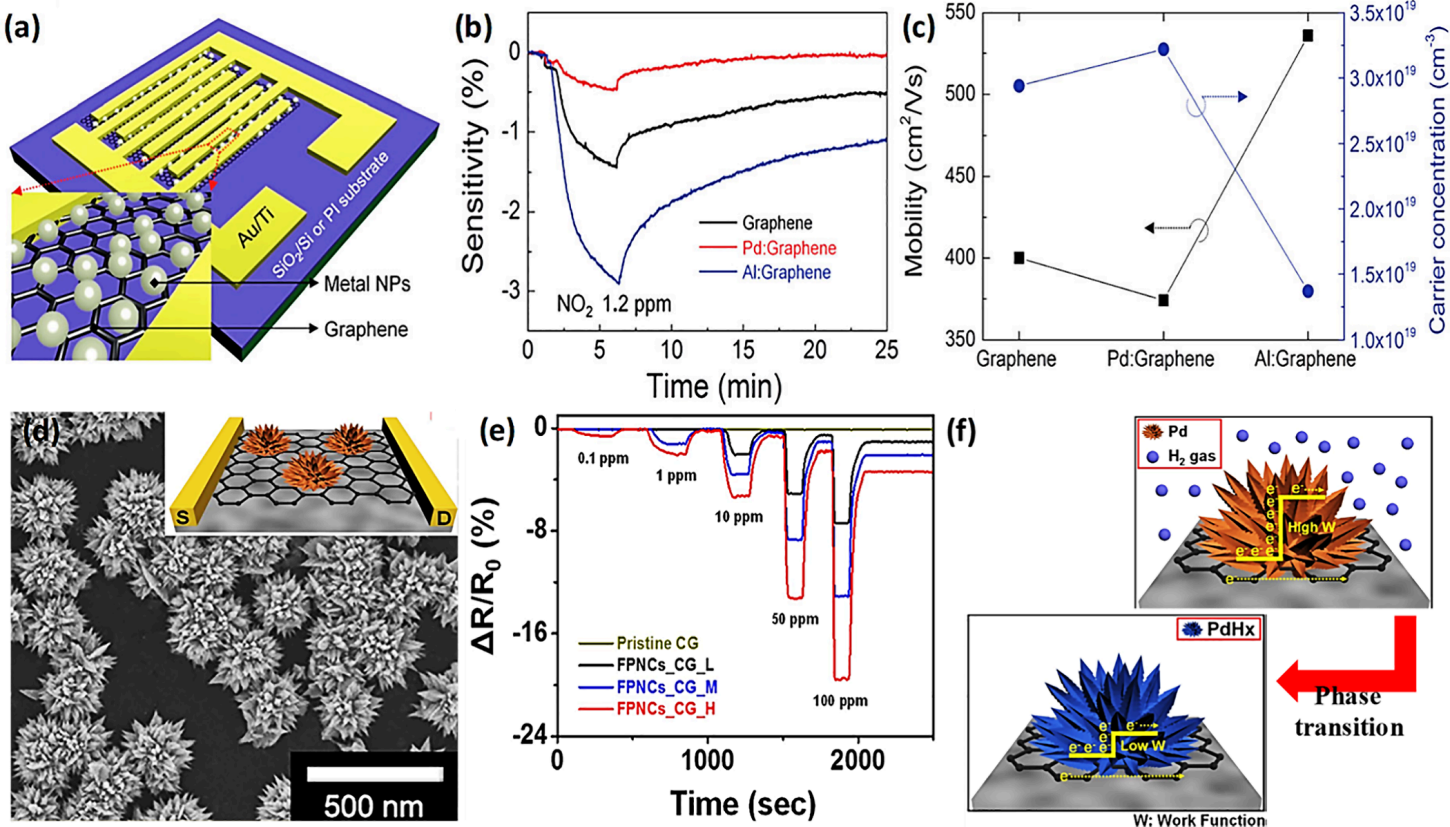
(a) Schematic image of metal decorated graphene. (b) Transient response upon 1.2 ppm of NO_2_ of bare graphene, Pd:graphene, and Al:graphene. (c) mobility and carrier concentration of bare graphene, Pd:graphene, and Al:graphene. Reproduced with permission from ref (407). Copyright 2014 The Royal Society of Chemistry. (d) SEM image and schematic image (inset) of flower-like palladium nanocluster (FPNCs) decorated CVD graphene (CG). (e) Normalized resistance changes upon sequential exposure to H_2_ of various concentration (0.1–100 ppm) at room temperature and (f) schematic image of H_2_ sensing mechanism of FPNCs_CG. Reproduced with permission from ref (408). Copyright 2015 Springer Nature Group.

In addition to directly modifying the properties of graphene, several studies involving the functionalization of graphene and modified graphene such as GO and rGO with metal NPs have been conducted to enhance the selectivity and the response of graphene. Attaching metal NPs such as Pt, Pd, or PtPd alloy on graphene layers can effectively tune the Fermi level of the graphene-based material owing to differences in their work functions, modulating the overall electrical properties.^403−406^ Cho et al. decorated Pd and aluminum (Al) NPs onto graphene using a thermal evaporator, and investigated the sensing performance of doped graphene toward analyte gases (Figure 26a).^407^ The graphene decorated with Al NPs (Al:graphene) exhibited a higher response (Δ*R*/*R*_a_ = 2.89) toward NO_2_ compared to the bare graphene (Δ*R*/*R*_a_ = 1.44), indicating an enhancement of 200% in response. On the other hand, Pd NPs decorated graphene (Pd:Graphene) showed a much lower response (Δ*R*/*R*_a_ = 0.46) toward NO_2_ (Figure 26b). To investigate the effect of metal NPs on graphene, hole mobility and carrier concentration of Al:graphene and Pd:graphene were determined through Hall measurements (Figure 26c). In the case of Al:graphene, the Hall mobility increased, whereas the carrier concentration decreased. This was attributed to the formation of a hole-depleted region at the interfaces of Al NPs and graphene. For Pd:graphene, the Hall mobility decreased, whereas the carrier concentration increased, demonstrating the formation of an ohmic contact between Pd NPs and graphene. Accordingly, the hole-depleted region at the interfaces of Al NPs and graphene facilitated electronic charge transfer from Al:graphene to NO_2_ molecules, resulting in higher response. Ohmic contact in Pd:graphene limited the electron transfer from Pd:graphene to NO_2_ molecules, lowering the response toward NO_2_. In a similar approach, Shin et al. reported flower-like palladium nanoclusters (FPNCs) decorated onto graphene for application as an H_2_ gas sensor (Figure 26d).^408^ The surface of the chemical-vapor-deposited graphene (CG) was treated with 1,5-diaminonaphthalene (DAN) to introduce amino groups, followed by the formation of FPNCs on the surface. The response toward H_2_ increased with the amount of FPNCs, attaining a very low LOD of 0.1 ppm (Figure 26e). The sensing mechanism of FPNCs-decorated CG was attributed to the phase transition of Pd upon exposure to H_2_. As introduced earlier, Pd transforms to PdH_*x*_ upon interaction with H_2_. In this case, the difference in the work functions is modulated to decrease the resistance of FPNCs-decorated CG (Figure 26f), thus achieving selective detection for H_2_. It is envisioned that unprecedented selectivity can be achieved by tuning the catalyst species and controlling the morphology of graphene/catalysts.

#### 6.2.3 Graphene Composites Involving Semiconducting Metal Oxides

**Figure 27:**
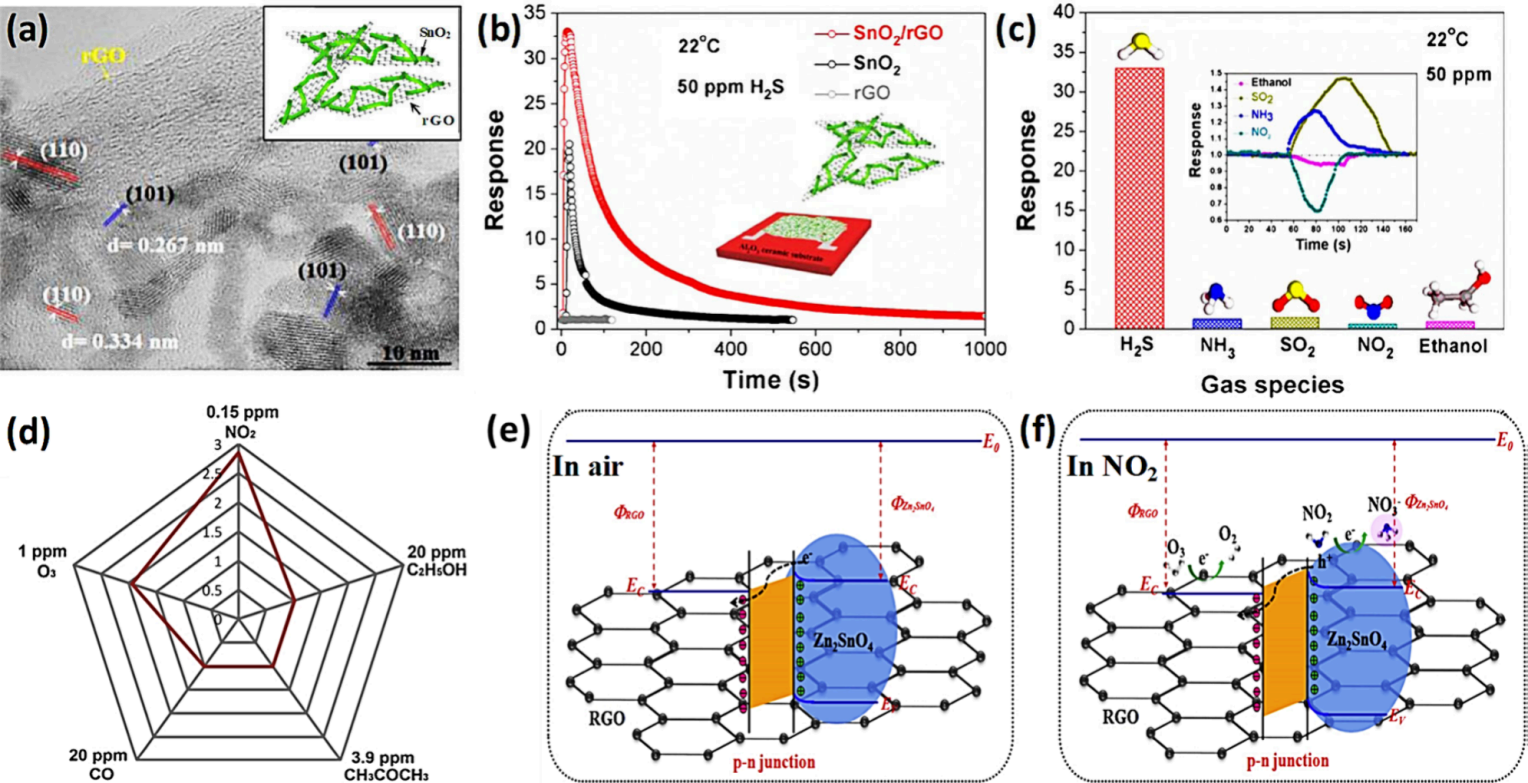
(a) High resolution TEM (HRTEM) image and schematic image (inset) of SnO_2_/rGO nanocomposite. (b) Response curves of gas sensors based on pristine rGO, SnO_2_ quantum wires, and SnO_2_–rGO nanocomposites. (c) Selectivity of SnO_2_–rGO nanocomposite to other interfering gases. Reproduced with permission from ref (413). Copyright 2016 The Royal Society of Chemistry. (d) Selectivity of Zn_2_SnO_4_–rGO hybrid sensor toward different kinds of gases, including 0.15 ppm of NO_2_, 1 ppm of O_3_, 20 ppm of C_2_H_5_OH, 3.9 ppm of CH_3_COCH_3_, and 20 ppm of CO at 30 °C under 50% RH. Schematic image of the energy band structures and the form of p–n heterojunction (e) in air, and (f) in NO_2_. Reproduced with permission from ref (414). Copyright 2019 Elsevier.

SMO-decorated graphene composites have also been widely investigated for gas sensing applications. They exhibit inherent advantages such as high sensitivity, fast response/recovery properties, and good stability. Graphene and its derivatives have low operation temperatures since high operation temperatures can create an oxidation problem, thus a combination of SMOs and graphene can improve the sensing characteristics, surpassing that of the individual components. In addition, Schottky junctions are formed between SMOs and graphene, resulting in a larger variation in resistance and enhanced sensing performance.^409−412^ For instance, Song et al. synthesized well-dispersed crystalline SnO_2_ quantum nanowires anchored onto rGO nanosheets using a one-step colloidal synthesis protocol (Figure 27a, the inset is a schematic of SnO_2_ quantum wire/rGO nanocomposite).^413^ The SnO_2_ quantum wire/rGO nanocomposite exhibited high response (*R*_a_/*R*_g_ = 33) toward H_2_S compared to that observed for pristine rGO or SnO_2_ quantum wires at room temperature (22 °C) (Figure 27b). The improved gas sensing performance was attributed to enhanced electron transport that resulted from charge transfer at SnO_2_/rGO interfaces, and the remarkable conductivity of rGO. In addition, SnO_2_/rGO sensors exhibited highly selective H_2_S sensing against 50 ppm of interfering gases such as NH_3_, SO_2_, NO_2_, and ethanol (all indicating *R*_a_/*R*_g_ < 1.5) at room temperature (Figure 27c). In another report, Wang et al. anchored Zn_2_SnO_4_ NPs on rGO (Zn_2_SnO_4_–rGO) using a one-step hydrothermal synthesis method.^414^ The anchored Zn_2_SnO_4_ NPs acted as a spacer to prevent the agglomeration of rGO sheets, resulting in larger surface area and high porosity necessary for good gas permeability and diffusivity. Furthermore, high carrier mobility of rGO reduced the resistivity of Zn_2_SnO_4_–rGO hybrid, enabling sensor operation at room temperature. As shown in Figure 27d, the Zn_2_SnO_4_–rGO hybrid exhibited excellent NO_2_ selectivity compared to the interfering gases including ethanol, acetone, carbon monoxide, and ozone analytes. Graphene-based materials typically have good selectivity toward oxidizing gases such as NO_2_ at room temperature due to the higher energy for the adsorption of oxidizing gases onto graphene compared to that of reducing gases. In addition, the highly electrophilic NO_2_ could directly capture free electrons from the Zn_2_SnO_4_–rGO hybrid, inducing no changes in the bands of pristine Zn_2_SnO_4_. Most importantly, the p-n heterojunction at the Zn_2_SnO_4_–rGO interface was a vital factor in enhancing the sensing properties (Figure 27e,f). Because of the differences in work function, the electron transfer from Zn_2_SnO_4_ to rGO results in higher resistance of Zn_2_SnO_4_–rGO and improved reactivity with NO_2_. Similar approaches involving various composites of metal oxide and graphene-based forms including In_2_O_3_–rGO nanocomposites,^415^ ZnFe_2_O_4_-graphene quantum dots,^416^ and TiO_2_-3D graphene–carbon nanotubes^417^ have also been reported.

### 6.3 Emerging Two-Dimensional Materials for Chemiresistors

#### 6.3.1 Black Phosphorus

Black phosphorus (BP) shares a layered structure with graphene but distinguishes itself through its orthorhombic puckered configuration. This unique structural attribute, combined with remarkable electronic properties of BP, enhances its chemical adsorption capabilities. The high adsorption energies and plentiful adsorption sites provided by its puckered surface make BP an exceptionally promising material for chemical gas sensors.

**Figure 28:**
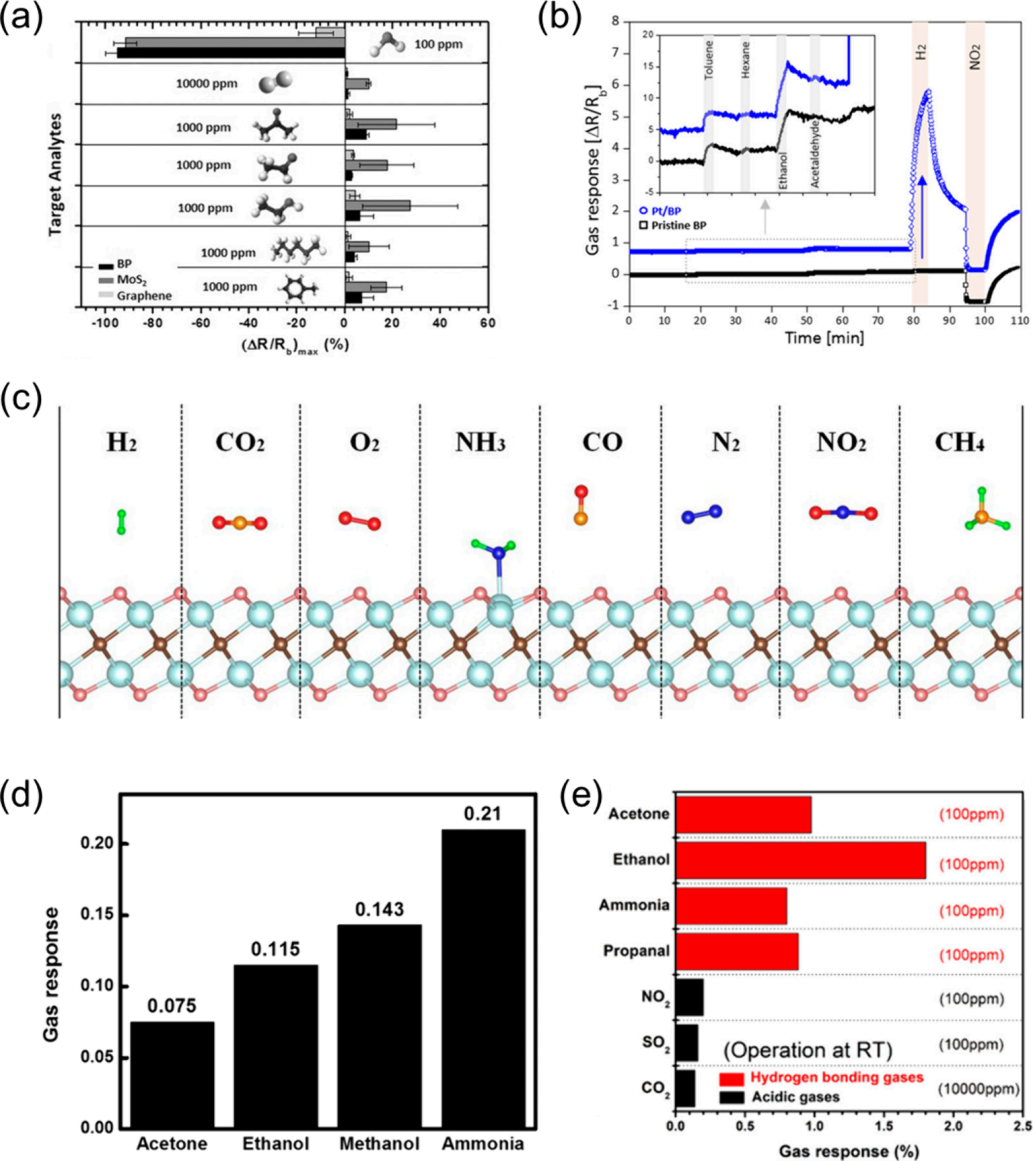
(a) Response values of BP toward various analytes in comparison to MoS_2_ and graphene. Reproduced with permission from ref (418). Copyright 2016 Wiley-VCH. (b) Selectivity tuning of Pt-decorated BP toward H_2_. Reproduced with permission from ref (419). Copyright 2017 American Chemical Society. (c) Schematic illustration of side view of the adsorption of various analyte molecules on monolayer Ti_2_CO_2_. Reproduced with permission from ref (420). Copyright 2015 American Chemical Society. (d) Average response of Ti_3_C_2_T_*x*_ MXene toward four analytes. Reproduced with permission from ref (421). Copyright 2017 American Chemical Society. (e) Response values of Ti_3_C_2_T_*x*_ MXene toward various analytes. Reproduced with permission from ref (422). Copyright 2018 American Chemical Society.

Kou et al. pioneered the computational exploration of various gas species’ adsorption on the surface of BP using first-principles calculations.^423^ They focused on the adsorption interactions of five gas molecules, CO, CO_2_, NH_3_, NO, and NO_2_, with monolayer BP, particularly examining the changes in its electronic properties upon interaction. Their findings indicated that nitrogen-based gas molecules exhibited the strongest binding potential with BP, attributed to significant charge transfers induced by N-containing gases. These calculations suggested exceptional selectivity of BP toward NO_2_, a finding corroborated by subsequent studies. One noteworthy study by Cho et al. highlighted the gas sensing capabilities of BP, where it showed over 90% response to 100 ppm of NO_2_, while showing significantly lower (less than 10%) and reversed responses to much higher concentrations (1000 and 10,000 ppm) of interferents such as hydrogen, acetone, and ethanol.^418^ This study underscored the superior performance of BP compared to other prominent 2D materials like graphene and MoS_2_, demonstrating a 20-fold higher response and 40-fold faster response time, along with ppb-level detection capabilities and superior selectivity toward NO_2_ (Figure 28a). Further DFT calculations supported the experimental data, showing that NO_2_ molecules bind much more strongly to BP than to graphene or MoS_2_, explaining BP’s outstanding selectivity and response characteristics.

Building on their earlier findings, Cho et al. further explored the impact of decorating BP with platinum (Pt) and gold (Au) nanoparticles on its gas sensing properties.^419^ Particularly notable were the results with Pt, where Pt-decorated BP exhibited a remarkable 500% response to 1% H_2_ gas concentration, while maintaining a similar response to NO_2_ as pristine BP (approximately 100% to 100 ppm) (Figure 28b). The enhanced detection performance for H_2_ gas is hypothesized to result from the dissociation of H_2_ molecules on Pt nanoparticles, which reduces the work function of Pt and prompts charge transfer to BP, thereby decreasing hole concentration of BP. Supporting these observations, a DFT study by Ghambarian et al. on Ni, Pd, and Pt-doped BP confirmed that doping BP with Group 10 metals significantly reduces its work function, which is further decreased upon hydrogen adsorption compared to pristine BP.^424^ These findings suggest that the selectivity of BP can be effectively tuned by forming heterostructures with metal elements. While subsequent studies have confirmed the enhanced hydrogen sensing capabilities of metal-decorated BP,^425^ other research indicates improved response and selectivity toward nitrogen oxides rather than hydrogen. Thus, a clearer overall understanding of the effects of metal nanoparticle decoration on selectivity of BP is necessary, highlighting the varying outcomes based on the type of metal used and the targeted gas molecule.

The integration of heterostructures combining 2D materials with metal oxides presents another robust strategy for tuning gas sensing properties. A study by Wang et al. demonstrated this by decorating BP with TiO_2_ nanoparticles using a hydrothermal method.^426^ The TiO_2_-decorated BP exhibited a surprising increase in response, showing a 20% response toward 5 ppm of NH_3_ with a selectivity factor greater than 3 over interferents. However, this study did not explore responses toward nitrogen oxides. Similarly, Ren et al. reported excellent NH_3_ selectivity for BP decorated with SnO quantum dots.^427^ In their research, they did investigate nitrogen oxides, finding that the SnO-modified BP significantly favored NH_3_. Despite these promising results, the precise mechanisms driving these enhancements remain unclear. Additionally, the response characteristics vary with different metal oxide functionalizations: BP/SnO_2_ quantum dot heterostructures were highly selective toward H_2_S,^428^ while ZnO nanowires incorporated into BP improved responses and selectivity primarily toward NO_2_.^429^ These studies suggest that the selectivity of BP-based gas sensors can potentially be fine-tuned through the choice of metal oxide and its form—quantum dots, nanowires, or nanosheets. However, a comprehensive understanding of how these modifications affect selectivity will require further experimental and computational research, examining a broader range of metal oxides and their morphological impacts. This continued investigation is essential to establish a reliable descriptor for the influence of metal oxide functionalizations on the selectivity of BP-based sensors.

#### 6.3.2 MXene

The critical limitations of previously discussed 2D materials involve a notable trade-off: achieving a high signal-to-noise ratio through highly conductive channels typically compromises the response values due to limited surface functionalities. In contrast, MXene presents a unique and compelling alternative among emerging 2D materials. It successfully combines metallic conductivity^430^ with a rich array of surface functionalities, exhibiting both properties without compromising one for the other.^431^ MXene boasts an impressive charge carrier mobility up to 20,000 S cm^–1^,^432^ and its surface is densely populated with functional groups such as −OH, −O, and −F, enhancing its interactions with analyte gases.^421^ This dual capability positions MXene as an exceptional material in the realm of gas sensing technologies.

In computational studies, MXene demonstrated potential detection capabilities for various gases, including NH_3_, H_2_, CH_4_, and CO. Notably, Yu et al. proposed that Ti_2_CO_2_ MXene shows strongest response to NH_3_ due to its ability for chemisorption onto the material surface (Figure 28c).^420^ Experimentally, the first type of MXene evaluated for gas sensing was Ti_3_C_2_T_*x*_ (where T represents surface terminating groups).^421^ This sensor indeed displayed the highest response to NH_3_, with a response value of 0.21, compared to lower responses to interfering VOCs like acetone, which had a response of 0.075 (Figure 28d). However, a subsequent study by Kim et al. using the same Ti_3_C_2_T_*x*_ MXene found a higher response to VOCs, particularly ethanol (response = 1.8%), rather than ammonia (response = 0.8%) (Figure 28e).^422^

This variation could stem from differences in the preparation of the MXene nanosheets. Although both studies used the same documented method for preparation of MXene nanosheets by exfoliation,^433^ Yu et al. created their gas sensing devices via a drop-coating method using a water-based solution on a polyimide substrate with interdigitated electrodes. In contrast, Kim et al. formed their sensors by vacuum filtering the films and then transferring them using an NaOH solution to dissolve alumina membranes. Given the high response of MXene to environmental conditions,^434^ these differing preparation techniques likely altered the proportion of surface functional groups, leading to the observed discrepancies in sensor responses. Further research is needed to explore how the dominant surface functional groups on MXene affect its selectivity toward different gases.

Hybridization with other materials, particularly other 2D materials such as TMDs or graphene derivatives, has proven to significantly enhance the gas sensing performance of MXene.^435−437^ A notable instance is when MXene was hybridized with reduced graphene oxide (rGO) fiber, which markedly increased its response to ammonia—achieving improvements of 7.9 and 4.7 times compared to single-component MXene film and rGO, respectively.^436^ This notable increase in selectivity is believed to be due to the augmentation of active Ti–O sites in MXene upon hybridization. Further evidence of this improvement is seen in a study by Kim et al., where a three-dimensional MoS_2_/MXene heterostructure aerogel demonstrated a more than 2-fold greater response to NO_2_ compared to pristine MXene aerogel, without affecting the response levels to other interfering substances.^435^ However, hybridization does not always lead to enhanced selectivity. For example, Chen et al. reported that while a Ti_3_C_2_T_*x*_/WSe_2_ heterostructure showed a significantly improved general response—12 times greater for ethanol detection compared to pure MXene—there was no discernible enhancement in selectivity among different analytes.^437^ This illustrates that while hybridization can universally enhance sensor response, achieving improved selectivity may depend on the specific materials and methods used in creating the hybrid structures.

MXene, as a relatively newly discovered class of 2D materials, has its gas sensing capabilities, and indeed, its overall physicochemical properties, largely unexplored. This nascent stage of research may contribute to the discrepancies in reported selectivity findings among different studies, even when the same chemical formulation of MXene is used. To establish a more consistent understanding and to define reliable descriptors for selectivity in MXene-based gas sensors, a comprehensive approach involving both experimental and theoretical studies is crucial. Further in-depth research is necessary to resolve these variances and to develop a consensus on the mechanisms that govern the selectivity of MXene in various sensing applications.

### 6.4 Selectivity in Electrically Conductive Metal–Organic Frameworks

Metal–organic frameworks (MOFs) are very advantageous in gas sensing application as they have ultrahigh specific surface area and porosity to facilitate diffusion and adsorption of gas molecules. In addition, their high tunability based on combinations of nodes and linkers has a great potential to target a wide range of analytes.^438,439^ However, the low electrical conductivities of typical MOFs have limited their application in chemiresistive sensors. Recently, electrically conductive MOFs (cMOFs), typically composed of layered 2D structures with π-conjugation, have been developed.^440^ As their high electrical conductivities enable room-temperature operation with the above merits, they have emerged as promising chemiresistive materials. Accordingly, cMOFs-based chemiresistors have been demonstrated for sensing of various gases, including NH_3_, H_2_S, NOx, and VOCs.^441−444^ Although current cMOF-based sensors have not reached sufficient levels of response and selectivity, their versatile structures offer a bright prospect for designing highly selective sensing materials. In this section, we highlight two approaches, node/linker control and integration of foreign materials, for modulating selectivity in cMOF-based sensors.

#### 6.4.1 Control of Nodes and Linkers in cMOFs

**Figure 29:**
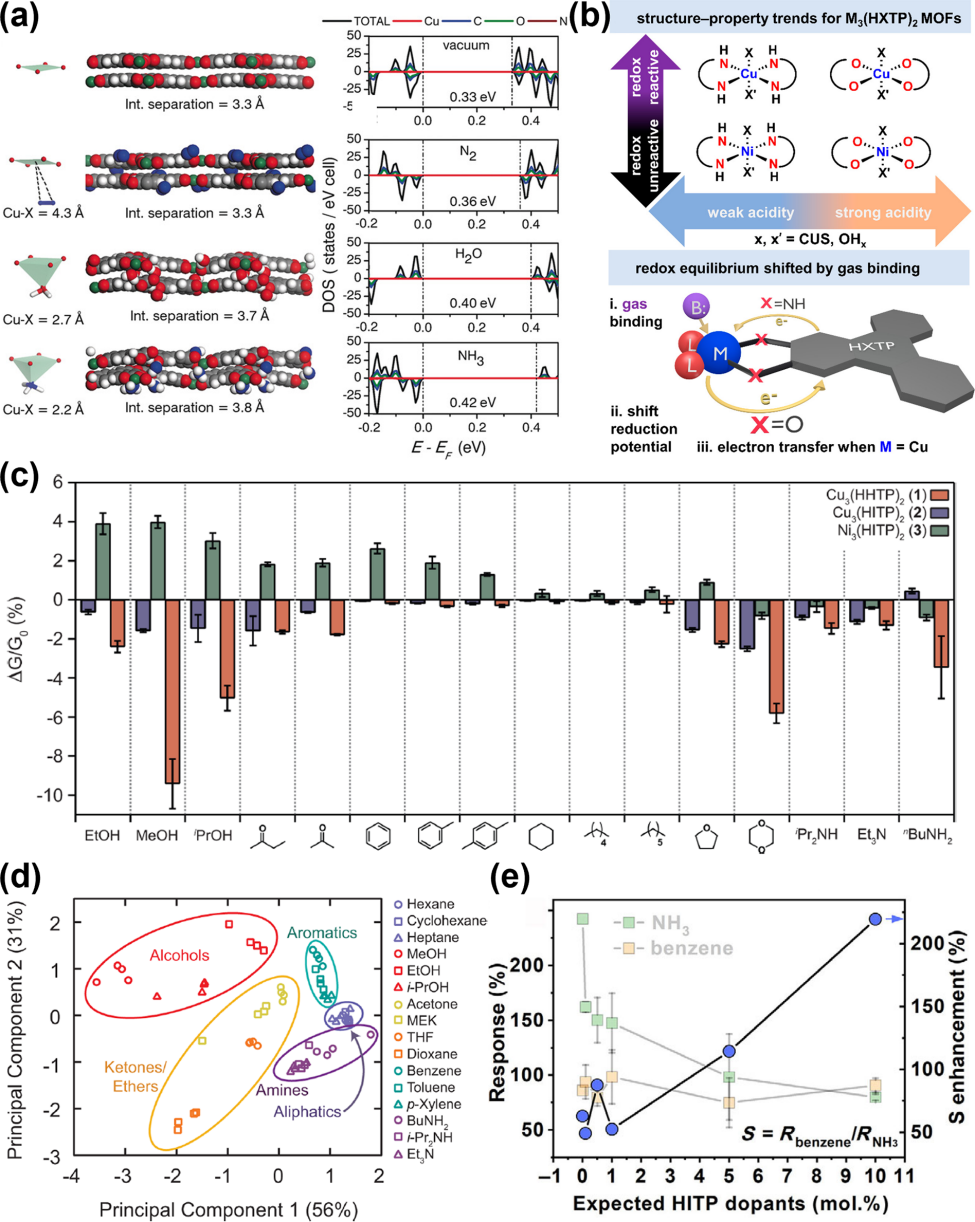
(a) DFT calculated structure and electronic density of states of Cu_3_(HHTP)_2_ in N_2_, H_2_O, and NH_3_.^445^ Reproduced with permission from ref (445). Copyright 2017 Wiley-VCH. (b) Trends in structure and property of cMOFs and mechanism for redox equilibrium upon gas binding.^438^ Reproduced with permission from ref (438). Copyright 2020 American Chemical Society. (c) Sensing response of cMOFs array toward various VOCs.^442^ (d) PCA anaylsis of array for VOCs.^442^ Reproduced with permission from ref (442). Copyright 2015 American Chemical Society. (e) Selectivity change in Cu_3_(HHTP)_2_ upon varying doping concentration of HITP.^446^ Reproduced with permission from ref (446). Copyright 2021 Springer Nature.

It is well-known that chemical properties of MOFs and their interactions with guest molecules can be designed by the selection of nodes and linkers in MOFs.^447−449^ Therefore, the selection of nodes and linkers is a primary factor for determining the selectivity of cMOF-based chemiresistors. In the first study on cMOF-based chemiresistors, Cu_3_(2,3,6,7,10,11-hexaiminotriphenylene), Cu_3_(HITP)_2_, exhibited a noticeable response toward <0.5 ppm of NH_3_.^441^ On the other hand, analogous Ni_3_(HITP)_2_ showed no response, which could be originated from different d electron count of metal node (d^8^ in Ni^II^ vs d^9^ in Cu^II^) and the corresponding change in Fermi level. Rubio-Giménez et al. observed the larger change in the electronic structure of cMOFs upon stronger coordination of analytes on the metal center, suggesting that the coordination affinity of analytes on the metal node is related to the sensor response (Figure 29a).^445^ These results reveal the importance of the metal node in the selectivity of cMOF-based chemiresistors. On the other hand, linkers also have a great impact on the interaction of gases. The interaction between the basic molecules and cMOFs showed that the linkers influence the surface acidity, thereby affecting analyte binding. Hexahydroxytriphenylene (HHTP) based cMOFs provide stronger Lewis or Brønsted acid sites than HITP, promoting stronger binding of basic NH_3_ (Figure 29b).^438^ In addition, as noninnocent ligands, these triphenylene linkers participate in the redox reaction to counterbalance the oxidation or reduction of metal node upon coordination of analytes. The redox balance may affect the electronic structure and response of cMOF-based sensors. In addition, heteroatoms of linkers, such as O or NH, may directly provide binding sites for analytes, leading to a linker-dependent response.^442^ For example, the conductivity changes are opposite in Ni_3_(HHTP)_2_ and Ni_3_(HITP)_2_ upon exposure of NO.^443^

Designing cMOFs for target analytes is challenging at this stage as sensing mechanism has not been clearly elucidated. However, switching the node and linker of cMOF still provides a facile way to impart different selectivity to sensors for targeting a wide range of gases. Utilizing the high tunability of cMOFs, sensor arrays have been constructed from isostructural cMOFs to differentiate various gases through principle component analysis (PCA). Campbell et al. prepared sensor array from three analogous cMOFs (Cu_3_(HHTP)_2_, Cu_3_(HITP)_2_, and Ni_3_(HITP)_2_), which exhibit dissimilar responses toward various analytes (alcohols, ethers, ketones, amines, and aromatic/aliphatic compounds) (Figure 29c,d).^442^ With PCA and linear discriminant analysis, it is demonstrated that analytes could be discriminated with 92% accuracy. Similarly, Smith et al. constructed sensor arrays by replacing nodes or linkers in triphenylene-based cMOFs to distinguish NH_3_, NO, H_2_S, and H_2_O.^443,450^ This approach is not only applicable to triphenylene-based cMOF. Nickel phthalocyanines were employed as linkers to synthesize four analogous bimetallic cMOFs with different sensing behaviors, thereby enabling differentiation of NO, NH_3_, and H_2_S with PCA.^451^ The advantage of phthalocyanine-based cMOFs is that linkers themselves can incorporate different metal centers, adding another variable for tuning selectivity. Cobalt-phthalocyanine-based cMOFs (CoPc-Cu) showed a higher response toward CO than NiPc-Cu, which could be explained by stronger binding and charge transfer of CO on cobalt sites.^452^ The enhanced response toward CO enables the discrimination of NO, NO_2_, and CO with the array of cMOFs. On the other hand, selectivity can be continuously adjusted by partly replacing linker of cMOF with another analogous linker. Upon increasing the doping concentration of HITP in Cu_3_(HHTP)_2_ up to 10%, NH_3_ response gradually decreases while improving response toward benzene.^446^ Accordingly, HITP-doped Cu_3_(HHTP)_2_ can target benzene, which has a lower reactivity and polarity compared to NH_3_, reversing selectivity (Figure 29e).

#### 6.4.2 Integration of Foreign Components into cMOFs

**Figure 30:**
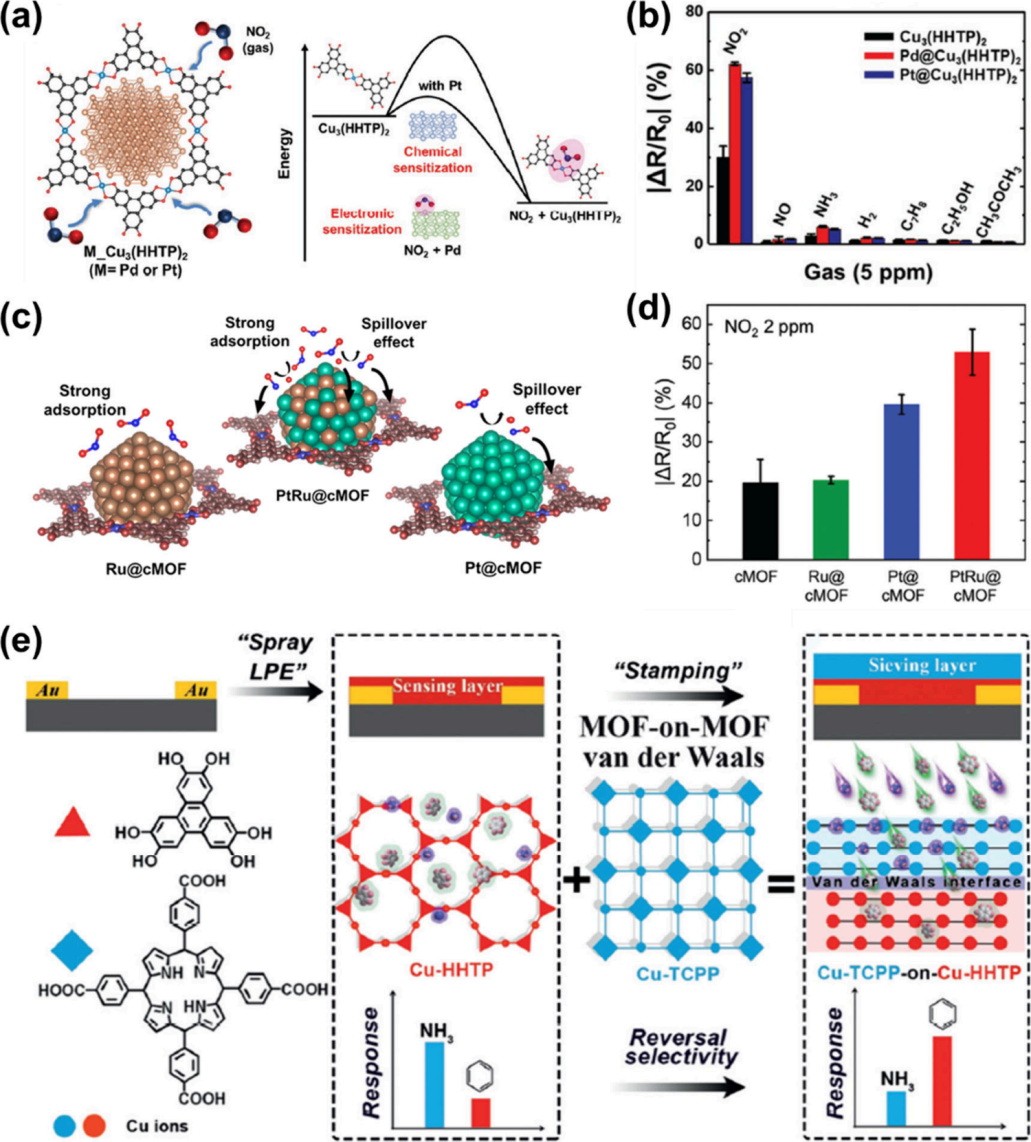
(a) Schematic illustration for sensing mechanism of Pt@Cu_3_(HHTP)_2_ and Pd@Cu_3_(HHTP)_2_. (b) Selectivity of Cu_3_(HHTP)_2_, Pt@Cu_3_(HHTP)_2_, and Pd@Cu_3_(HHTP)_2_.^454^ Reproduced with permission from ref (454). Copyright 2019 Wiley-VCH. (c) Schematic illustration for bifunctional mechanism of bimetallic Pt–Ru NPs on Cu_3_(HHTP)_2_.^444^ (d) Sensing response of Cu_3_(HHTP)_2_, Pt@Cu_3_(HHTP)_2_, Ru@Cu_3_(HHTP)_2_, and PtRu@Cu_3_(HHTP)_2_ toward 2 ppm of NO_2_.^444^ Reproduced with permission from ref (444). Copyright 2021 Wiley-VCH. (e) Schematic illustration for fabrication of MOF-on-MOF heterostructures.^456^ Reproduced with permission from ref (456). Copyright 2020 Wiley-VCH.

As mentioned above, it is difficult to rationally design cMOFs for specific targets because the gas sensing mechanisms of cMOFs are complicated and incompletely understood. One possible alternative is the introduction of foreign components, for which reaction mechanisms with gases are relatively well-understood, to improve the response and selectivity for target analytes. MOFs in particular are renowned for well-defined porous structures, which are very advantageous for stabilizing NPs.^453^ Koo et al. synthesized Pt@Cu_3_(HHTP)_2_ and Pd@Cu_3_(HHTP)_2_ by infiltration of metal ions in pores and subsequent chemical reduction.^454^ Utilizing the catalytic effect of Pt NPs which promotes the reaction of NO_2_ by a spillover effect, Pt@cMOF showed a higher response toward 5 ppm of NO_2_ (Δ*R*/*R*_0_ = −57.38%) compared to cMOFs (Δ*R*/*R*_0_ = −29.95%) and excellent selectivity with a 10-fold higher response for NO_2_ than interfering gases (Figure 30a,b). Similarly, Pd@cMOFs exhibited high NO_2_ response (Δ*R*/*R*_0_ = −62.11%) and selectivity based on the electronic sensitization effect of Pd NPs. Note that these NPs@cMOF structures also can be fabricated as film-type chemiresistors via microfluidic-based solution shearing.^455^ In addition, it is possible to stabilize ultrasmall NPs containing more than one element via site-specific growth in the pores of cMOFs. Encapsulation of bimetallic Pt–Ru NPs in cMOF further improved the NO_2_ sensitivity based on a bifunctional mechanism combining the strong adsorption of NO_2_ on Ru and a spillover effect by Pt (Figure 30c,d). It might be possible to modulate the response and selectivity for a wider variety of gases by exploring different combinations of metal elements in encapsulated multimetallic NPs in cMOFs.

Considering the various interactions of MOFs with gases, other MOFs can be attractive candidates as foreign components to control the selectivity of cMOFs. Cu_3_(HHTP)_2_ exhibits considerable response toward benzene, but its stronger response toward NH_3_ has hindered detection of benzene. Yao et al. reversed the selectivity by using another MOF, Cu-TCPP, as a sieving layer, which can limit the penetration of NH_3_ (Figure 30e).^456^ To obtain MOF-on-MOF heterostructure, Cu_3_(HHTP)_2_ film was first fabricated as a sensing layer by liquid phase epitaxial growth. Since Cu-TCPP cannot form direct covalent or coordination bonds with the structurally dissimilar Cu_3_(HHTP)_2_, Cu-TCPP nanosheets were first synthesized and transferred onto Cu_3_(HHTP)_2_ as a separate layer. Cu-TCPP layer selectively lowered the response toward NH_3_, maintaining the response toward benzene. As a result, the selectivity toward benzene (*S* = *R*_benzene_/*R*_NH3_) was increased up to 250%. In addition, the selectivity of heterostructures could be gradually modulated by varying the number of transferred layers (or thickness) of Cu-TCPP. By constructing an array from these heterostructures, common biomarkers (acetone, CO, hexane, benzene, and NH_3_) could be distinguished through PCA.

#### 6.4.3 Future Prospects in cMOF-Based Chemiresistors

The sensing response of cMOF does not simply originate from charge transfer but involves multiple, often unique, sensing mechanisms. While this uncommon behavior makes it difficult to elucidate sensing mechanisms, it helps cMOFs be complementary to conventional sensing materials by imparting unique selectivity to them. For example, Cu_3_(HITP)_2_ shows opposite responses to amines with different substituents, which are considered to be similar reducing gases in a typical sensing mechanism.^442^ In addition, cMOFs have distinct binding sites depending on facets, including interior pores, basal plane, and linker or node at the edge, which can undergo different interactions with gases.^438^ Since their proportion or accessibility can be controlled by orientation, delamination, and growth control,^457−459^ tailoring these binding sites can be another design principle for further tuning their selectivity in addition to the combination of linkers and nodes. However, to take full advantage of the tunability of cMOFs, it is necessary to elucidate sensing mechanisms toward more various gases. Especially, research on cMOF-based chemiresistors has focused on only a few reactive and polar gases such as NO_*x*_, H_2_S, and NH_3_, although high structural/chemical diversity is a key advantage of cMOFs. Since discrimination between relatively less polar VOCs is also highly demanding and challenging issue in gas sensor field, sensing behaviors of cMOFs on these gases need to be further investigated.

Moreover, the integration of cMOFs with composite materials, such as graphene, carbon nanotubes, and metal oxides, has opened new avenues for advancing gas sensor performance.^460,461^ These composites enhance electronic modulation, create efficient charge conduction pathways, and improve selectivity and sensitivity through synergistic interactions between cMOFs and the supporting materials. By leveraging the high structural tunability of cMOFs alongside the functional versatility of composites, new gas sensing technologies can address the challenges of VOC discrimination and beyond, paving the way for next-generation gas sensors with unprecedented performance. Beyond gas sensing, cMOFs show promise in flexible electronics, particularly in electrochemical transistors, where their high porosity and aligned pore structures enable efficient ion diffusion, high volumetric capacitance, and rapid response speeds.^462^ These advancements highlight the potential of cMOFs for multifunctional sensing and flexible device applications.

### 6.5 Challenges in 2D Materials-Based Chemiresistive Sensors

Although atomically thin 2D materials such as TMDs exhibit excellent semiconducting performances and possess fascinating physical and chemical properties that are desirable for gas sensing,^463,464^ intensive exploration is still required to unlock novel sensing properties. Some of the key obstacles to the use of TMDs include the absence of a clear fundamental sensing mechanism for some TMDs, low response compared to SMOs counterparts, sluggish response/recovery kinetics and stability, and difficulty in achieving controllable doping in 2D lattices using traditional synthesis techniques.^55,465−467^ In addition, only a few gases such as NH_3_, NO_2_, and NO, have been investigated with TMDs for selective sensing applications (Table 5). For graphene, despite having large theoretical surface area and excellent carrier mobility, it has a zero band gap, and lacks functional groups for strong adsorption of gas molecules.^468^ As observed, defect engineering, doping, modification into GO and rGO, and surface immobilization of metal or metal oxide NPs on graphene are generally employed to utilize graphene in gas sensing applications. Ideally, substitutional doping introduces defects in graphene, enabling interaction with gas molecules.^398^ However, defects in graphene also act as sources of noise, which can degrade the electrical quality and response and counteract the aforementioned benefits.^469,470^ As shown in Table 6, the response values of recently reported graphene, graphene derivatives as well as metal oxide decorated graphene composites are still lower compared to those of SMOs shown in Table 1 and Table 2. Taken together, there is still much demand for further investigations into 2D materials to unlock their full potential toward gas sensing applications.

**Table 5:** Recently Reported Gas-Selective Sensors Based on Various 2D Transition Metal Dichalcogenides and Their Modified Forms

sensing material	response definition	response/conc (ppm)	target gas	interferents	ambient condition	ref
monolayer MoS_2_	Δ*I*/*I*_0_	300% to 50 ppm	NH_3_	not investigated	air, RT	(362)
single-layer MoS_2_ with green light	Δ*R*/*R*_0_	70% to 200 ppm	NH_3_	not investigated	N_2_, RT	(55)
single-layer MoSe_2_	Δ*R*/*R*_0_	1100% to 500 ppm	NH_3_	not investigated	Ar, RT	(366)
MoTe_2_ with gate voltage	Δ*G*/*G*_0_	30% to 2 ppm	NH_3_	not investigated	N_2_, RT	(471)
SnS_2_ with white light	Δ*I*/*I*_0_	500% to 500 ppm	NH_3_	HCl, H_2_O_2_, dimethyl carbonate, hexane, acetone, H_2_O, CH_3_CN, and DMC	air, RT	(136)
MoS_2_	Δ*I*/*I*_0_	80% to 2 ppm	NO	not investigated	N_2_, RT	(360)
vertically aligned MoS_2_	Δ*R*/*R*_0_	10% to 100 ppm	NO_2_	not investigated	N_2_, RT	(368)
Pt-MoS_2_	Δ*I*/*I*_0_	16% to 1.2 ppm	NO_2_	not investigated	N_2_, RT	(377)
graphene/MoS_2_	Δ*R*/*R*_0_	110% to 1 ppm	NO_2_	NH_3_, H_2_, and H_2_S	N_2_, RT.	(472)
MoS_2_ with UV light	Δ*R*/*R*_0_	35% to 100 ppm	NO_2_	NH_3_, H_2_, H_2_S, CO_2_, and CH_4_	Ar, RT.	(373)
MoS_2_ with led light	Δ*R*/*R*_0_	12% to 200 ppb	NO_2_	not investigated	N_2_, RT	(372)
ultrathin WS_2_	Δ*R*/*R*_0_	36.9% to 1 ppm	NO_2_	not investigated	air, RT	(369)
WS_2_–Ag nanowire	Δ*G*/*G*_0_	650% to 500 ppm	NO_2_	not investigated	air, 100 °C	(370)
WS_2_–carbon NFs	Δ*R*/*R*_g_	15% to 1 ppm	NO_2_	NH_3_, toluene	air, RT	(363)
WS_2_–carbon composites	Δ*R*/*R*_g_	18% to 1 ppm	NO_2_	H_2_S, NH_3_, ethanol, acetone, toluene	air, RT	(364)
MoSe_2_	Δ*I*/*I*_0_	5% to 5 ppm	NO_2_	not investigated	N_2_, RT	(473)
MUA-conjugated MoS_2_	Δ*R*/*R*_0_	7% to 100 ppm	acetone	toluene, hexane, ethanol, propanol	N_2_, RT	(383)
MoS_2_–Au NPs	Δ*R*/*R*_0_	40% to 1000 ppm	acetone	acetaldehyde, ethanol, hexane, toluene	N_2_	(378)
WS_2_–Ag nanowire	Δ*G*/*G*_0_	50% to 10 ppm	acetone	not investigated	air, 100 °C	(370)
MoS_2_ on porous Si	Δ*R*/*R*_0_	5% to 5 ppm	ethanol	H_2_O, benzene, toluene, isopropyl alcohol, acetone, methanol	air, RT	(474)
MoS_2_–Pd NPs	Δ*R*/*R*_g_	4.5 to 50000 ppm	hydrogen	not investigated	N_2_, RT	(376)
MoS_2_–Pd	Δ*R*/*R*_0_	35% to 10000 ppm	hydrogen	not investigated	air, RT	(379)
monolayer MoS_2_	Δ*G*/*G*_0_	3% to 1 ppm	trimethylamine	tetrahydrofuran, methanol, nitrotoluene, 1,5-dichloropentane, DCB, 1,4-dichlorobenzene	N_2_, RT	(475)

**Table 6:** Recently Reported Gas-Selective Sensors Based on Graphene, Graphene Derivatives, and Metal Oxide Modified Graphene Composites

sensing material	response definition	response/conc (ppm)	target gas	interferents	ambient condition	ref
graphene based single yarn	Δ*R*/*R*_a_	12% to 1.25 ppm	NO_2_	ethanol, ethylene, acetone, CO_2_	air, RT	(476)
graphene-PEDOT:PSS	Δ*R*/*R*_a_	1.2% to 5 ppm	NH_3_	diethylamine, acetone, ethanol, methanol, toluene	air, RT	(477)
3D reduced graphene oxide hydrogel (RGOH)	Δ*R*/*R*_a_	3.74% to 10 ppm	NO_2_	NH_3_, CO_2_, acetone, ethanol, methanol, chloroform, DMF	N_2_/air, 22 °C	(478)
rGO–PANI	*R*_g_/*R*_a_	344.2% to100 ppm	NH_3_	C_6_H_5_CH_3_, HCHO, CH_3_OH, CH_3_COCH_3_, C_2_H_5_OH	air, RT	(479)
Zn_2_SnO_4_–rGO	*R*_a_/*R*_g_	5.97/1 ppm	NO_2_	O_3_, C_2_H_5_OH, CO, CH_3_COCH_3_	air, 30 °C	(414)
In_2_O_3_–rGO	*R*_a_/*R*_g_	8.25/30 ppm	NO_2_	C_2_H_5_OH, CH_4_, H_2_, CO, CH_3_OH, C_8_H_10_, C_3_H_3_O	air, RT	(415)
flower-like Pd decorated graphene	Δ*R*/*R*_a_	6%/10 ppm	H_2_	NO_2_, NH_3_	N_2_, RT	(408)
SnO_2_–rGO	*R*_a_/*R*_g_	33/50 ppm	H_2_S	NH_3_, SO_2_, NO_2_, ethanol	air, 22 °C	(413)
rGO–carbon dot	Δ*R*/*R*_a_	120%/25 ppm	NO_2_	chloroform, ethanol, toluene, ammonia, DCM, methanol, DMF, DMMP, hexane, acetone	air, RT	(480)
3D epitaxial graphene nanowalls	Δ*I*/*I*_o_	77%/500 ppm	O_2_	N_2_ and H_2_	RT	(385)

## 7 Future Perspectives on Research and Development

### 7.1 New Synthetic Approaches for Catalyst Decoration

#### 7.1.1 Transient High-Temperature Annealing Approaches

Based on high thermal-budget efficiency, versatility, and tunability, several momentary high-temperature annealing approaches such as microwave shock,^481^ joule-heating-induced thermal shock,^482−484^ and light-induced photothermal shock^485−487^ have been reported as effective annealing methods to functionalize catalytic NPs on carbon- or oxide-based sensing scaffolds. These methods take advantage of millisecond-scale annealing at high-temperature with ultrafast heating and cooling rates (up to 10^5^ K s^–1^), which enables generating a far-from-equilibrium synthesis regime to explore new catalytic materials with unexpected catalytic characteristics.

In the case of microwave-assisted method, the rapid oscillating electric field of microwaves effectively transfers the electromagnetic energy to the target materials to induce rapid heating.^481,488^ Meanwhile, joule-heating method utilizes the heat generated by the resistance of conductive supports by applying current. Hu et al. first reported joule-heating-based momentary high-temperature annealing method (>3000 K within 1 s) using carbon NFs as supporting materials to decorate catalytic NPs with the unary composition to high-entropy alloys and even SACs.^482,483^ In the transient high temperature annealing process, conventionally immiscible elements can be formed into atomically mixed high-entropy alloy NPs without any phase separations. The advantages of the joule-heating approach include that by controlling the power, and the number and duration of pulses applied to the conductive supports, the maximum temperature could be precisely manipulated to engineer the size and distribution of catalytic NPs.^489−491^ Since the joule-heating approach requires high conductivity of the target materials, carbon-based supports including carbon NFs, graphene, and carbonized wood, have been mainly studied.^492^ Meanwhile, by introducing subsequent synthetic procedures, joule-heating-induced catalytic NPs can be successfully transferred to neighboring oxide supports. Chae et al. reported Pt NPs-decorated WO_3_ nanotubes as effective H_2_S gas sensing layers by introducing joule-heating and subsequent sputter-deposition processes.^493^ After decoration of catalytic Pt NPs on carbon NFs using joule-heating, WO_3_ overlayers were sputter-deposited followed by subsequent calcination in air atmosphere to thermally decompose carbon NFs and to transfer Pt NPs on the porous WO_3_ layers. This work successfully demonstrated the feasibility of joule-heating-assisted decoration of catalytic NPs on oxide supports, which can be expanded to various composition of catalytic NPs and oxide supports to bring different gas selectivities.

**Figure 31:**
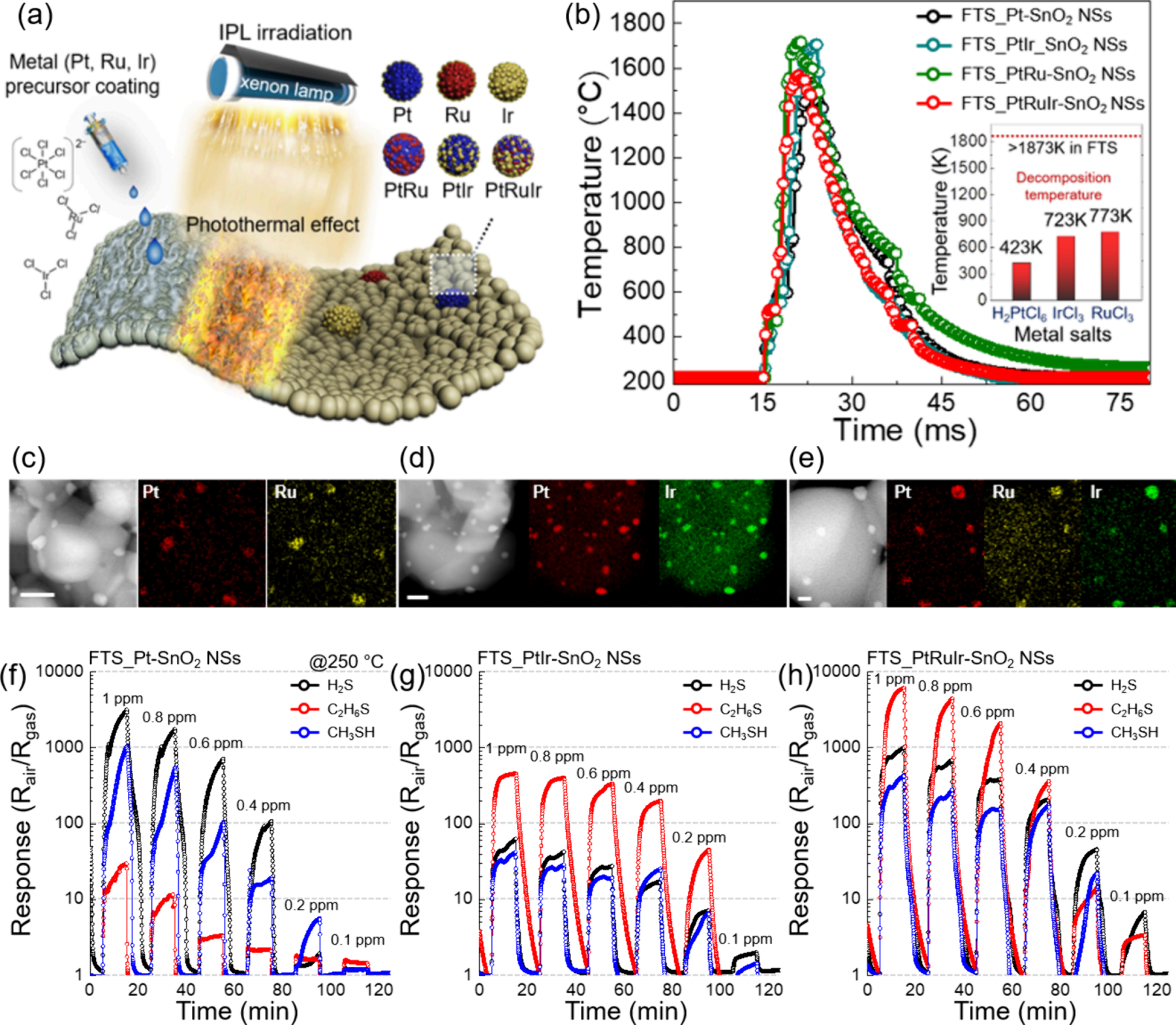
(a) Schematic illustrations of the synthesis of catalysts-stabilized SnO_2_ nanosheets (NSs) via IPL treatment. (b) Temperature–time curves of catalysts-precursors loaded SnO_2_ NSs under IPL treatment. STEM elemental maps of (c) FTS_PtRu-SnO_2_ NSs, (d) FTS_PtIr-SnO_2_ NSs, and (e) FTS_PtRuIr-SnO_2_ NSs. Gas sensing characteristics of (f) FTS_Pt-SnO_2_ NSs, (g) FTS_PtIr-SnO_2_ NSs, and (h) FTS_PtRuIr-SnO_2_ NSs. Reproduced with permission from ref (494). Copyright 2022 Cell Press.

The synthetic methods using high-intensity light utilize either laser with a specific short-wavelength or a lamp with various longer-wavelengths. The basic principle of light-induced synthesis is that when a material with a specific bandgap is irradiated with light having a higher energy than the bandgap, the stimulated electrons relax back to a lower energy state, converting the lost energy into the emission of photons or nonradiative lattice phonons to generate thermal energy upon interaction with defect sites.^495^ Lamps feature a high output efficiency and large beam area (cm^2^ scale) for a large-scale processability. However, lamps with a wavelength range of 300–1000 nm lack energy density to effectively trigger photothermal effects on large bandgap oxides, hampering its wide use. To overcome the issue, Kim et al. suggested that control of the defect density, bandgap type, crystallite size, and porosity of oxide supports could successfully enhance the photothermal efficiency to induce transient high-temperature (>1800 °C with a duration time of 20 ms) (Figure 31a,b).^486^ Precoating of metal ion precursors on oxide supports and subsequent lamping led to the direct formation of multielemental catalytic NPs including PtRu, PtIr, and PtRuIr (Figure 31c–e). As a result, Pt- or PtRuIr-decorated SnO_2_ showed selective chemiresistive responses toward hydrogen sulfide or dimethyl sulfide gases, respectively, with extremely high responses (Figure 31f–h). Interestingly, introducing multiple pulses of flash lamping could downsize the catalytic NPs even into atomically isolated metal species. The flash lamping approach is simple, yet versatile thus decoration of diverse high-entropy alloys and/or single-atom-scale with tunable composition and size could strengthen the strategies to prepare high-performance gas sensing layers with tunable gas selectivity. We anticipate that elaborate integration of several transient high-temperature annealing methods, e.g. joule-heating and flash lamping, would further expand the type of catalysts and support materials.

#### 7.1.2 Ex-Solved Metal Nanoparticle Catalysts

Securing a high surface-to-volume ratio by making the catalytic metal particles smaller is essential for improving catalytic atom efficiency. Other key aspects to consider when designing supported nanocatalytic systems are high dispersion of the catalysts exclusively on the surface, uniform size distribution among the catalyst NPs, as well as the tolerance of the metal NPs toward poisoning, degradation and agglomeration. Conventional approaches for the synthesis of supported nanocatalysts have not only been unsuccessful in achieving precise control over the size distributions of the metal NPs, but also have failed to produce strong attachments between the catalysts and the support materials. Furthermore, the weak metal–support interactions were often followed by poor stability of the catalyst NPs and hence leading to poor lifetime of the whole catalytic system.

In recent studies, “ex-solution process”, in which the NPs are extruded from the support material itself via heat treatment under partially reducing condition, is receiving immense interest for its superior size uniformity, and stability.^496^ In particular, the ex-solved NPs that are strongly attached to the metal oxide support are exceptionally resistant toward agglomeration and contamination (e.g., coking or sulfidation),^497−500^ which is advantageous in chemiresistive gas sensing that requires high-temperature operations and exposure to various gas species. Such aspects of the ex-solved NPs are derived from the fact that the ex-solved NPs originate from the highly reducible transition metal cation dopants in complex oxide lattice, that are selectively precipitated onto the surface of the metal oxide support during partial reduction process.^501^

**Figure 32:**
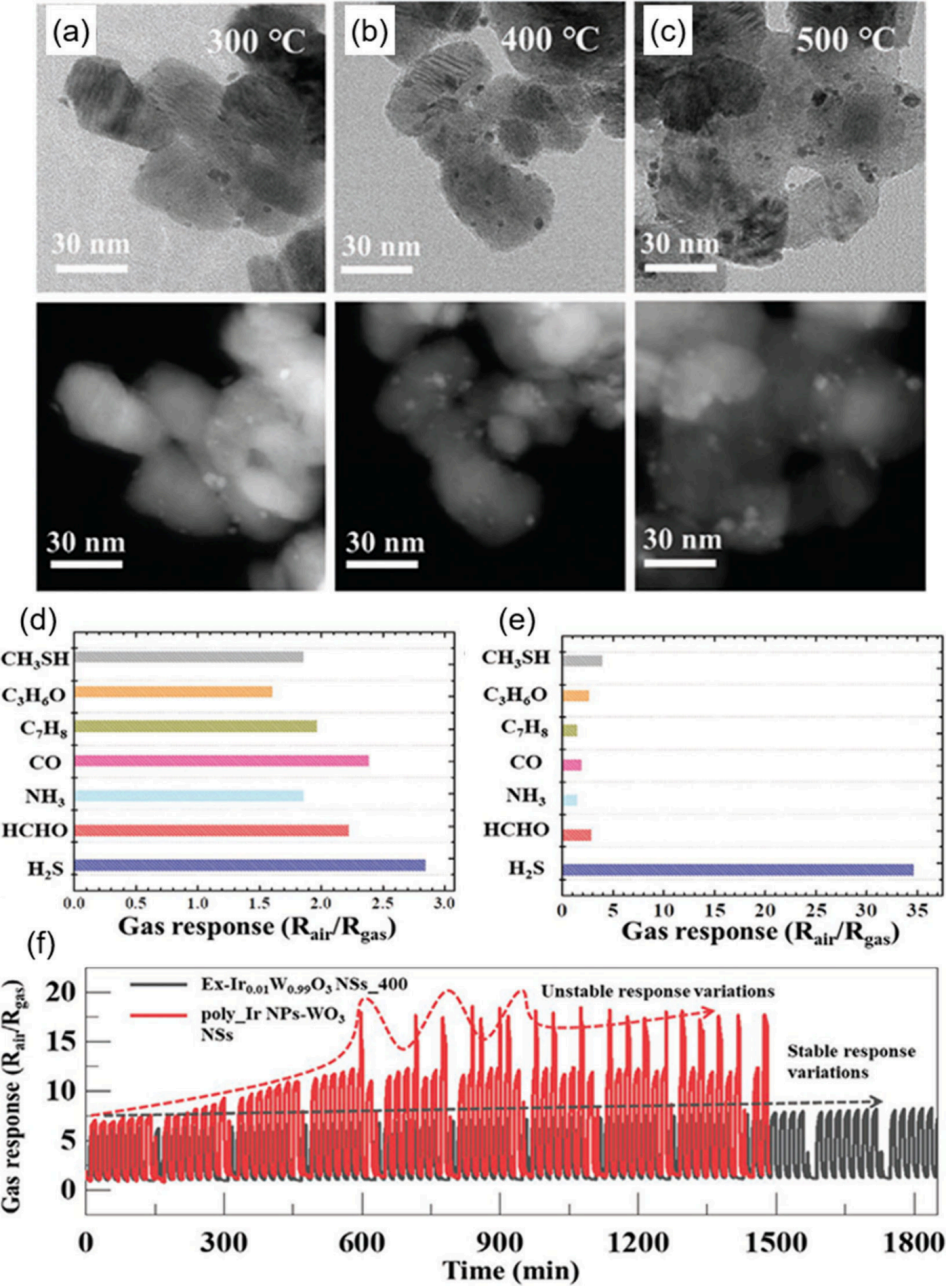
(a–c) In situ TEM images of Ir_0.01_W_0.99_O_3_ NSs annealed in H_2_/Ar (4%/96% volume ratio) at 300 °C, 400 °C, and 500 °C, respectively, selectivity of (d) Ir_0.01_W_0.99_O_3_ NSs and (e) Ex-Ir_0.01_W_0.99_O_3_ NSs_400 toward interfering gas species, (f) 80 cyclic exposure and recovery toward 1 ppm of H_2_S gas for Ir_0.01_W_0.99_O_3_ NSs and Ex-Ir_0.01_W_0.99_O_3_ NSs_400. Reproduced with permission from ref (502). Copyright 2020 Wiley-VCH.

As a first ever demonstration to employ ex-solved metal NPs as a catalyst for chemiresistive gas sensors, Jang et al. used Ir NPs-ex-solved WO_3_ nanosheets (NS).^502^ Here, interestingly, the ex-solution phenomenon was achieved at a temperature as low as 300 °C (Figure 32a–c), whereas in typical ex-solution processes the reductive heat treatments are carried out at high temperatures of >700 °C. This work highlights the role of Ir doping on host WO_3_ to promote dynamic phase transition of WO_3_ during the ex-solution process by ‘breaking’ the symmetry of the host oxides. Then, this phase transition destabilizes the Ir cation dopants, thereby accelerating the extrusion of Ir as nanoparticles at the surface of WO_3_.

In comparison with Ir-doped WO_3_ NS sample (Ir_0.01_W_0.99_O_3_) before the ex-solution process, WO_3_ NSs with Ir NPs ex-solved at 400 °C (Ex-Ir_0.01_W_9.99_O_3_ NSs_400) showed far superior gas sensing response toward H_2_S gas at 375 °C, with the response value (R_air_/R_gas_) of 35 at 5 ppm, which is over 10-fold higher than that of Ir-doped WO_3_ NSs. While the response toward H_2_S gas molecules has experienced a substantial improvement, the responses of Ex-Ir_0.01_W_9.99_O_3_ NSs_400 toward interfering gas species such as CO or NH_3_ remained unchanged (Figure 32d–e). Furthermore, the ex-solved NPs are known to have extremely high sinter-resistance, as mentioned earlier, and Jang et al. have successfully demonstrated it through comparative experiments against WO_3_ NSs decorated with polyol-synthesized Ir NPs, by exposing both samples to 80 cycles of H_2_S (Figure 32f). Overall, this case study presents excellent evidence that the ex-solved NPs can provide enhancements in selectivity in metal oxide-based chemiresistive gas sensors that are identical to the conventional NPs, but the far superior durability, stronger attachment to the host metal oxide, and the finer distribution of the ex-solved NPs will undoubtedly provide a new paradigm in the supported catalyst designs for gas sensors.

In a more recent study by Park et al., have suggested an alternate route for low temperature ex-solution (400–500 °C) by employing metal–organic framework (MOF) as an oxide precursor.^503^ The high surface area and mesoporosity of MOF not only provide uniform stabilization sites for incorporated metal cations, but also forms a metal oxide with extremely high specific surface area when calcined in air. Owing to the abundant active binding sites and the successfully ex-solved bimetallic NPs (combinations of Pt, Pd and Rh), the MOF-derived mesoporous ZnO particles functionalized with ex-solved NPs, PdPt in particular, have shown excellent acetone gas sensing properties. In terms of selectivity, ex-solution of PdPt NPs has significantly improved the response of ZnO particles toward acetone gas exclusively, while the responses toward other interfering gases were hardly affected.

Ex-solution has obvious advantages for catalyzing metal oxide-based chemiresistive gas sensors over those of conventional synthetic approaches for metal NPs, in that they can offer generally higher uniformity, dispersity, sinter resistance, and chemical/thermal stability. Since the phenomenon was fairly recently discovered, there still are plenty of opportunities for selectivity enhancement via attempting new combinations of metal elements for the NPs, the support metal oxides, as well as the morphologies of the support metal oxide especially in the realm of nanostructure engineering. Furthermore, one of the key challenges in ex-solution that needs to be overcome is that a significant proportion of the cation dopant remains in the support oxide lattice after the ex-solution process, and therefore cannot participate in the surface catalytic reactions. This problem could be alleviated by utilizing nanometer-sized metal oxide nanograins and introducing mesopores, thereby shortening the diffusion length of the metal cations, as was the case in the above study by Park et al. Topotactic ex-solution would provide a more sophisticated solution, where, instead of doping the whole metal oxide support, the metal element intended for ex-solution can be coated only on the support material surface as a thin layer followed by oxidation.^504,505^ If the support oxide is a perovskite, the layered metal can readily form alloyed nanoparticles upon the ex-solution process, which can bring about further enhancements in the response and selectivity for gas sensors.

**Figure 33:**
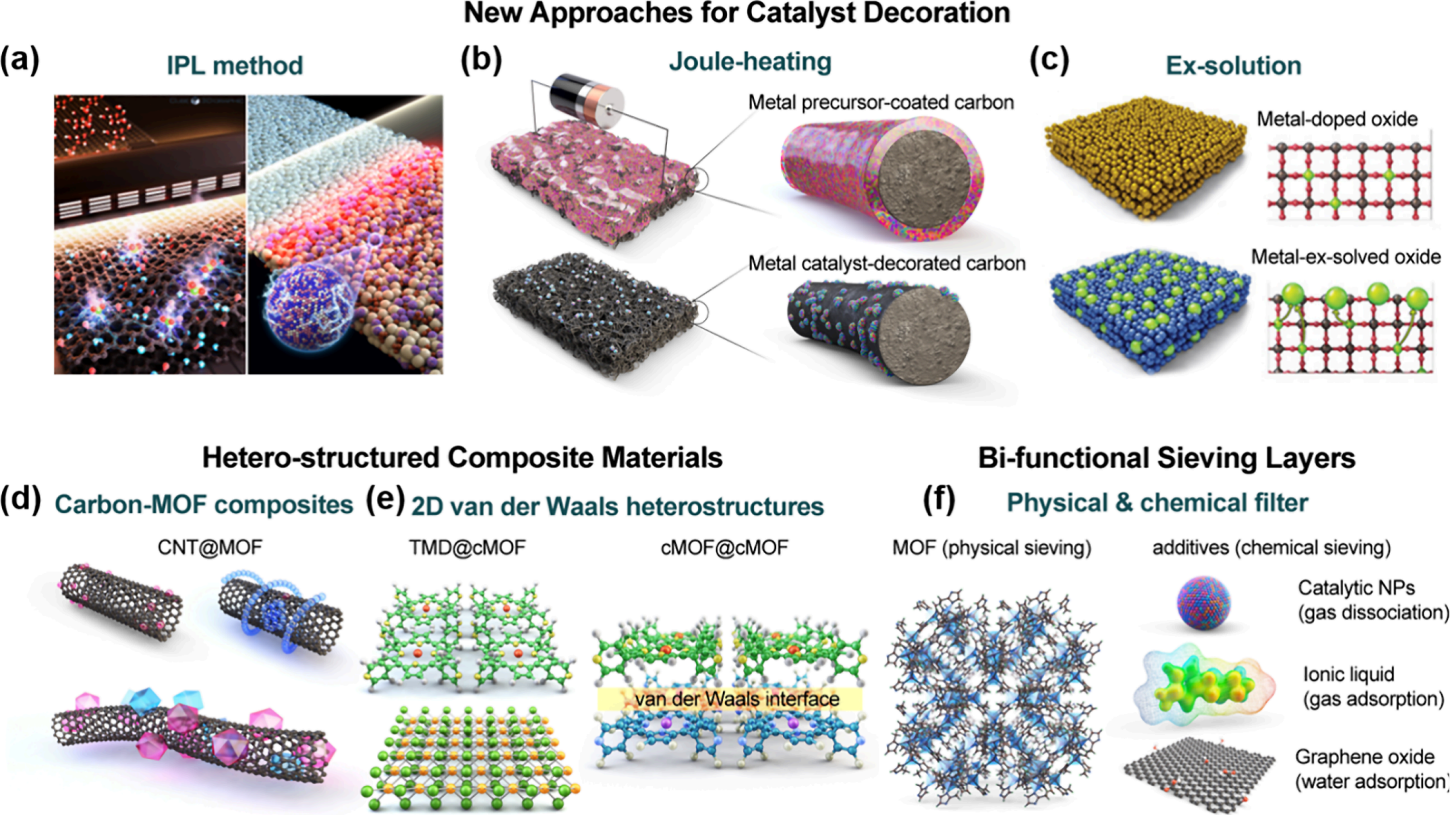
Schematic illustrations of the perspectives of future research directions via new approaches for (a–c) catalyst decoration, (d–e) heterostructured composite materials, and (f) bifunctional sieving layers.

In summary, we introduced innovative catalyst synthesis routes to develop highly sensitive and selective gas sensors. Conventional approaches for the decoration of catalytic NPs on oxide supports utilize wet-chemistry and pyrolysis-involved methods.^506−508^ In particular, pyrolysis normally requires high-temperature annealing for prolonged times which can induce the undesirable phase-separation of multicomponent elements and the agglomeration of catalysts into larger NPs.^482,509^ In this regard, it is challenging to control the composition, uniformity, and size of catalytic NPs, all of which are critical factors for gas sensing performance. Such issue has been attempted to be addressed by using momentary high-temperature annealing methods to directly synthesize and functionalize catalytic NPs on metal oxide supports (Figure 33a,b),^492,510^ and by fabricating strongly ‘socketed’ on oxide supports, so-called ex-solution catalysts, as effective catalysts for tuning gas sensing selectivity (Figure 33c).^496,511^ In this regard, not only high sensitivity and selectivity but also long-term stability characteristics could be obtained by introducing the two novel catalyst-design strategies.

### 7.2 New Heterostructured Composite Materials

Elaborate manipulation and heterostructuring of diverse composite materials could induce unexpected physicochemical properties that could lead to highly sensitive and selective gas sensing performances. In this respect, many researchers have focused on synthesis of sensing layers using composite materials of oxides, carbons, TMDs, and further functionalization with catalytic NPs to improve the sensing characteristics.^512−514^ However, given that new materials have been constantly discovered with intrinsic surface activities, we believe that there remains much room to explore new combinations of composite gas sensing layers.

#### 7.2.1 Carbon Nanotubes–MOF Composites

Carbon nanotubes (CNTs) with tunable electrical conductivity, i.e., either metallic or semiconducting depending on the chirality, promise a number of applications including catalysis, supercapacitors, and chemical sensing based on intrinsic electrical/thermal conductivity, strength, and high surface-to-volume ratio.^515−517^ However, pristine CNTs-based chemical sensors often lack gas selectivity due to their inertness under normal conditions, thus increasing the demand to introduce various functionalization strategies on CNTs.^513,518^ Meanwhile, MOFs constructed by crystalline materials based on coordination chemistry of metal ions and organic ligands feature exceptional chemical and physical properties with large surface area, high porosity, tunable pore size, and tunable active sites.^519^ However, a majority of MOFs, except for cMOFs, often lack in electrical conductivity and stability, hampering their wide use in electrical chemiresistive gas sensing applications. To address the issue, many researches have been reported in which CNTs-MOFs composites are synthesized, taking advantages of the merits of both CNTs and MOFs (Figure 33d). Covalently or noncovalently modified CNTs can be successfully functionalized with MOFs via direct mixing, self-assembly, and multistep synthesis.^520,521^ In the case of covalently modified CNTs, chemical groups are covalently attached onto the framework of CNTs via chemical bonds on which these groups provide active sites for the nucleation and growth of MOFs.^520,522^ Thus, covalent modification with MOFs may decrease the intrinsic conductivity of CNTs. However, gas adsorption on MOFs can directly influence conductivity of underlying CNTs, leading to high resistance variations. Besides, the noncovalently modified CNTs generally utilize wrapping of molecules, e.g., polymers and surfactants, around CNTs through π–π staking interactions.^523^ The functionalities of these molecules can participate in the nucleation and growth of MOFs and they minimize the destruction of the π-network of CNTs, maintaining the structure and electronic properties of CNTs. By using the aforementioned functionalization methods, a variety of CNTs-MOFs composites have been developed to endow with gas selectivity with dramatically improved sensing performances compared to pristine CNTs.^524−526^ However, besides ZIF-based nonconducting MOFs, new strategies for functionalizing conducting MOFs could be interesting ideas to explore to induce unexpected properties of materials and to modulate the corresponding gas sensing characteristics. Layer-by-layer assembly of metal centers and organic ligands could successfully functionalize MOFs on sensing scaffolds such as CNTs with tunable MOF thicknesses and compositions.^456,527,528^ In addition, deprotonation and attaching of diverse organic ligands and/or metals on the surface of CNTs functionalized with −NH_2_, −OH, or −COOH groups could effectively tune the electrical and chemical properties of CNTs to induce enhanced surface activities toward gas analytes.

#### 7.2.2 2D van der Waals Heterostructures

2D van der Waals heterostructures, especially those that utilize TMDs and graphene, have attracted significant interest in gas sensing fields because of their unique properties. However, most 2D materials possess gas selectivity toward NO_*x*_ or NH_3_ gas analytes, thereby hindering the broad application of 2D van der Waals heterostructures in chemiresistive gas sensors.^529−531^ Recently, MOFs-based 2D materials have been introduced as effective gas sensing or sieving layers as described previously (see details in sections 3.4 and 6.4). In particular, introduction of MOF-on-MOF thin film has strong potential feasibility of controlling the gas selectivity based on different gas adsorption energy on metal centers of MOF. For example, introduction of electrically nonconductive 2D MOF (Cu-TCPP) sieving layer on electrically conductive MOF (Cu-HHTP) sensing layer could tune the gas selectivity from NH_3_ to benzene via strong interaction between Cu^2+^ metal centers of Cu-TCPP and NH_3_ gas analytes.^456^ Since there are various types of 2D materials including TMDs, cMOFs, Mxene, black phosphorus, and graphene, there is strong potential feasibility to broaden the types of target gases via combinations of 2D van der Waals heterostructures. Diversification in 2D materials and control of stacking sequences or stacking numbers are two critical engineering factors enabling the control of gas sensing characteristics (Figure 33e).

### 7.3 Bifunctional Sieving Membranes

#### 7.3.1 Metal–Organic Frameworks with Various Additives

As described in section 3.3, many researchers have focused on the introduction of gas sieving membranes on underlying sensing layers, especially by using MOF and zeolites-based physical sieving membranes, oxide-based catalytic filter membranes, or activated-carbon-based sorption-type sieving membranes. However, the multiple interdependent factors that determine the sieving properties of particular systems are not well understood, and this has impeded progress toward the development of bifunctional gas sieving membranes. We believe there are many opportunities for an elaborate combination of physical, catalytic, and chemical filters on the same sensing layer (Figure 33f). Incorporation of catalytic NPs inside the micropores of MOF structures could induce not only the physical screening effect by specific aperture size of MOF structures, but also catalytic filtering effect by dissociation of interfering gas molecules by highly active catalytic NPs. Meanwhile, incorporation of ionic liquids in MOF structures could be an intriguing idea to endow with both physical and chemical sieving effects, since ionic liquids can effectively control the pore size of MOF by partial blockage of the pore to control the effective pore size, as well as adsorb polar gas molecules to induce chemical sieving effect.^532^ It is known that phosphonium-based ionic liquids show high solubility toward sulfur-containing molecules, while imidazolium-based ionic liquids present high solubility toward CO_2_ gas molecules.^533^ Since there are a variety of combinations of ionic liquids (cation and anion parts), diverse MOF-ionic liquids composite materials could be suggested for finely tuned gas selectivity.^534^

Meanwhile, graphene oxide-based sieving membranes possess high adsorption properties toward water molecules, thus direct growth of MOF particles on graphene oxide membrane could induce physical sieving and water molecule capturing effects simultaneously.^218^ If underlying sensing layers are susceptible to oxidation, introduction of water-capturing sieving membranes could greatly improve the long-term stability with highly selective sensing properties. Since the research on gas sieving membranes is at the beginning stage compared to the research on gas sensors, innovative functional sieving membranes could be potentially incorporated on gas sensing layers to control the gas selectivity.

### 7.4 Toward Next-Generation Artificial Olfaction

#### 7.4.1 Multimodal Sensing for Accurate Detection of Gas Molecules

**Figure 34:**
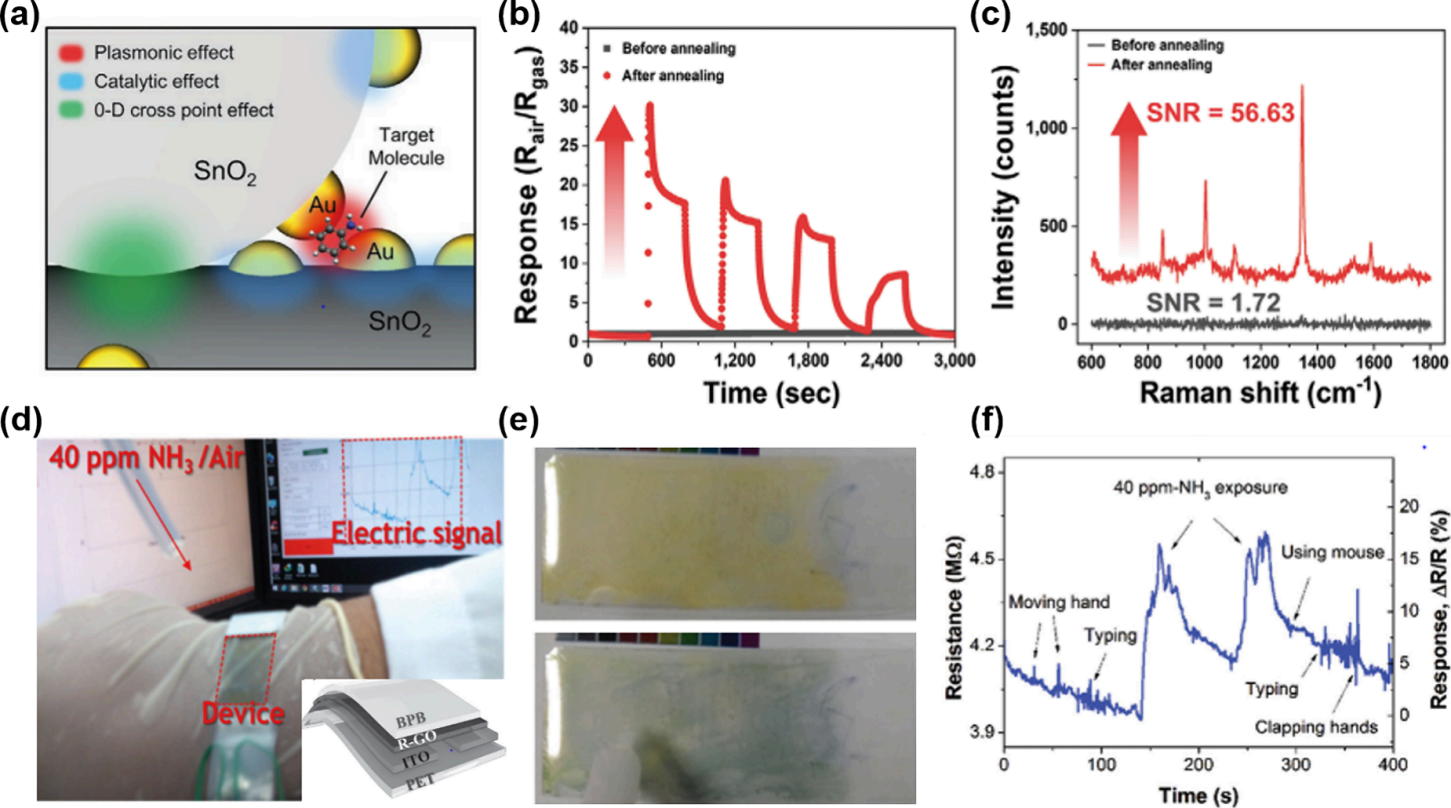
(a) Schematic illustration of the chemiresistive and SERS multimodal sensing platform with Au NPs decorated SnO_2_ of 3D cross-point multifunctional architecture. (b) Chemiresistive and (c) SERS sensing properties with Au NPs decorated SnO_2_ nanowires. Reproduced with permission from ref (535). Copyright 2021 Wiley-VCH. (d) Photograph image of the colorimetered dye-loaded rGO sensing platform and their (e) colorimetric and (f) chemiresistive sensing characteristics upon exposure to NH_3_ gas. Reproduced with permission from ref (536). Copyright 2016 Wiley-VCH.

To overcome the disadvantages of the single-mode sensor platforms in terms of accuracy in quantitative and qualitative analyses, it has been of great interest to develop multimodal sensing platforms by integration of diverse modes of sensors such as chemiresistive, surface enhanced Raman scattering (SERS), and colorimetric to enhance the quality of the output data. In the case of metal oxide-based chemiresistive-type sensors, gas selectivity is relatively poor compared to their gas sensitivity. Meanwhile, colorimetric-type sensors feature high selectivity, but the detection limit is relatively high. The SERS-type sensors have advantages of excellent selectivity and ultralow detection limit characteristics. Recent researches suggest the potential feasibility of building a sensor platform that can compensate for the limitations of each sensor signals by implementing a multimodal (chemiresistive and SERS or chemiresistive and colorimetric) sensing platform.^535^ Han et al. reported a synergistic integration of chemiresistive and SERS sensing for label-free multiplex gas detection. They demonstrated a synergistic multimodal sensing platform that consists of core-metal oxide nanowires for quantitative detection of chemical species using electrical sensing mode and shell-metallic Au NPs for selective identification of chemical species using SERS sensing mode (Figure 34a). In particular, Au NPs serve a dual function of chemical sensitizer to improve the gas response of the electrical signals and plasmonic particles to amplify the Raman signals (Figure 34b,c). In this regard, the suggested multimodal sensor successfully demonstrated highly selective and quantitative identification of trace amount of target gas species. As a chemiresistive and optical dual-mode gas sensor platform, Duy et al. reported flexible transparent reduced graphene oxide sensing layers coupled with organic dye molecules for the rapid detection of ammonia gas molecules (Figure 34d).^536^ In particular, protonated organic dye molecules (bromophenol blue, BPB) chemically react with ammonia gas to form BPB-O^–^NH_4_^+^ or O^–^SO_2_–BPB = ONH_4_^+^ to induce yellow-to-blue color change (Figure 34e). Meanwhile, reduced graphene oxide acts as a p-type semiconducting sensing layer to induce an increase of resistance upon exposure to reducing ammonia gas molecules (Figure 34f). Simple stacking of BPB dyes on reduced graphene oxide sensing layers shows synergistic effects in sensing ammonia gas by offering effective modulation of both electrical and optical signals.

#### 7.4.2 Sensor Arrays, Multianalyte Quantification, and Pattern Recognition Using Principal Component Analysis (PCA) and Deep Learning

In some cases, no sufficiently selective single sensor may be found to discriminate the target gas sufficiently accurate in a mixture of complex gas analytes (e.g., exhaled breath contains >1400 compounds).^537^ When sensors with limited selectivity are combined, their response patterns can describe complex mixtures of gases. This concept is the primary approach for developing electronic noses (E-noses) to detect complex odors, mimicking the human olfactory system in certain aspects.^538,539^ Each olfactory receptor cell, which contains only one type of receptor, can detect a limited range of odorant molecules. For complex odors composed of multiple molecules, several receptors are activated simultaneously. The resulting receptor pattern determines our perception of the odor. Similarly, gas sensors that are individually selective and sensitive to specific gases function as receptors. These sensors are then arrayed in the E-nose system to produce distinct signal patterns to discriminate complex gas analytes.^540,541^ The pattern is then analyzed both quantitatively and qualitatively, analogous to the recognition process in the olfactory cortex of the brain. To achieve this, various pattern recognition algorithms have been developed using machine learning, further accelerated by recent advancements in neural networks.^542,543^ Commonly used algorithms classifiers include linear discriminant analysis (LDA), *k*-nearest neighbors (KNN), classification and regression trees (CART), Gaussian naïve Bayes (NB), support vector machines (SVM), random forest (RF), multilayer perceptron (MLP), convolutional neural network (CNN), and recurrent neural network (RNN).^543,544^ The potential applications of the E-nose sensor system include exhaled breath analysis,^229,545,546^ air-quality monitoring,^547^ and food-contamination test.^548^ However, since the concentration of the target gas in the complex gases can be extremely low, down to ppb level, manufacturing highly sensitive sensors capable of detecting extremely low-concentration gas with high selectivity is an important task to be resolved, prior to building the multiarray sensors system.

Complex gas mixtures can be statistically analyzed by introducing a data processing tool such as principal component analysis (PCA).^549^ Note that the PCA is a data processing procedure based on statics, which can summarize the large amount of data into smaller amount via dimension reduction and data compression steps. Then, only sensor response patterns are identified that might be discriminant in some situations (e.g., healthy vs lung cancer from exhaled breath),^270^ but the information on the underlying gas molecules is lost or not extracted.^550^ This “black box” approach bears the risk of pseudo correlations. Specifically, due to the moderately selective and widely sensitive nature of the sensors used in E-noses, response patterns may be influenced or even generated by confounding factors.551 In this article, we have suggested pathways to design highly selective sensors that are less susceptible to confounders. These sensors enable sensor arrays with more orthogonal characteristics, that feature enhanced discrimination power and robustness, when applying, for instance, multivariate regression algorithms for data analysis.^552^ As a result, individual gas molecule types can be quantified directly in complex mixtures, as will be demonstrated below with selected examples.

**Figure 35:**
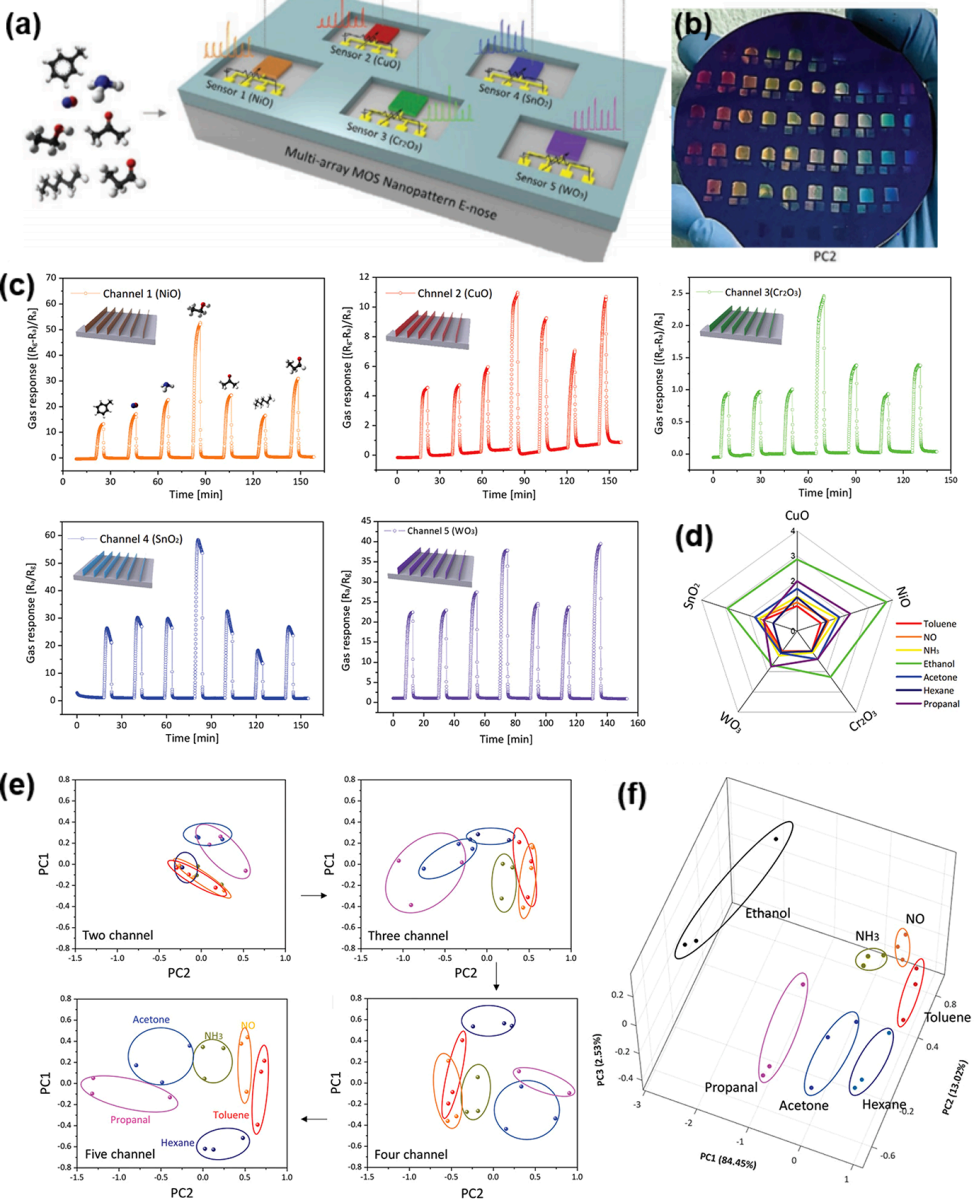
(a) Conceptual schematics of detecting various gas analytes using multiarray gas sensors and (b) photography image of fabricated multiarray nanochannel in 4 in. wafer scale. (c) Sensing response of five different oxide-based channels toward 5 ppm of seven different gas analytes (toluene, nitrogen monoxide, ammonia, ethanol, acetone, hexane, and propanal). (d) Selectivity fingerprints of five channels toward seven different gas analytes. (e) 2D PCA results to discriminate six different gas analytes using two, three, four, and five channels, respectively. (f) 3D PCA results to discriminate seven different gas analytes using five channels. Reproduced with permission from ref (557). Copyright 2020 Wiley-VCH.

Gas selectivity can be endowed with by controlling (i) the type of metal catalysts functionalized on metal oxides^553,554^ and/or (ii) the type of metal oxide support materials.^555,556,554,555^ In particular, in terms of the fabrication process, facility, reliability, and throughput are important factors to be considered for commercialization. In this regard, Kang et al. proposed the successful fabrication of multiarray nanopattern E-nose system via high-resolution top-down nanolithography methods.^557^ Thin-walled (20–30 nm) and nanogranular (5 nm) patterns of five different metal oxides including NiO, CuO, Cr_2_O_3_, SnO_2_, and WO_3_ were fabricated into multiarray sensors via high-throughput lithography method that utilizes sputtering of the metals using low-energy ion plasma bombardment (Figure 35a). The proposed plasma bombardment-based top-down lithographic approach is advantageous in terms of its processability; 54 individual sensing devices were fabricated in a 4-in. wafer scale, simultaneously, proving unique potential for large-scale fabrications (Figure 35b). The sensing tests were conducted using NiO, CuO, Cr_2_O_3_, SnO_2_, and WO_3_ channels toward 5 ppm of toluene, nitrogen monoxide, ammonia, ethanol, acetone, hexane, and propanal gas analytes (Figure 35c). Four channels including NiO, CuO, Cr_2_O_3_, and SnO_2_ exhibited the highest response toward ethanol vapor, while WO_3_ channel showed strong reactivity toward propanal vapor (Figure 35d). Although the response values of CuO and Cr_2_O_3_ are much lower than those of NiO, SnO_2_, and WO_3_ channels, when integrated into E-nose systems, all five different sensors provide important clues of fingerprints left by these gases. In this regard, the pattern recognition was conducted using PCA to successfully discriminate and distinguish seven different types of gases. The PCA results indicate that increasing the number of channels is an important factor to clearly distinguish patterns from multiple gases; two to four channels insufficiently discriminate multiple gases, while five channel system successfully distinguish all the seven different gases into nonoverlapping regions (Figure 35e,f). These results demonstrate the importance of the number of channels when it comes to building-up a E-nose sensor platform to selectively discriminate mixed gas analytes. More sensor channels with individually distinct signals provide more accurate fingerprints of specific gases, but it is also important to process the massive data meaningfully.^558,559^

In another study, an array of four differently doped SnO_2_ sensors was applied.^560^ The individual sensors detected formaldehyde down 3 ppb with high signal-to-noise ratio (>25) at 90% relative humidity. When tested with gas mixtures including ammonia, ethanol, and acetone, formaldehyde (30–180 ppb) was quantified accurately with average error ≤ 9 ppb. Yet, this error increased with increasing complexity of the gas mixture due to the rather colinear characteristics of the applied sensors. This was addressed by utilizing more selective sensors enabling arrays with more orthogonal characteristics. This was demonstrated under real conditions when quantifying multiple analytes simultaneously in mixtures with hundreds of constituent gases. For instance, a sensor array consisting of distinct selective sensors including Ti-doped ZnO, Si-doped MoO_3_ and Si-doped ε-WO_3_ quantified breath- and skin-emitted ammonia, acetone and isoprene accurately (error ≤ 21 ppb) from humans when entrapped in a plethysmography chamber.^561^

**Figure 36:**
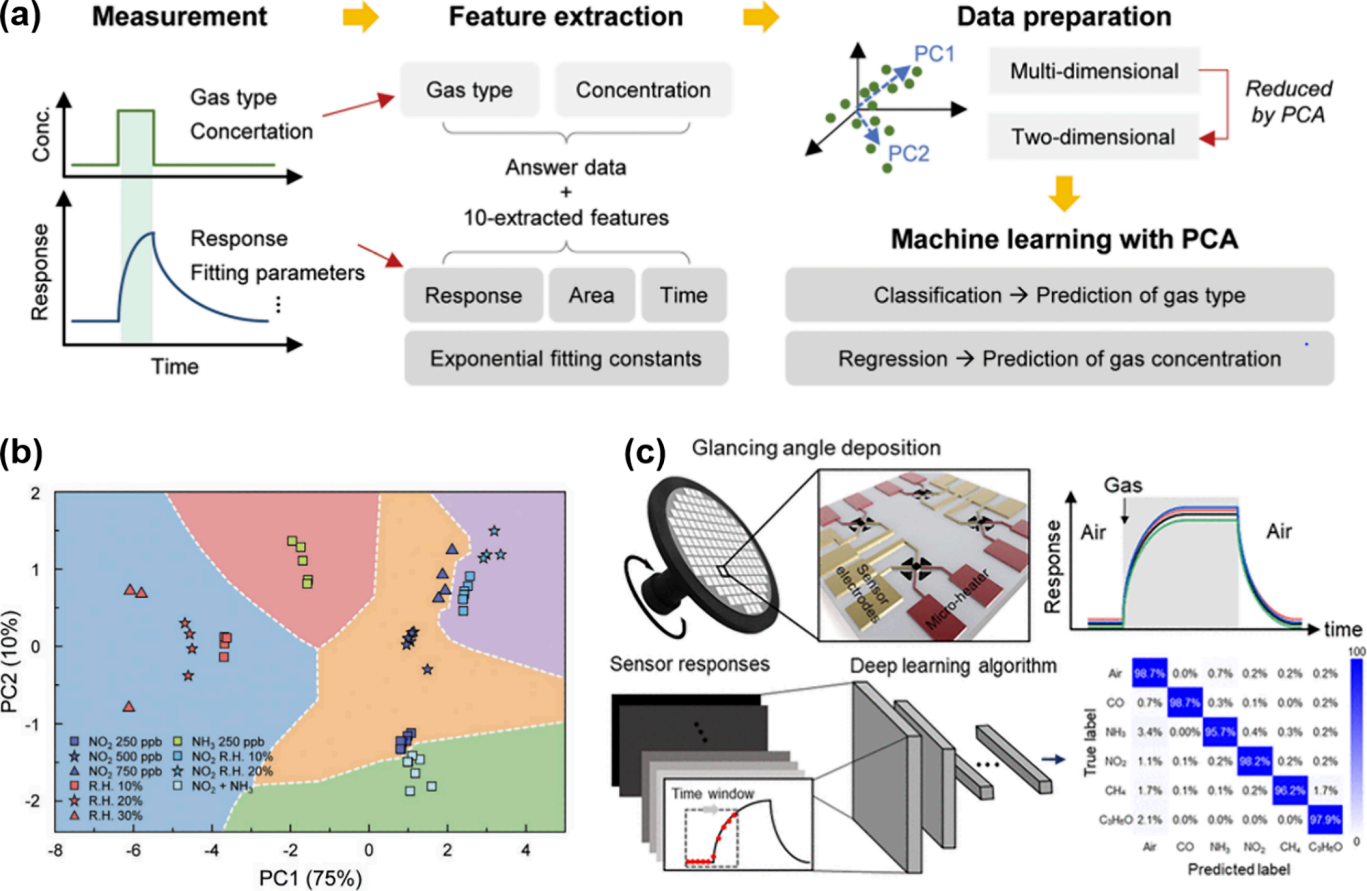
(a) Schematic illustration of the PCA-assisted machine learning classification of the data preparation. (b) PCA-assisted machine learning classification and regression. Reproduced with permission from ref (570). Copyright 2022 Wiley-VCH. (c) Preparation of uniform gas sensor arrays and applying the machine learning algorithm for high accuracy identification of multigas species. Reproduced with permission from ref (565). Copyright 2022 American Chemical Society.

Machine learning or deep learning is an ultimate data processing tool to classify different types of gases based on the collected sensing data from multiarray sensors.^540,543,551,562,563,540,543,550,561,562^ With the balance of the development of advanced algorithm system using deep learning and high-performance sensing layers, the multiarray E-nose sensor platform could possibly classify low concentration mixed gases with fast computation speed and the accuracy. Interestingly, Ogbeide et al. reported highly predictable detection of NO_2_ and humidity concentrations in a mixed gas atmosphere by adopting machine learning on single sensor which consists of inkjet-printed rGO/CuCoO_*x*_-based sensing layers.^564^ The data acquisition process accompanies extraction of ten-characteristic parameters from the gas sensing measurement data upon exposure to target gases, which were then reduced to two principal components for the machine learning which includes classification and regression steps (Figure 36a). Note that the classification and regression predict the gas type and gas concentration, respectively. From the PCA-assisted machine learning data, ten different sensing results can be clearly discriminated (Figure 36b). As a result, the proposed sensing platform allows for 98.1% accuracy for the prediction of NO_2_ concentration and humidity. This work successfully demonstrated the accurate detection of two types of unknown gas concentrations using a single sensor device, suggesting potential feasibility to expand to detection of complex gas molecules by introducing multiarray sensor platforms. Kang et al. suggested highly accurate identification of multigas species using uniform metal oxide-based gas sensor arrays and introducing the deep learning algorithm to the response data obtained from the sensor arrays (Figure 36c).^565^ They proposed glancing angle deposition method to prepare uniform metal oxide sensors including SnO_2_, In_2_O_3_, WO_3_, and CuO with low batch-to-batch variation in response (a relative standard deviation of ∼5%), and adopted convolutional neural network on multi gas sensor arrays to enable highly accurate (98%) identification of gas mixtures including CO, NH_3_, NO_2_, CH_4_, and C_3_H_6_O. Recently, Sung et al. proposed using eigengraphs in deep learning models as refined representations derived from sensing graphs to effectively characterize different gas reactions.^566^ This data-centric approach, combined with an olfactory receptor-like sensor array, enables accurate classification (96.1–100%) of mixtures of automobile exhaust gases, including CO, CO_2_, NO, NO_2_, C_3_H_8_, and O_2_. On the other hand, the high processing capability of machine learning algorithms enables the utilization of additional sensing data that was previously neglected or unused. For example, transient signals generated from external stimuli, such as rapid heating cycles or pseudorandom illumination, can provide additional information depending on the analytes.^567,568^ Additionally, SMO sensors can be integrated with neural transistors to produce distinct spiking signals for different gases.^569^ These additional signals can be analyzed using machine learning, enhancing the capability of e-Noses to distinguish gases more accurately and efficiently.

The future of machine learning in gas sensing is promising, with ongoing research focused on developing sophisticated sensor arrays coupled with advanced machine learning models.^571^ Machine learning has emerged as a transformative tool for the design and optimization of materials and structures, particularly in metal oxide-based chemiresistive sensors, where catalytic activity plays a critical role in gas sensing performance. Machine learning enables efficient screening of multicomponent catalysts, which have demonstrated unexpectedly high catalytic activity for specific target gases, significantly reducing the experimental workload required to explore the vast combinations of catalyst compositions.^494,572^ Recent advancements, such as the integration of experimental trials with active learning, have further enhanced the optimization process for multimetallic alloy catalysts, successfully identifying optimal precursor compositions for high-performance catalysts.^573^ This approach is particularly effective in designing and synthesizing advanced catalysts tailored for gas sensing applications, enhancing selectivity for specific gases while minimizing experimental costs and time.

Beyond catalyst design, machine learning and artificial intelligence systems, such as ChatMOF, demonstrate significant potential in advancing materials research.^574^ ChatMOF utilizes large language models to predict and generate metal–organic frameworks (MOFs) with high accuracy. Similarly, biomimetic olfactory chips featuring monolithically integrated sensor arrays on nanoporous substrates, with up to 10,000 individually addressable sensors per chip, leverage AI to achieve high selectivity to various gases, distinguish mixed gases, and detect 24 distinct odors.^575^ These systems aim to accurately detect and quantify complex gas mixtures in real-time, with applications ranging from environmental monitoring^576^ to medical diagnostics.^577^ Researchers are working toward creating sensor arrays capable of identifying and quantifying over ten gas types with at least 98% accuracy for air quality monitoring. In the medical field, there is significant potential for detecting biomarker gases at parts-per-billion (ppb) levels with high accuracy in both qualitative and quantitative assessments, potentially leading to breakthroughs in noninvasive disease diagnosis and monitoring.^92^ Integrating PCA with deep learning techniques enhances the extraction and interpretation of significant patterns from high-dimensional data sets generated by multisensor arrays. This approach improves the qualitative and quantitative analysis of gas components, enabling more nuanced discrimination of complex gas mixtures. As sensor technology advances, we expect to see more compact, efficient, and versatile gas sensing systems. These systems will likely incorporate a diverse range of sensor types on a single device, providing a wealth of data for machine learning algorithms to process. This convergence of advanced sensing hardware and sophisticated machine learning algorithms promises to revolutionize gas detection and classification across various industries, from environmental monitoring to industrial safety and healthcare.

#### 7.4.3 Future Perspectives in Artificial Olfaction

**Figure 37:**
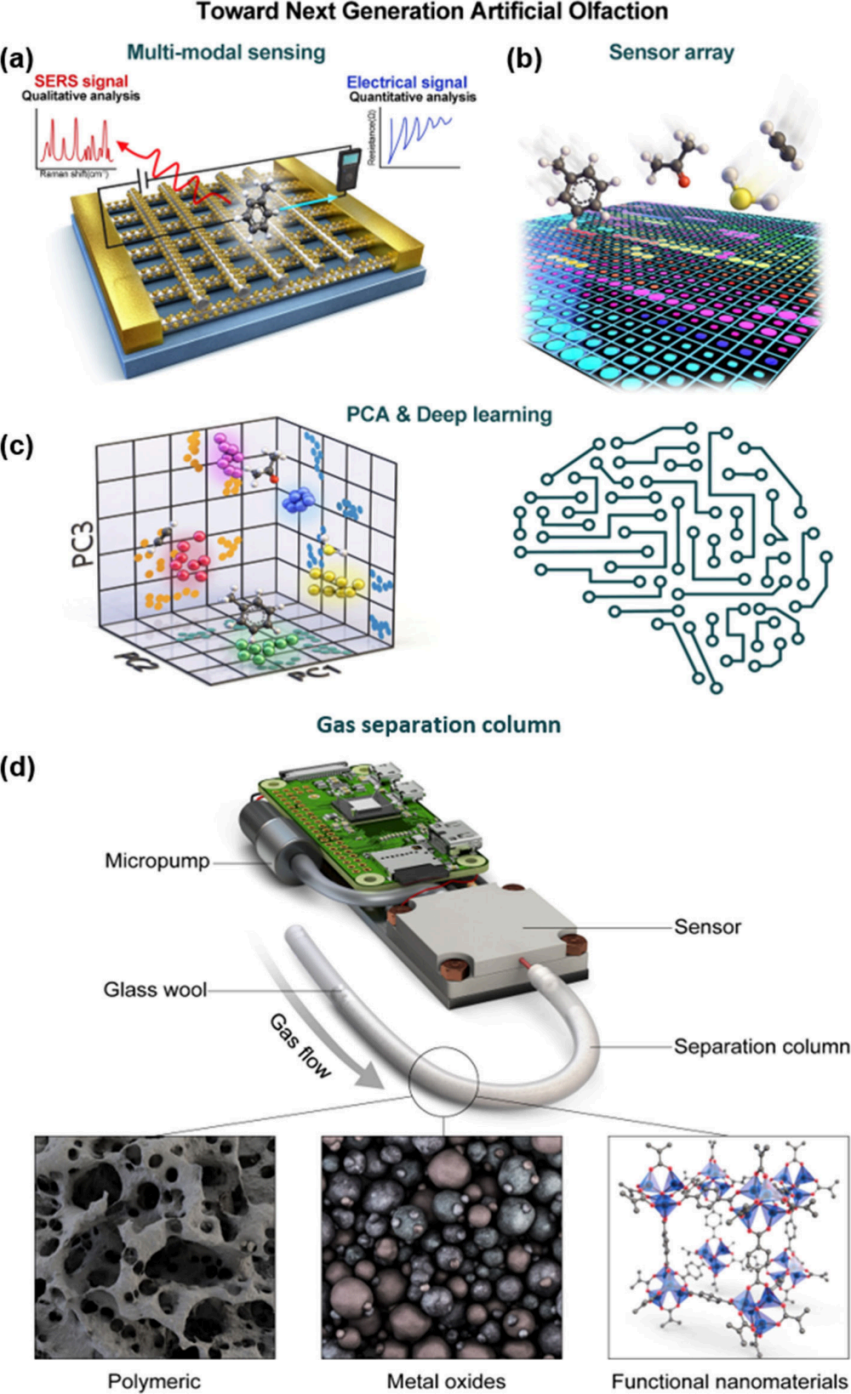
Schematic illustrations of the perspectives for next generation artificial olfaction based on (a) multimodal sensing, (b) sensor array, (c) PCA/deep learning techniques, and (d) gas separation column.

In the previous sections, we discussed future directions for the newly developed highly selective gas sensing materials. In this last section of our review, we suggest as futuristic concepts toward next generation artificial olfaction:

i. Integration of multiple different modes of gas sensors into a multimodal gas sensing system (Figure 37a), which will mutually compensate the limitations that each gas sensing modes suffer from for example, surface-enhanced Raman spectroscopy (SERS) can compensate the low selectivity that the chemiresistive gas sensors have, while chemiresistive gas sensors can compensate the narrow range of detectable gas species that SERS has.
ii. The availability of highly selective gas sensors enables the formation of arrays (Figure 37b) with more orthogonal characteristics. Together with the aid of machine learning and, perhaps pattern recognition (Figure 37c), enables data processing to extract more robust signal patterns and even to discriminate and quantify individual gas molecules with high accuracy. Along with the dramatic leap of the machine learning technologies, we anticipate development of more advanced and accurate sensor array platform in the near future for identifying multiple gases both in qualitative and quantitative manners.
iii. Gas separation columns based on physical/chemical filtration of gas molecules result in complete exclusion or increased retention time to reach the gas sensor, as we have discussed in depth in Figure 12, are currently relying on polymeric and metal oxide filters. We anticipate that the filtration properties of the gas separation columns can be further improved by utilization of functional nanomaterials such as graphene derivatives, metal–organic frameworks, covalent organic frameworks, and many more functional nanomaterials (Figure 37d).

We envision that successful demonstration of the strategies we have listed above will indeed be the goal for ultimate improvement of the selectivity of the chemiresistive gas sensors, and will bring us one step closer to commercialization of gas sensors for universally wide applications. Previous examples have demonstrated that the transfer times from innovation of a selective sensor system to commercial product can be extremely short (e.g., < 2 year)^96^ yielding rather immediate economic and societal impact.

## 8 Summary and Outlook

In this review, we focused on the strategies reported in literature for improving the selectivity of various gas-sensing chemiresistive materials. As observed, various mechanisms depend on factors such as target gas type, properties of materials, influence of catalysts, as well as synthesis and operating conditions. While chemiresistive sensors, especially those based on SMOs, already show modest sensitivity toward various gases, the selective detection of specific gases by single-sensor elements remains challenging due to limited receptor availability and nonspecific absorption when exposed to many analytes. To this end, functionalization with catalysts and/or defect engineering are promising approaches to overcome these drawbacks. Nevertheless, the capability for size control and distribution of catalyst on SMO surfaces are still limited by current technology and synthetic methods, which remains an important topic for future research endeavors.

Besides, despite the significant advancements in material design and functionalization, the evaluation of selectivity in gas sensing remains inconsistent across studies. Current assessments often rely on comparing sensor responses to individual gases in isolation or simple binary mixtures, which may not fully reflect real-world scenarios involving complex gas mixtures and environmental variations. To advance the field, it is imperative to develop standardized protocols that rigorously test selectivity under practical conditions, such as varying humidity, temperature, and the presence of multiple interfering gases.

For single-target sensors, we introduced another equally attractive strategy to improve selective detection of gas species, which is the incorporation of multiple single-target sensors into an array of microsensors on a single chip. With the availability of more selective gas sensors, the robust and simultaneous discrimination between several gases in mixtures and their quantification can be achieved. A critical requirement is that these individual single-sensor elements, cross-sensitive to different gases, must exhibit significantly high sensing responses from each other in a multidimensional space to allow for efficient discrimination of analytes. The basic working principles and viable designs have been demonstrated in several reports as the proof-of-concept.^119,578^ Moreover, the integration of these arrays with machine-learning will enable substantial enhancements in rapid classification of exposed gas species by accumulation of data and pattern recognition.

For the case of metals, nonmetals, and metal-based alloys/composites, unavailability of intrinsic receptors for a variety of gases seems to be the key limiting factor. However, avenues to achieve substantial structural and functional diversity to improve gas receptors for enhanced gas sensing performance still exist. Many researchers propose improvement of metallic alloys/composites by incorporating rare-earth metals in well-researched Pd, Pt, and Si systems, as well as composition control in current systems such as PdNi, PdAu, PtPd, PdMg, and Si/Pd. On the other hand, advanced synthetic techniques can be employed to tailor some 2D materials (for example MoS_2_) to exhibit improved sensitivity, shorter response/recovery, and selectivity to a wide range of gases.^579^

In the case of newly discovered 2D materials like MXenes and phosphorenes, they exhibit tunable functionalities that have triggered many investigations on their chemiresistive sensing properties.^47,580,581^ Specifically, MXenes exhibit tunable electronic properties and terminating groups, and phosphorenes exhibit puckered-like lattice structure and tunable direct band gap. These features offer exciting prospects for exceptional selectivity toward nitrogen-containing gases.^47,582,583^ In particular, while materials such as MXene already exhibit tunable physicochemical properties that are key prerequisites for molecular sieving/selective membranes, they are also permselective to H_2_ against many gases such CO_2_, O_2_, N_2_, propane, methane, and propene,^584^ which enables their additional role as filter layers to achieve a finer optimization of the selectivity. Future investigations will perhaps capitalize on these new insights to incorporate 2D material overlayers onto active sensing surfaces such as SMOs and metal composites.

To address drift and stability challenges in various sensors, researchers have developed diverse strategies tailored to specific materials. For semiconducting metal oxides, ex-solution catalytic nanoparticles prevent agglomeration at high temperatures, ensuring long-term stability, while dynamic surface interactions, such as CeO_2_ on In_2_O_3_, enable humidity-independent behavior.^585^ Carbon-based materials like graphene and CNTs benefit from photoactivation, thermal treatments, or dynamic baseline correction to neutralize trapped charges and restore baseline resistance.^586−589^ For conducting MOFs, introducing light-active nanoparticles or blending with hole-rich polymers enhances reversibility by facilitating the desorption of strongly adsorbed analytes. Additionally, advanced approaches like heterojunction engineering, sieving membranes, and AI-driven dynamic baseline correction provide promising solutions to improve sensor stability and reliability under real-world conditions.

While this review primarily focuses on enhancing the selectivity of gas sensors through material design and engineering strategies, it is worth acknowledging the advancements in separation element miniaturization as a complementary approach.^590^ The miniaturization of chromatographic columns and micropreconcentrators, for instance, can effectively reduce cross-interference and enhance the specificity of gas sensing systems. Future studies combining these separation technologies with selective sensing materials could offer a promising pathway toward achieving high selectivity in real-world applications. A more detailed exploration of this topic would provide a valuable perspective and can be addressed in dedicated reviews focused on separation-sensor integration strategies.

Improved selectivity in chemiresistive sensory devices can greatly expand the functionality of current sensor systems for medical diagnosis applications, environmental monitoring, and safety assurance. Arguably, the most impactful application of highly selective chemiresistive sensors is the noninvasive gas sensing platform in monitoring human health by detecting and analyzing gases in human breath, which are indicative of various noncommunicable diseases.^411,550,591,592^ Ultimately, achieving high selectivity in chemiresistive sensing is a pivotal undertaking in the development of practical chemiresistive sensors that guarantee the health and safety of humans in a vast range of applications.
